# Metabolome‐Based Genome‐Wide Association Study of Duck Meat Leads to Novel Genetic and Biochemical Insights

**DOI:** 10.1002/advs.202300148

**Published:** 2023-04-04

**Authors:** Dapeng Liu, He Zhang, Youyou Yang, Tong Liu, Zhanbao Guo, Wenlei Fan, Zhen Wang, Xinting Yang, Bo Zhang, Hongfei Liu, Hehe Tang, Daxin Yu, Simeng Yu, Kai Gai, Qiming Mou, Junting Cao, Jian Hu, Jing Tang, Shuisheng Hou, Zhengkui Zhou

**Affiliations:** ^1^ Institute of Animal Science Chinese Academy of Agricultural Sciences Beijing 100193 P. R. China

**Keywords:** genetic and biochemical basis, meat flavor and nutrition, metabolites, mGWAS, volatiles

## Abstract

Meat is among the most consumed foods worldwide and has a unique flavor and high nutrient density in the human diet. However, the genetic and biochemical bases of meat nutrition and flavor are poorly understood. Here, 3431 metabolites and 702 volatiles in 423 skeletal muscle samples are profiled from a gradient consanguinity segregating population generated by Pekin duck × Liancheng duck crosses using metabolomic approaches. The authors identified 2862 metabolome‐based genome‐wide association studies (mGWAS) signals and 48 candidate genes potentially modulating metabolite and volatile levels, 79.2% of which are regulated by *cis*‐regulatory elements. The level of plasmalogen is significantly associated with *TMEM189* encoding plasmanylethanolamine desaturase 1. The levels of 2‐pyrrolidone and glycerophospholipids are regulated by the gene expression of *AOX1* and *ACBD5*, which further affects the levels of volatiles, 2‐pyrrolidone and decanal, respectively. Genetic variations in *GADL1* and *CARNMT2* determine the levels of 49 metabolites including L‐carnosine and anserine. This study provides novel insights into the genetic and biochemical basis of skeletal muscle metabolism and constitutes a valuable resource for the precise improvement of meat nutrition and flavor.

## Graphical abstract

Meat is among the most consumed foods worldwide and has a unique flavor and high nutrient density in the human diet. However, the genetic and biochemical bases of meat nutrition and flavor are poorly understood. Here, 48 candidate genes potentially modulating metabolite and volatile levels are identified and provide a roadmap to improve meat nutrients and flavor.

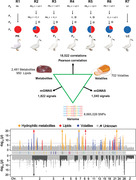

## 1 Introduction

Meat is considered the postmortem skeletal muscle of vertebrate animals,^[^ ^1^, ^2^ ^]^ has played a crucial role in human evolution and is an important component of a healthy and balanced diet due to its highly abundant nutrients.^[^ ^3^ ^]^ As a complex food, meat displays various biochemical properties that are largely determined by a variety of metabolites, including both hydrophilic and hydrophobic metabolites.^[^ ^4^ ^]^ Hydrophilic metabolites and lipids in skeletal muscle not only provide essential nutrition but also, as flavor‐precursor volatiles, determine meat flavor.^[^ ^5^, ^6^ ^]^ Volatiles are essential for good flavor in particular and are generated through complex chemical reactions among various metabolites, such as lipid autoxidation and the Maillard reaction.^[^ ^7^, ^8^ ^]^ Recently, metabolome and volatilome analyses have been widely applied in meat phenotype studies.^[^ ^9^, ^10^, ^11^, ^12^ ^]^ However, the meat metabolome, volatilome, and their correlation have not been systematically studied, which hinders further research and improvement of the nutrition and flavor of the meat.^[^ ^13^, ^14^ ^]^

In recent years, metabolomic analysis coupled with genome‐wide association studies (GWAS) has made it possible to simultaneously screen a large number of genetic loci for important metabolic traits to understand the genetic basis of metabolic diversity and its relevance to complex traits.^[^ ^15^ ^]^ For example, metabolic GWASs (mGWASs) on blood metabolites have been carried out in humans, resulting in the identification of a large number of genetic loci for metabolite concentrations and providing new insight into many disease‐related associations.^[^ ^16^, ^17^, ^18^ ^]^ In plants, a series of studies have been carried out on important crops and fruits, including rice,^[^ ^19^ ^]^ maize,^[^ ^20^ ^]^ wheat,^[^ ^21^ ^]^ tomato^[^ ^22^ ^]^ and peach,^[^ ^23^ ^]^ and have provided an indispensable reference to help understand the genetic basis of metabolomes and to facilitate breeding for enhanced nutritional value. Thus, understanding the genetic basis of the metabolome and volatilome in meat is essential to improve and enhance the nutritional value and flavor of meat for consumers.

Roasted Pekin duck is a world‐famous dish produced from Pekin duck.^[^ ^24^ ^]^ As an elite indigenous breed, Liancheng duck is the most popular among local consumers due to its highly delicious flavor.^[^ ^25^ ^]^ There are dramatic differences between Pekin duck and Liancheng duck in terms of nutrients and flavor (Figure S1, Supporting Information). Here, we constructed a large Pekin duck (high meat yield and subcutaneous fat content) × Liancheng duck (low meat yield and subcutaneous fat content) gradient consanguinity segregating population to profile metabolites in raw meat and volatiles in cooked meat, as well as explored their genetic and biochemical basis. Our study identified a number of QTLs affecting the levels of metabolites and volatiles and provided novel insights into the genetic basis of skeletal muscle metabolic traits. Moreover, our study provided powerful data for meat quality improvement.

## 2 Results

### 2.1 Metabolite Profiling of Skeletal Muscle

**Figure 1:**
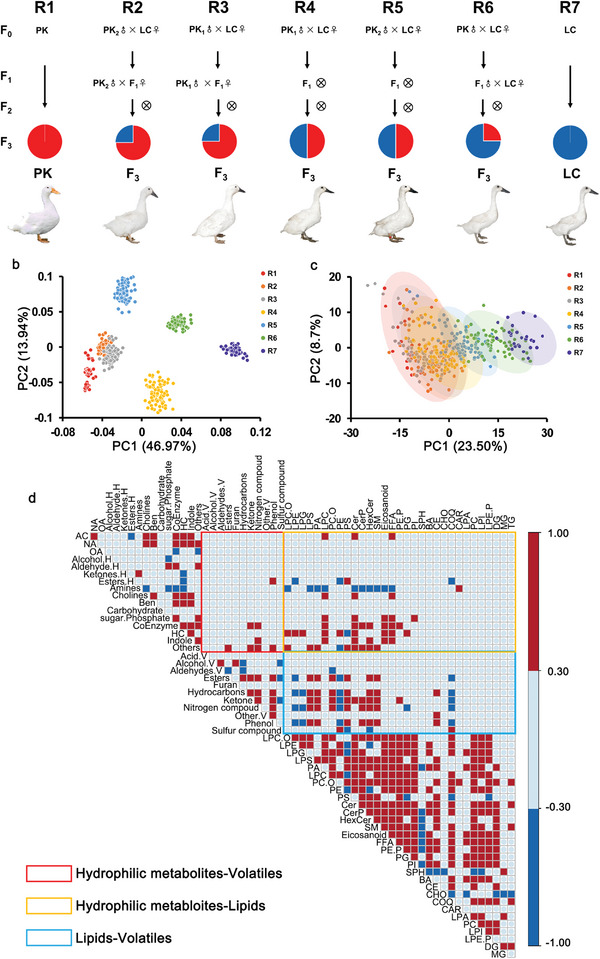
Meat metabolome profile and correlation for the Pekin duck × Liancheng duck gradient consanguinity segregating population. a) Gradient consanguinity design used for the generation of a large segregating duck population for the genetic and biochemical basis of metabolites and volatiles. b) PCA divided 423 individuals into six independent clusters based on SNPs. c) PLS‐DA analysis of 423 individuals shows continuously distributed clusters of the 7 lines based on the levels of metabolites and volatiles. d) A heatmap depicting Pearson's correlation between secondary categories of metabolites and volatiles. The red box represents a Pearson's correlation greater than 0.3, and the blue box represents a Pearson's correlation less than −0.3. Three rectangles are annotated on the bottom left.

We collected 423 individual breast muscle samples from a gradient consanguinity F_3_ segregating population generated by Pekin duck × Liancheng duck crosses at 6 weeks of age (**Figure** **1**a; Table S1, Figure S2, Supporting Information). Based on the broadly targeted profiling method using liquid chromatography–tandem mass spectrometry (LC–MS/MS), we detected 3431 metabolic features in total. Among them, 321 hydrophilic metabolites and 950 lipids were annotated. Amino acids, nucleotides, vitamins, carbohydrates, and their derivatives comprised the main identified hydrophilic compounds. The annotated lipids contained 6 categories, including glycerophospholipids (GPs), glycerolipids (GLs), sphingolipids (SPs), fatty acyls (FAs), sterol lipids (STs), and prenol lipids (PRs) (**Table** **1**; Figure S3a,b, Tables S2,S3, Supporting Information). Furthermore, volatile compounds were produced from the same meat samples and investigated using gas chromatography‐high resolution mass spectrometry combined with the solid‐phase microextraction technique (SPME‐GC‐HRMS). A total of 702 volatile features were detected. The 153 annotated volatiles consisted of 11 classes, including aldehydes, ketones, alcohols, hydrocarbons, acids, furans, phenols, esters, sulfur‐containing compounds, nitrogen‐containing compounds, and others (Table 1; Figure S3c, Table S4, Supporting Information).

**Table 1:** Summary of hydrophilic metabolites, lipids, and volatiles in meat

	Amino acids and derivatives	Nucleotides and derivatives	Organic acid and derivatives	Amines and cholines	Coenzyme and vitamins	Carbohydrates and derivatives
Hydrophilic metabolites 2, 481	105	56	46	18	17	15
	Benzene and derivatives	Esters	Heterocyclic compounds	Others	Unknown	
	14	12	9	29	2160	
Lipids 950	Glycerophospholipids (GPs)	Glycerolipids (GLs)	Sphingolipids (SPs)	Fatty acyls (FAs)	Sterollipids (STs)	Prenollipids (PRs)
	395	365	86	78	23	3
Volatiles702	Ketones	Aldehydes	Hydrocarbons	Sulfur‐containing compound	Esters	Acids
	27	25	22	16	15	9
	Nitrogen‐containing compound	Furans	Alcohols	Phenols	Others	Unknown
	8	9	8	3	11	549

For the broad variations in hydrophilic metabolites, lipids, and volatiles, 38.08%, 54.00%, and 57.26% had coefficients of variation greater than 50%, respectively (Figure S4, Supporting Information). The broad‐sense heritability (H^2^) was greater than 0.5 for 25.43% hydrophilic features, 15.16% lipids, and 14.67% volatile features. A total of 2.54% of the features had heritability over 0.9 (Figure S4, Supporting Information). These results suggested that genetic variations affected the levels of metabolites and volatiles.

According to partial least squares discriminant analysis (PLS‐DA) based on the total feature contents, seven lines from the 423 individuals showed continuously distributed clusters in line with the consanguinity relationship (Figure 1c; Figure S5, Supporting Information), and we found that the levels of 829 metabolites and volatiles (20.06%) showed increasing or decreasing trends from R1 to R7 (Among them, 367 with increasing trends and 462 with decreasing trends, Table S5, Figure S6, Supporting Information). Then the discriminant features between Pekin duck (*n* = 30) and Liancheng duck (*n* = 30) were screened, including 145 hydrophilic metabolites, 144 lipids, and 180 volatiles (|log_2_fold‐change| > 1 and FDR < 0.05, Table S6, Figure S7, Supporting Information). Based on the trend analysis and differential analysis, a total of the overlapped 174 molecules were selected as the potential compounds with nutrition or flavor values for Peking duck and Liancheng duck. (Table S7, Supporting Information).

In addition, the deposition rules of the total metabolic features at seven different developmental stages (1 day, 1–6 weeks) were explored. Most of the metabolites, such as lysophosphatidylethanolamines and triglycerides (TGs), preferentially accumulated in Pekin duck, while organic acids and their derivatives and sphingomyelins (SMs) had higher concentrations in Liancheng duck. Different metabolic patterns could be attributed to the specific breed characteristics between Pekin duck and Liancheng duck samples (Tables S8–S10, Supporting Information).

The relationship between the metabolome in raw meat and the volatilome in cooked meat has not been clearly and systematically studied to date. To address this issue, metabolites in raw meat and volatiles in cooked meat were profiled, and their correlations were investigated. The results showed that GPs were the main metabolites that were highly correlated with volatiles. GPs had both positive (*r* > 0.3, *P* < 0.05) and negative (*r* < −0.3, *P* < 0.05) influences on the volatile levels since GPs could produce volatiles, and also retain volatiles due to the hydrophobic and hydrophilic forces between the GPs and volatiles. In addition, GLs, including TGs and diglycerides (DGs), had limited effects on the volatile levels (Figure 1d, Table S11, Supporting Information). PC(19:0_18:2), PE(18:2_16:0), and PE(19:0_18:2) (*r* = 0.31, 0.30, and 0.29, respectively) were positively correlated with the 3‐octen‐2‐one level, which was generated through the autoxidation of linoleic acid (C18:2) during the heating procedure.^[^ ^26^ ^]^ Likewise, PE(18:1_20:3) was highly correlated with heptanal (*r* = 0.26) since eicosatrienoic acid (C20:3) could undergo autoxidation and thermal degradation^[^ ^27^ ^]^ (Figures S8,S9, Supporting Information). For the hydrophilic metabolites, only a few were correlated with the volatiles. Valylmethionine and glutamylmethionine containing methionine had significant correlations with methional (*r* = 0.41, 0.39) since methional was generated through the Strecker degradation of methionine^[^ ^28^ ^]^ (Figure S8, Supporting Information).

Furthermore, a systematic correlation between metabolites and volatiles was investigated. We found that the levels of hydrophilic metabolites (betaine, *N*‐acetylglycine, *N*‐methylalanine) and lipids (HexCer (d18:1/18:0), HexCer (d18:1/18:1), HexCer (d18:1/20:1)) were significantly higher in Liancheng duck than in Pekin duck, positively correlated (*r* > 0.35, *P* < 0.05) with volatiles such as tetramethyl‐2‐heptanone, ethyl acetate and 2‐pyrrolidone and negatively correlated with undecanone, capric acid, and caprylic acid (*r* < −0.35, *P* < 0.05). Interestingly, hydrophilic metabolites (*N*‐acetyl‐L‐histidine, nicotinic acid‐adenine dinucleotide) and lipids (PC (15:0_16:0), PE (17:1_18:1)) had significantly higher levels in Pekin duck, negatively correlated with tetramethyl‐2‐heptanone, ethyl acetate and 2‐pyrrolidone (*r* < −0.35, *P* < 0.05) and positively correlated with undecanone, capric acid, and caprylic acid (*r* > 0.35, *P* < 0.05) (Figure S10, Supporting Information). The analysis not only identified the differential metabolites and volatiles between Pekin duck and Liancheng duck but also contributed to the study of flavor‐related pathways.

### 2.2 Genetic Basis of Metabolites and Volatiles in Skeletal Muscle

We generated sequencing data for 423 individuals from 7 gradient consanguinity segregating populations generated by Pekin duck × Liancheng duck crosses at 6 weeks with a mean coverage depth of 5×. These data were used to identify variations at the whole‐genome level. A total of 8665026 SNP datasets were generated. Principal component analysis (PCA) results showed that the 423 individuals could be divided into six independent clusters, indicating that their genetic relationship was consistent with the gradient consanguinity segregating populations (Figure 1b).

We then performed mGWAS using the 4133 metabolite and volatile feature phenotypes in 423 ducks based on a mixed linear model (MLM). A Bonferroni correction of *P* = 8.94 × 10^−8^ was employed as the genome‐wide threshold for all trait associations, and a total of 2862 signals corresponding to 1063 loci for 673 metabolites were detected. Among them, 1696 signals corresponded to 427 hydrophilic metabolic features, 126 signals corresponded to 75 lipids, and 1040 signals corresponded to 171 volatile features (Table S12–S15, Supporting Information). A total of 16.28% of the metabolites detected (673 out of 4133) had at least one significant association, with an average of 4.4 associations per metabolite. In general, these signals showed large effects when explaining the variation: up to 29.65% (*N*‐methyl‐L‐glutamate), 59.17% (PC(14:0_22:6)), and 21.13% (2‐pyrrolidone) for hydrophilic metabolites, lipids, and volatiles, respectively, with an average of 9.7% (**Table** **2**; Tables S12–S14, Supporting Information).

**Table 2:** Summary of genome‐wide significant associations identified in mGWAS

Item	Hydrophilic metabolites	Lipids	Volatiles
Number of traits	427	75	171
Number of lead SNPs^a)^	1696	126	1040
Number of loci^b)^	475	67	521
SNPs above 10% of the variation	342	35	391
Maximum explained variation(%)	29.7	59.2	21.1
Explained variation per SNP(%)	9.5	10.3	9.9

^a)^ The SNPs with the lowest P value in a defined region;

^b)^ Adjacent lead SNPs separated by less than 300 kb were considered as a cluster.

**Figure 2:**
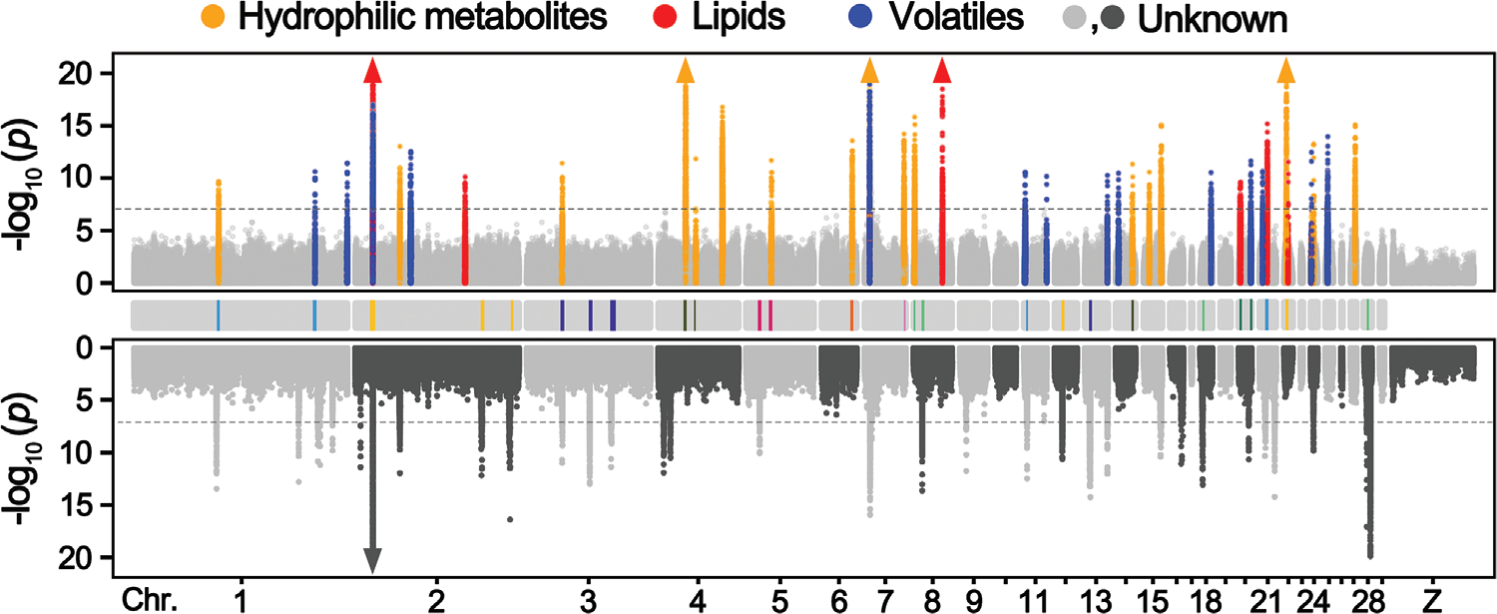
Manhattan plots of mGWAS results with the genetic association. The strength of association for known (top) and unknown (bottom) metabolites and volatiles is indicated as the negative logarithm of the *P* value for the MLM. All metabolite and volatile SNP associations with *P* values below 8.94 × 10^−8^ (horizontal dashed line in all Manhattan plots) are plotted against genome location in intervals of 1 Mbp. The bars in the middle panel represent the overlapped regions for mGWAS signals and selective signals. Triangles represent metabolites and volatile SNP associations with *P* values below 1.0 × 10^−20^.

Manhattan plots of the significant signals that were detected repeatedly are also illustrated, including 51 signals corresponding to amino acids and their derivatives, GPs, TGs, and other known metabolites, as well as 190 signals corresponding to currently unknown metabolites (**Figure** **2**, Table S16, Supporting Information). Genome‐wide analysis of the significant loci identified a significant deviation from a random distribution across the 29 autosomes (*χ* ^2^ = 186.4, *P* < 2.2 × 10^−16^), suggesting that these significant regions contained major genes controlling the levels of large sets of metabolites. A total of 137 potential mGWAS hot spots (signal number > 7, permutation test, *P* < 0.01) were identified and located on chromosomes 2, 7, and 28 (Figure S11a, Table S17, Supporting Information).

To explore whether the genetic divergence between the Pekin duck and Liancheng duck results in changes in metabolite contents, we scanned the genome for regions with extreme divergence in allele frequency (top 1%) in a 10‐kb sliding window on autosomes, showing that 52.14% (85 of 163) of diverse regions overlapped with mGWAS signals (Figure 2; Figure S11b, Table S18, Supporting Information). These results indicated that the levels of breed‐specific metabolites and volatiles often changed alongside the genetic divergence, supporting the opinion that metabolites are regarded as a bridge between the genome and phenome.^[^ ^16^ ^]^

Based on our mGWAS results, we searched for candidate genes utilizing the following principles: i) LD analysis for significant loci, ii) gene expression, and iii) prior knowledge. We were able to identify 48 candidate genes modulating metabolites and volatiles important for muscle physiological or nutritional traits (**Table** **3**; Figure S12, Tables S19,S20, Supporting Information). In summary, we identified a large set of mGWAS signals in the meat metabolome and volatilome whose levels were under complex genetic regulation.

**Table 3:** Summary of 48 genes assigned from mGWAS results

Metabolite	Class	‐LOG10(P)	Region	Candidate gene	Description
Citric acid	Hydrophilic metabolites	10.98	Chr1:41.40‐41.20Mb	*KLF12*	Activator protein‐2 alpha is a developmentally‐regulated transcription factor
Cer(d18:1/20:0)	Lipids	11.56	Chr1:51.95‐52.05Mb	*TGDS*	A member of the short‐chain dehydrogenases/reductases (SDR) superfamily
L‐Cystathionine	Hydrophilic metabolites	9.66	Chr1:80.20‐80.40Mb	*CBSL*	Cystathionine beta‐synthase‐like protein
LPC(O‐20:0)	Lipids	9.22	Chr1:134.15‐135.69Mb	*FAR2*	Fatty Acyl‐CoA Reductase 2
2'‐Deoxycytidine 5'‐diphosphate	Hydrophilic metabolites	9.05	Chr1:156.20‐156.40Mb	*FGD6*	FYVE, RhoGEF And PH Domain Containing 6
Benzofuran,2‐methyl	Volatiles	9.42	Chr1:161.00‐162.50Mb	*SYT1*	Synaptotagmin 1
TG(16:0_16:0_17:0)	Lipids	9.09	Chr1:172.75‐173.10Mb	*PNPLA8*	Patatin Like Phospholipase Domain Containing 8
Methylparaben	Hydrophilic metabolites	9.03	Chr1:190.40‐191.20Mb	*MAGI2*	Membrane Associated Guanylate Kinase, WW, and PDZ Domain Containing 2
Pantetheine	Hydrophilic metabolites	10.25	Chr2:0.20‐0.30Mb	*SMARCD3*	SWI/SNF‐Related Matrix‐Associated Actin‐Dependent Regulator
2‐(4‐Hydroxyphenyl)ethanol	Hydrophilic metabolites	9.87	Chr2:7.50‐7.70Mb	*DPP6*	Dipeptidyl Peptidase Like 6
PC(14:0_22:6)	Lipids	76.78	Chr2:16.80‐16.89Mb	*ACBD5*	Acyl‐CoA Binding Domain Containing 5
Histidine	Hydrophilic metabolites	11.41	Chr2:42.40‐42.50Mb	*GADL1*	Glutamate Decarboxylase Like 1
PC(17:1_20:4)	Lipids	16.10	Chr2:46.00‐46.80Mb	*COL15A1*	Collagen Type XV Alpha 1 Chain
2‐Decanone	Volatiles	9.28	Chr2:70.00‐72.50Mb	*CDH12*	Cadherin 12
PE(24:0_18:1)	Lipids	9.11	Chr2:76.56‐76.63Mb	*CDH6*	Cadherin 6
TG(16:0_16:2_16:3)	Lipids	9.68	Chr2:100.60‐101.20Mb	*CDH7*	Cadherin 7
LPC(24:0/0:0)	Lipids	10.13	Chr2:104.34‐104.53Mb	*MYL2*	Myosin regulatory light chain 2
DG(16:0_19:1)	Lipids	9.56	Chr2:153.60‐153.75Mb	*TSNARE1*	Predicted to be an integral component of the membrane
PC(15:0_16:1)	Lipids	11.01	Chr3:16.90‐17.05Mb	*CSTL1*	Cystatin‐like
Glutathione reduced form	Hydrophilic metabolites	10.17	Chr3:21.29‐21.30Mb	*LIN9*	Lin‐9 DREAM MuvB Core Complex Component
Uridine 5‐monophosphate	Hydrophilic metabolites	10.08	Chr3:33.45‐33.90Mb	*GLO1*	Glyoxalase I
DG(18:0_20:3)	Lipids	9.84	Chr3:33.25‐33.40Mb	*ZFAND3*	Zinc Finger AN1‐Type Containing 3
TG(14:0_14:1_16:1)	Lipids	9.18	Chr3:82.60‐82.70Mb	*TPBG*	Trophoblast Glycoprotein
2‐Methylguanosine	Hydrophilic metabolites	9.54	Chr4:10.20‐10.40Mb	*GRID2*	Glutamate Ionotropic Receptor Delta Type Subunit 2
PA(18:2_18:2)	Lipids	10.14	Chr4:10.90‐11.10Mb	*CCSER1*	Coiled‐Coil Serine Rich Protein 1
*N*‐Methyl‐L‐glutamate	Hydrophilic metabolites	29.97	Chr4:25.70‐26.20Mb	*AADAT*	Aminoadipate Aminotransferase
Nicotinamide Mononucleotide	Hydrophilic metabolites	16.78	Chr4:60.80‐60.90Mb	*BST1*	Bone Marrow Stromal Cell Antigen 1
Punicic Acid	Hydrophilic metabolites	9.91	Chr5:5.00‐5.10Mb	*MDGA2*	MAM Domain Containing Glycosylphosphatidylinositol Anchor 2
4‐Acetamidobutyric acid	Hydrophilic metabolites	11.69	Chr5:23.60‐24.30Mb	*FLRT2*	Fibronectin Leucine Rich Transmembrane Protein 2
6‐O‐methylguanine	Hydrophilic metabolites	8.94	Chr6:25.70‐25.80Mb	*ZCCHC24*	Zinc Finger CCHC‐Type Containing 24
p‐Chlorophenylalanine	Hydrophilic metabolites	10.00	Chr6:28.40‐28.70Mb	*NCOA4*	Nuclear Receptor Coactivator 4
2‐Pyrrolidone	Hydrophilic metabolites	29.16	Chr7:4.71‐4.80Mb	*AOX1*	Aldehyde Oxidase 1
1‐Acetylindole	Hydrophilic metabolites	10.25	Chr7:5.90‐6.00Mb	*ANKRD44*	Ankyrin Repeat Domain 44
*L*‐carnosine	Hydrophilic metabolites	11.99	Chr7:37.40‐37.50Mb	*CARNMT1*	Carnosine *N*‐Methyltransferase 1
(R)‐(‐)‐2‐phenylglycine	Hydrophilic metabolites	15.85	Chr8:1.00‐1.20Mb	*BICC1*	BicC Family RNA Binding Protein 1
3‐Octen‐2‐one	Volatiles	9.39	Chr8:6.25‐6.35Mb	*LEPROT*	Leptin Receptor Overlapping Transcript
Sarcosine	Hydrophilic metabolites	9.16	Chr8:12.00‐12.35Mb	*BEND5*	BEN Domain Containing 5
Cer(d18:0/16:0)	Lipids	14.47	Chr8:27.38‐27.40Mb	*FMO5*	Flavin Containing Dimethylaniline Monoxygenase 5
2,3‐Pentanedione	Volatiles	9.33	Chr11:1.10‐1.20Mb	*PAQR5*	Progestin And AdipoQ Receptor Family Member 5
*L*‐Serine	Hydrophilic metabolites	10.09	Chr14:15.95‐16.05Mb	*LCP2*	Lymphocyte Cytosolic Protein 2
*L*‐Proline	Hydrophilic metabolites	10.58	Chr15:5.50‐5.65Mb	*ELFN1*	Extracellular Leucine Rich Repeat And Fibronectin Type III Domain Containing 1
cis‐Citral	Hydrophilic metabolites	15.08	Chr15:15.90‐17.80Mb	*EMP2*	Epithelial Membrane Protein 2
SM(d18:1/12:0)	Lipids	10.46	Chr20:2.20‐2.24Mb	*FBN3*	Fibrillin 3
PE(P‐18:0_20:3)	Lipids	13.62	Chr21:7.67‐7.76Mb	*ADNP*	Neuroprotective factor
PE(P‐18:1_18:2)	Lipids	15.19	Chr21:7.20‐7.55Mb	*TMEM189*	Plasmanylethanolamine Desaturase 1
4‐Heptanone	Volatiles	9.49	Chr21:10.35‐10.45Mb	*NEK6*	Transferase
Guanidinoethyl sulfonate	Hydrophilic metabolites	29.02	Chr22:2.19‐2.23Mb	*AGMAT*	Agmatinase
TG(18:0_18:1_20:3)	Lipids	9.18	Chr25:0.50‐0.70Mb	*ST3GAL4*	ST3 Beta‐Galactoside Alpha‐2,3‐Sialyltransferase 4

### 2.3 *TMEM189*, a Key Gene that Regulates the Synthesis of Plasmalogens

**Figure 3:**
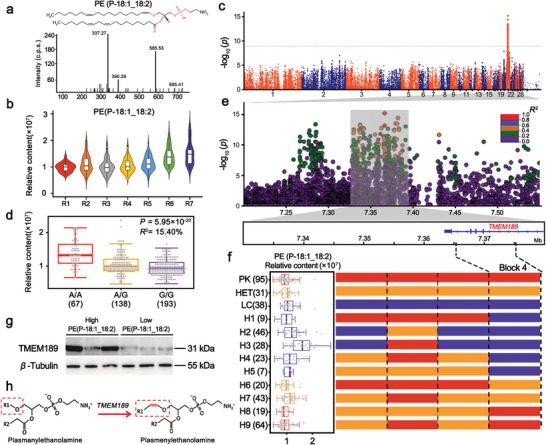
Genetic regulation of plasmalogen levels. a) Structure and MS/MS spectrum of PE(P‐18:1_18:2). b) Violin plot of the levels of PE(P‐18:1_18:2), which showed an increasing trend from R1 to R7. c) Manhattan plots of mGWAS for PE(P‐18:1_18:2). d) Box plot for PE(P‐18:1_18:2) content, plotted as a function of genotypes at SNP Chr21: 7366333 bp. e) Regional plots for the loci ranging from 7.20 Mbp to 7.55 Mbp associated with PE(P‐18:1_18:2) content. All genotyped SNPs are color‐coded according to their pairwise LD values based on the lead SNP. SNPs are colored based on the strength of LD values (*r* ^2^ values) considering the most strongly associated SNP and the other SNPs in the region. f) Recombination event analyses are shown in schematic form in this plot. Red bars refer to chromosomal segments originating from Pekin ducks, purple bars refer to segments originating from Liancheng ducks, and orange bars refer to segments originating from heterozygotes. H1‐9 refer to nine recombinant types. The left box plot refers to PE(P‐18:1_18:2) content. The numbers of individuals are given in brackets. Box plots denote median (centerline), 25–75th percentile (limits), minimum and maximum values without outliers, and outliers (gray dots). g) Western blot analysis showing TMEM189 protein levels between high‐PE(P‐18:1_18:2) content individuals (*n* = 3) and low‐PE(P‐18:1_18:2) content individuals (*n* = 3). The expression level of *β*‐Tubulin was used as a loading control. Western blots show representative data from experiments performed three times. h) *TMEM189* encodes plasma ethanolamine desaturase (PEDS1), which introduces the vinyl ether double bond in PE‐P.

Plasmalogens, which contain a characteristic vinyl ether‐linked alkyl chain at the sn‐1 position of the glycerol backbone,^[^ ^29^ ^]^ have long been reported to maintain the cell membrane and have potential in therapeutic strategies for neurodegenerative and cardiometabolic diseases.^[^ ^30^, ^31^ ^]^ In general, plasmenylethanolamine (PE‐P) accounts for more than half of the total plasmalogens in muscle.^[^ ^32^ ^]^ The levels of PE(P‐18:1_18:2) showed an increasing trend from R1 to R7 (**Figure** **3**a,b). Moreover, the PE(P‐18:1_18:2) level was significantly associated (*P* = 6.4 × 10^−16^) with an SNP (7366333 bp) on chromosome 21 spanning a QTL interval from 7.20 to 7.55 Mbp (Figure 3c). The lead SNP with the highest association with PE (P‐18:1_18:2) content explained 15.40% of the total variance (Figure 3d).

Then, we defined the causal region by calculating pairwise LD within this QTL surrounding the lead SNP (Chr21: 7366333 bp). Seventy SNPs spanning a region from 7.33–7.43 Mbp were highly correlated (pairwise *r* ^2^ > 0.4; Figure 3e). To further narrow the QTL, we characterized the recombination events in the candidate region and identified three recombinant breakpoints that divided the 423 ducks into twelve haplotypes (Figure 3f). Only the haplotypes in block 4 (Chr21:7376251‐7376302 bp) located on the *TMEM189* gene could absolutely distinguish the levels of PE (P‐18:1_18:2) (Figure 3f).

*TMEM189* encodes plasmanylethanolamine desaturase 1 (PEDS1).^[^ ^29^ ^]^ PEDS1 is a key enzyme in the biosynthesis of plasmalogens, introducing the vinyl ether double bond and generating PE‐P^[^ ^33^ ^]^ (Figure 3h). Notably, *TMEM189* was highly and specifically expressed in both adipose tissue and breast muscle (Figure S13a, Supporting Information). Then, we performed a Western blot analysis of TMEM189 in the breast muscles of individuals at 6 weeks with high PE(P‐18:1_18:2) contents and low PE(P‐18:1_18:2) contents (*n* = 3, respectively). A higher protein expression of TMEM189 was observed in individuals with high‐PE(P‐18:1_18:2) contents than those with low‐PE(P‐18:1_18:2) contents, resulting in the differences in PE(P‐18:1_18:2) levels (Figure 3g; Figure S13b, Supporting Information). The gene coding for the plasmalogen biosynthesis enzyme in livestock has not yet been fully identified. Our study supports that genetic variation in *TMEM189* affects the levels of PEDS1 protein expression regulating the amount of PE(P‐18:1_18:2) in muscle, which could possibly be used as a dietary supplement to prevent or treat neurodegenerative diseases.

### 2.4 Identification of a 2‐Pyrrolidone Synthesis‐Controlling Gene

**Figure 4:**
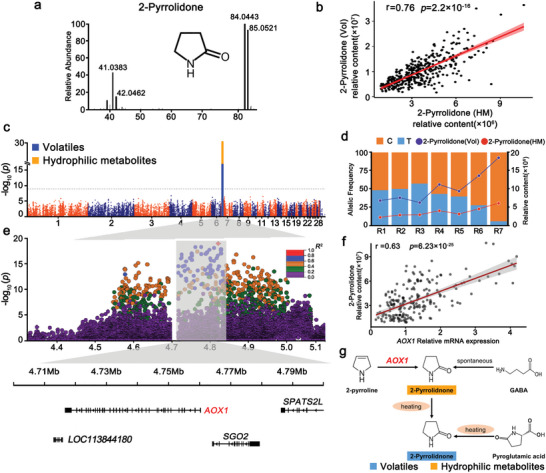
Genetic regulation of the levels of 2‐pyrrolidone a) Structure and EI–MS spectrum of 2‐pyrrolidone. b) The correlation of 2‐pyrrolidone (hydrophilic metabolites) and 2‐pyrrolidone (volatiles). c) Manhattan plots of mGWAS for 2‐pyrrolidone (hydrophilic metabolites, orange dot) and 2‐pyrrolidone (volatiles, blue dot). d) 2‐Pyrrolidone (hydrophilic metabolites) content change corresponding to allele frequencies at a locus on Chr7: 4810144 bp in R1–R7. e) Regional plots for the QTL ranging from 4.30 to 5.10 Mbp associated with 2‐pyrrolidone content. All genotyped SNPs are color‐coded according to their pairwise LD calculated based on the lead SNP. SNPs are colored based on the strength of LD values (*r* ^2^ values) considering the most strongly associated SNP and the other SNPs in the region. f) Relationship between *AOX1* gene expression and 2‐pyrrolidone content in 213 individuals (*r* = 0.63, *P* = 6.23 × 10^−25^). g) The integrated metabolic networks of the 2‐pyrrolidone biosynthesis pathway.

2‐Pyrrolidone was identified as a potential characteristic volatile distinguishing Pekin duck and Liancheng duck. This metabolite gives off a maple and popcorn‐like smell when heated.^[^ ^34^ ^]^ 2‐Pyrrolidone was present in both the hydrophilic metabolome and volatilome, and the correlation coefficient was 0.76 (**Figure** **4**a,b).

According to mGWAS, the levels of 2‐pyrrolidone in the hydrophilic metabolome and volatilome were significantly associated with QTLs (4.56–5.23 Mbp) on chromosome 7 (Figure 4c). The lead SNP (Chr7: 4810144 bp) with the highest association with 2‐pyrrolidone levels explained 28.90% and 21.11% of the total variance (Figure S14a,b, Supporting Information). Interestingly, we found that 2‐pyrrolidone levels showed an increasing trend from R1 to R7 with the increasing T allele frequency at the lead SNP (Chr7: 4810144 bp; Figure 4d). These data indicate that this allele frequency plays an important role in 2‐pyrrolidone content due to the genetic divergence between the Pekin duck and the Liancheng duck. To narrow the candidate region, we examined the lead SNP closely by calculating pairwise LD between the SNPs within the QTL (Chr7:4.56–5.23 Mbp) surrounding the lead SNP. Fifty‐nine SNPs spanning a region from 4.56 to 5.00 Mbp were highly correlated (pairwise *r* ^2^ > 0.6; Figure 4e). Additionally, this QTL contained 3 annotated genes (*AOX1, SGO2*, and *SPATS2L*) (Figure 4e). *AOX1* encodes aldehyde oxidase. It has been reported that the 2‐pyrrolidone content decreases when aldehyde oxidase is deficient.^[^ ^35^, ^36^ ^]^

To further determine the candidate gene, we compared the expression of 3 candidate genes and 2‐pyrrolidone levels in the skeletal muscle of Pekin ducks and Liancheng ducks at seven developmental stages (1 day and 1–6 weeks). Only *AOX1* expression in Liancheng ducks was higher than that in Pekin ducks at 1 and 2 weeks. The 2‐pyrrolidone levels were correspondingly higher at 1 and 2 weeks (Figure S14c, Supporting Information). Then, we used qRT‐PCR to measure the *AOX1* gene expression on 213 F_3_ individuals and examined the correlation with 2‐pyrrolidone content. These results shown that *AOX1* gene expression was significantly correlated with the levels of 2‐pyrrolidone (*r* = 0.63, *P* = 6.23 × 10^−25^), suggesting that the higher expression of *AOX1* in Liancheng ducks led to increased levels of 2‐pyrrolidone (Figure 4f, Table S21, Supporting Information). In addition, 2‐pyrrolidone could be generated through direct volatilization when heated (Figure 4g). These results indicated that 2‐pyrrolidone is highly stable and can be directly used as a molecular marker in metabolome‐assisted breeding for flavor.

### 2.5 Glycerophospholipids and their Autoxidation‐Induced Volatiles are Regulated

**Figure 5:**
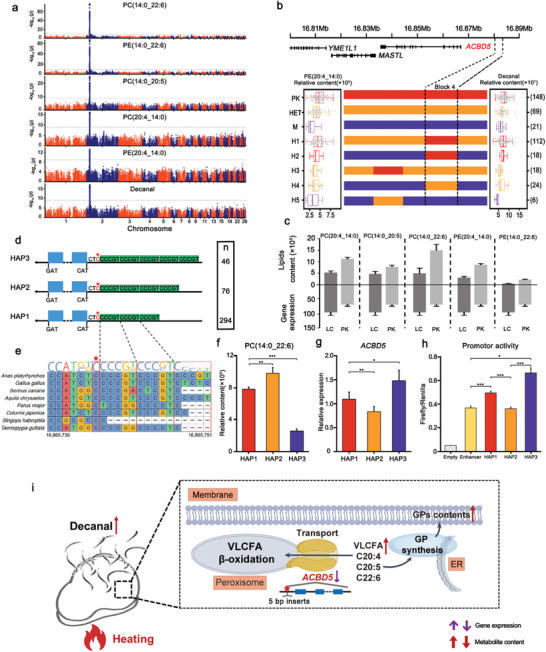
Identification of shared QTL for key genes regulating the levels of 5 GPs and decanal. a) Manhattan plots of mGWASs for 5 GPs and decanal. b) Recombination event analyses are shown in schematic form in this plot. Red bars refer to chromosomal segments originating from Pekin ducks, purple bars refer to segments originating from Liancheng ducks, and orange bars refer to segments originating from heterozygotes. H1‐5 refer to the five recombinant types. The left box plot refers to PE(20:4_14:0) content. The right box plot refers to decanal content. The numbers of individuals are given in brackets. Box plots denote median (centerline), 25–75th percentile (limits), minimum and maximum values without outliers, and outliers (gray dots). c) Five changed GPs (*n* = 5) and the corresponding differentially expressed gene *ACBD5* (*n* = 3). Data represent the mean ± SEM. d) Structural variations in the three *ACBD5* haplotypes. e) Conserved alignment of the sequences surrounding Chr2:16865736 (→C, →CCCCGT, →CCCCGTCCCGT) in birds. f) The relative content of PC(14:0_22:6) in three haplotypes (*n* = 294, 76 and 46, respectively), data represent the mean ± SEM. g) *ACBD5* gene expression of the three haplotypes (*n* = 10, 16 and 15, respectively), data represent the mean ± SEM. h) Promoter activity tests for three haplotypes. This shows that HAP3 (10 bp inserts) had higher promoter activity than HAP2 (5 bp inserts) and HAP1. Data represent the mean ± SEM. * *P* < 0.05, ** *P* < 0.01, *** *P* < 0.001. (i) The hypothesis that *ACBD5* regulates 5 GPs to affect decanal content.

Lipids are important aroma precursors.^[^ ^37^ ^]^ The autoxidation and thermal degradation of lipids are the main pathways for aroma formation in meat and can produce aldehydes, ketones, and other volatile compounds during the heating process.^[^ ^7^ ^]^ The conversion of volatiles from lipids has been reported and discussed previously.^[^ ^38^ ^]^ However, the genetic basis of this process is still largely unknown. Interestingly, we discovered that five GPs containing very‐long‐chain fatty acids (VLCFAs, PC(14:0_22:6), PE(14:0_22:6), PC(14:0_20:5), PC(14:0_20:4), and PE(20:4_14:0)) and volatile decanal were significantly associated and shared QTL hotspots on Chr2:16 Mbp (**Figure** **5**a).

To further narrow the QTL, we examined recombination events in the shared QTL region. Four recombination breakpoints were identified and divided the 423 ducks into 5 haplotypes according to 56 SNPs. Identity‐by‐descent (IBD) analysis indicated that the genotypes in block 4 on Chr2: 16880032‐16881763 bp could distinguish the levels of 5 GPs and decanal. Block 4 was upstream of *ACBD5* and considered the smallest candidate region (Figure 5b). Moreover, this region showed a large genetic divergence between Pekin ducks and Liancheng ducks (Figure S15, Supporting Information). It was reported that *ACBD5* was involved in peroxisomal VLCFA *β*‐oxidation and preferentially bound very‐long‐chain fatty acyl‐CoAs (VLC‐CoAs).^[^ ^39^, ^40^, ^41^ ^]^

Then, we found that *ACBD5* expression in Liancheng ducks was significantly higher than that in Pekin ducks at 5 weeks of age and was accompanied by a lower level of 5 GPs in Liancheng ducks (Figure 5c). To investigate functional variations at the *ACBD5* locus, we found that a tri‐allele short tandem repeats (→C, →CCCCGT, →CCCCGTCCCGT) at position Chr2:16865736 in the *ACBD5* promoter region could be classified into three haplotypes (Figure 5d, Table S22, Supporting Information). We performed a BLAST search of the sequences surrounding Chr2:16865736 (→C, →CCCCGT, →CCCCGTCCCGT) (40 bp upstream and 40 bp downstream), and we found that this locus was conserved among birds (Figure 5e). Interestingly, Hap 3 showed lower contents of the 5 GPs than Hap 1 and Hap 2 (Figure 5f). Moreover, we also found lines with low GP contents had increased *ACBD5* expression and contained 10 bp inserts while lines with high GP contents contained 5 bp inserts or no inserts (Figure 5g, Table S23, Supporting Information).

To further determine whether variation in the promoter affected gene expression, we compared the transcriptional activity of the promoters of HAP1, HAP2, and HAP3 using a dual luciferase reporter gene assay. Hap3_pro exhibited 1.85‐fold higher activity than Hap2_pro, resulting in the observed higher expression of Hap3 than Hap2. This was consistent with the observed differences in gene expression of *ACBD5* among three haplotypes (Figure 5h).

These results indicated that *ACBD5* was a candidate gene regulating the levels of these 5 GPs by transporting VLC‐CoAs into the peroxisome for *β*‐oxidation. Afterward, these VLCFAs were subsequently incorporated into the membrane.^[^ ^41^, ^42^ ^]^ Lipids could be oxidized to volatiles such as decanal through peroxidation and degradation during heating (Figure 5i). This is a typical example of the genetic regulation of lipids and its further influence on the volatiles in meat.

### 2.6 Identification of Synthesis‐Controlling Genes for Carnosine and Related Metabolites

**Figure 6:**
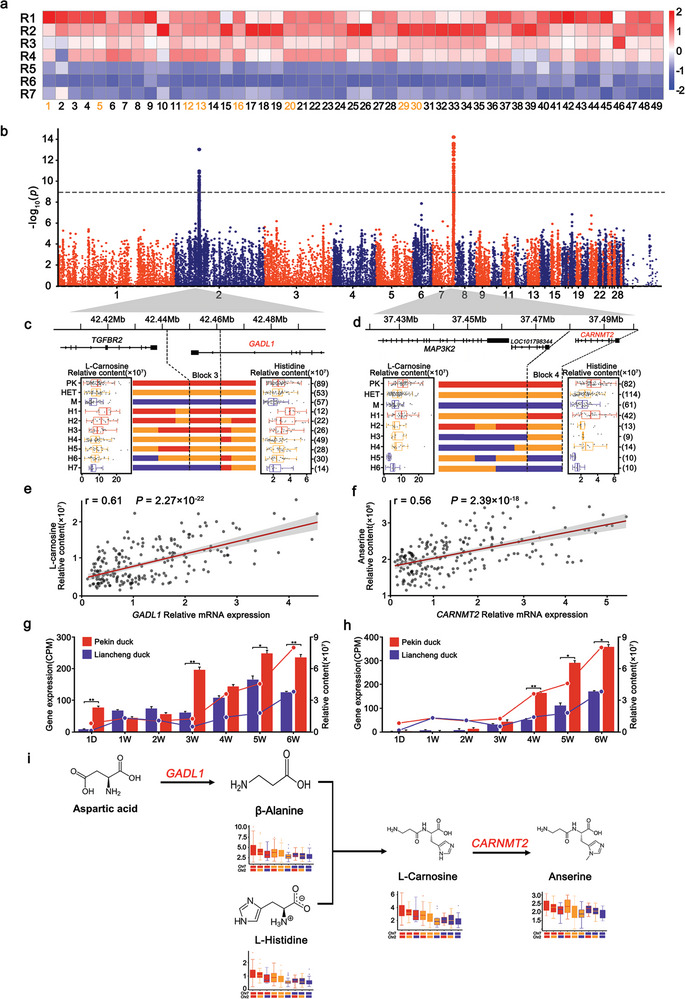
The genetic regulation of the levels of carnosine and related metabolites and metabolic pathways. a) Heatmap of the levels of carnosine and 49 related metabolites detected in this study that showed a decreasing trend from R1 to R7. b) Manhattan plots of mGWAS for carnosine and 49 related metabolites. Recombination event analyses on Chr2 (c) and Chr7 (d) are shown in schematic form in this plot. Red bars refer to chromosomal segments originating from Pekin ducks, purple bars refer to segments originating from Liancheng ducks, and orange bars refer to segments originating from heterozygotes. H1‐7 refers to the seven recombinant types on Chr2, and H1‐6 refers to the six recombinant types on Chr7. The left box plot refers to the L‐carnosine content. The right box plot refers to other metabolite contents. The numbers of individuals are given in brackets. Box plots denote the median (centerline), 25–75th percentile (limits), minimum and maximum values without outliers, and outliers (gray dots). e) Relationship between *GADL1* gene expression and L‐carnosine content in 206 individuals (*r* = 0.61, *P* = 2.27 × 10^−22^). f) Relationship between *CARNMT2* gene expression and anserine content in 208 individuals (*r* = 0.56, *P* = 2.39 × 10^−18^). g,h) L‐Carnosine content changes corresponding to differentially expressed *GADL1* and *CARNMT2*. Data represent the mean ± SEM, as analyzed by Student's *t*‐test, * *P* < 0.05, ** *P* < 0.01, *** *P* < 0.001. i) The integrated metabolic networks of carnosine and related metabolite metabolism pathways.

Carnosine, an animal‐specific metabolite, is considered to be an important antioxidant, pH buffer, and neuromodulator.^[^ ^43^ ^]^ However, its biosynthetic route is controversial. mGWAS identified two significant signals (Chr2, 7) for 49 metabolites, including 8 annotated metabolites, such as carnosine, anserine, and histidine carnosine, as well as 31 unknown metabolites (**Figure** **6**a,b). Moreover, we found that the levels of 49 metabolites showed a decreasing trend from R1 to R7 (Figure 6a, Table S24, Supporting Information).

Using IBD analysis, we discovered the smallest candidate regions, including block 3 (Chr2: 42242000 bp–42261000 bp) located on the *GADL1* gene and block 4 (Chr7: 37472000 bp–37497000 bp) located on the *CARNMT2* gene (Figure 6c,d), which could affect each correlated metabolite level. These two blocks showed a large genetic divergence between Pekin ducks and Liancheng ducks (Figure S16, Supporting Information). Additionally, by evaluating the interactions between the two smallest candidate regions, the effect of the PK haplotypes at block 3 on increasing the amounts of carnosine and related metabolites was found to be primarily dependent on the PK haplotypes at block 4, suggesting the joint genetic control of carnosine and related metabolite contents and that these two blocks may act sequentially in the biosynthesis of carnosine and related metabolites (Figure 6i). *GADL1* has been reported to be involved in *β*‐alanine and carnosine production in mammalian tissues.^[^ ^44^ ^]^ *CARNMT2* encodes carnosine *N*‐methyltransferase 2 and has been reported to convert carnosine to anserine.^[^ ^45^ ^]^ We further used qRT‐PCR to measure the *GADL1* and *CARNMT2* gene expression on 206 and 208 F_3_ individuals, respectively, and then examined the correlation between the mRNA expression of *GADL1*and *CARNMT2* and the levels of carnosine and related metabolites. *GADL1* expression was significantly correlated with the L‐carnosine level (*r* = 0.61, *P* = 2.27 × 10^−22^) while *CARNMT2* expression was significantly correlated with the anserine levels (*r* = 0.56, *P* = 2.39 × 10^−18^) (Figure 6e,f, Tables S25,S26, Supporting Information). In addition, based on the investigation of gene expression and the levels of carnosine and related metabolites in breast muscles at seven stages (1 day, 1–6 weeks), the expression levels of *GADL1* and *CARNMT2* were significantly higher in Pekin ducks and further led to higher levels of carnosine and related metabolites (Figure 6g,h). These results suggested that both *GADL1* and *CARNMT2* are responsible for the accumulation of carnosine and related metabolites.

mGWAS can facilitate identifying and annotating metabolites detected by theoretically linking the metabolites to functionally related genes.^[^ ^19^, ^46^ ^]^ In our study, we examined the candidate regions for *GADL1* and *CARNMT2* and determined whether they could possibly enable the annotation of additional unknown metabolites. We plotted the 49 metabolite traits through their chromatographic retention time and corresponding precursor m/z (Figure S17, Table S27, Supporting Information). These unannotated metabolites shared a retention time region with histidine and its derivatives, suggesting that those unidentified metabolites could possibly be amino acids and their analogs with similar polarities. Additionally, the same QTLs for those unknown metabolites were also located on the *GADL1* and *CARNMT2* genes. The shared genetic regulation further allowed us to predict that these unknown metabolites are histidine and its derivatives. Seven additional metabolites were annotated and verified by high‐resolution mass spectrometry (Figure S18, Supporting Information). All the above data indicated that a class of metabolic pathways was controlled by a few large‐effect loci in the skeletal muscle metabolome.

## 3 Discussion

Meat quality is a broad and complex term that covers various attributes such as texture, hygiene, nutrition, and flavor.^[^ ^47^, ^48^ ^]^ Varieties of metabolites perform indispensable and prominent roles in maintaining and promoting meat quality.^[^ ^49^, ^50^ ^]^ In this study, it was found that 568 metabolites and volatiles were correlated with 7 meat quality traits (water loss, lightness (L*), redness (a*), yellowness (b*), shear force, pH_24h_, and crude fat; |*r*| > 0.3, *P* < 0.05) (Figures S19–S21, Supporting Information). These metabolites could provide both macronutrients and micronutrients, function as flavor precursors and determine unique meat flavors, and facilitate meat processing through complex chemical reactions.^[^ ^51^ ^]^ Currently, rapidly developing metabolomics techniques enable us to determine this complex meat quality issue. Metabolomics approaches make it possible to decompose complex meat quality phenotypes into specific metabolic traits with substructure and chemical class information, broadening and deepening our understanding of meat and meat quality. Currently, meat metabolic research has always placed much emphasis on identifying the different metabolites and volatiles between different varieties.^[^ ^52^, ^53^, ^54^ ^]^ In this study, we identified 174 important metabolites and volatiles in 7 gradient consanguinity segregating populations generated by Pekin duck × Liancheng duck crosses at 6 weeks (Table S7, Supporting Information). Those metabolites and volatiles mostly determine the nutrition and flavor of duck meat. However, the genetic and biochemical bases underlying the metabolites and volatiles were not explored. In this study, we provide the first mGWAS results to understand the genetic and biochemical basis of metabolites and volatiles in duck meat.

mGWAS is a powerful tool for performing association analysis between a large set of metabolites and genetic variants to reveal the genetic basis of metabolic traits.^[^ ^15^ ^]^ In general, genetic variations in primary metabolites tend to be controlled by a large number of small‐effect loci.^[^ ^55^, ^56^, ^57^ ^]^ To better detect small‐effect genetic loci modulating metabolite content, we specifically designed a two‐breed gradient segregation population to improve the power and accuracy in mapping small‐effect and closely linked QTLs. Finally, we detected 1063 significant loci regulating metabolite and volatile contents, which greatly enhanced our knowledge of the genetic basis of the meat metabolome. Moreover, we found that 52.14% of extreme genetic divergence regions between the Pekin duck and Liancheng duck overlapped with mGWAS signals, and these data could provide in‐depth insight into the dissection of complex traits in animals. Integrating these results, the build‐up of a meat metabolome database could facilitate in‐depth research on meat.

Throughout the history of animal breeding, phenotype‐targeted selection has been the common method. High throughput molecular breeding has huge potential to accelerate meat quality improvement. In this study, we identified 48 candidate genes modulating metabolites and volatiles, important for muscle physiological or nutritional traits. Those results would be of great importance for potential marker‐assistant breeding to improve duck meat quality. However, some issues still need to be investigated: 1) the accurate quantitation of these interested metabolites and volatiles in duck meat. 2) The relationship between the genetic regulation of metabolites and volatiles with the growth rates of ducks. 3) How do these metabolites and volatiles affect human sensory evaluation.

## 4 Conclusion

In summary, our results presented a comprehensive metabolomics analysis of meat and enhanced our understanding of the genetic basis of muscle metabolic traits. A valuable roadmap has been created to associate metabolites with genetic variations. We identified abundant essential genes underlying metabolites, increased the knowledge of the nutritional components in muscle, and developed animal meat quality breeding strategies.

## 5 Experimental Section

### Animal and Sample Collection

The study focused on a large gradient consanguinity segregating population generated by Pekin duck × Liancheng duck crosses containing a total of seven lines (R1, R2, R3, R4, R5, R6, and R7) as follows. R1 is a Pekin duck purebred line. R7 is a Liancheng duck purebred line. In the R2 and R3 lines, the F1 generation was produced from PK1♂ (patriline of Pekin duck), PK2♂ (matriline of Pekin duck), and Liancheng duck♀. The F2 generation was produced from PK1♂, PK2♂ and F1 generation♀. The F3 generation was obtained by natural mating of the F2 generation, which was expected to show ≈75% of the genome obtained from the Pekin duck and ≈25% of the genome obtained from the Liancheng duck. In the R4 and R5 lines, the F1 generation was produced from PK1♂ (patriline of Pekin duck), PK2♂ (matriline of Pekin duck), and Liancheng duck♀. The F2 generation was obtained by natural mating of the F1 generation, and the F3 generation was obtained by natural mating of the F2 generation, which was expected to show ≈50% of the genome obtained from Pekin duck and ≈50% of the genome obtained from Liancheng duck. In the R6 line, the F1 generation was produced from PK♂ and Liancheng duck♀. The F2 generation was produced from the F1 generation♀ and Liancheng duck♀. The F3 generation was obtained by natural mating of the F2 generation, which was expected to show ≈25% of the genome obtained from the Pekin duck and ≈75% of the genome obtained from the Liancheng duck (Figure S2, Supporting Information).

A total of 423 gradient consanguinity segregating population ducks (30, 74, 75, 75, 75, 64, and 30 ducks in R1, R2, R3, R4, R5, R6, and R7), along with 35 Pekin ducks and 35 Liancheng ducks, were used in this study (Table S1, Supporting Information). All eggs were incubated using a normal procedure, and all ducks were reared in cages under continuous lighting using standard conditions of temperature, humidity, and ventilation. All ducks were fed the same corn‐ and soybean meal‐based diet, which met the nutrition recommendations of the National Research Council (NRC, 1994). Feed and water were provided ad libitum during the experiment (Table S28, Supporting Information).

For all of the ducks, blood was obtained from the wing vein and was rapidly frozen at −20 °C. Following a 12‐h overnight fast, a slaughter experiment was performed on 423 ducks 42 days after birth. The breast muscle was collected from the left side (1 cm from the upper and left edges of the breast muscle) and immediately snap‐frozen using liquid nitrogen (Figure S2, Supporting Information).

In RNA‐seq, hydrophilic metabolites and lipids samples of breast muscle at seven developmental stages, another 10 ducks (5 Pekin ducks and 5 Liancheng ducks) were randomly selected and slaughtered at days 1, 7, 14, 21, 28, 35, and 42 after birth. The breast muscle was collected from the left side (1 cm from the upper and left edges of the breast muscle) and immediately snap‐frozen using liquid nitrogen (Figure S2, Supporting Information).

All experiments with ducks were performed under the guidance of ethical regulation from the Institute of Animal Science, Chinese Academy of Agricultural Sciences (NO. IAS‐2022‐114), Beijing, China.

### Hydrophilic Metabolite Profiling

The lyophilized muscle samples were crushed using a mixer mill (MM 400, Retsch) with zirconia beads for 1 min at 30 Hz. The crushed sample (80 ± 2 mg) was accurately weighed. Samples were extracted overnight at 4 °C with 1 mL of 70% aqueous methanol and centrifuged at 12 000 rpm for 10 min at 4 °C. The extracts were cleaned up using a CNWBOND Carbon‐GCB SPE cartridge (250 mg, 3 mL; ANPEL, Shanghai, China) and filtered (SCAA‐104, 0.22 µm pore size; ANPEL, Shanghai, China) before analysis.

Hydrophilic metabolites were analyzed using an LC‒ESI‒MS/MS system (HPLC, Shim‐pack UFLC Shimadzu CBM30A system, MS, Applied Biosystems 6500 QTRAP) equipped with an ESI Turbo Ionspray interface controlled by Analyst 1.6.3 software (ABSciex).

The LC analysis conditions were as follows: column, Waters ACQUITY UPLC HSS T3 C18 (1.8 µm, 2.1 mm × 100 mm); mobile phase, A: water (0.04% acetic acid), B: acetonitrile (0.04% acetic acid); gradient program, 5% B at 0 min, 95% B at 11.0 min, 95% B at 12.0 min, 5% B at 12.1 min, 5% B at 15.0 min; flow rate, 0.35 mL min^−1^; column temperature, 40 °C; and injection volume, 2 µL.

The MS parameters were set as follows: source temperature, 550 °C; negative ion spray voltage (IS), (+) 5500 V and (−) 4500 V; gas I (GSI), gas II (GSII), and curtain gas (CUR) were set at 55, 60, and 35 psi, respectively; and the collision gas (CAD) was medium. Instrument tuning and mass calibration were performed with 10 and 100 µmol L^−1^ polypropylene glycol solutions in QQQ and LIT modes, respectively. The QQQ scans were acquired as multiple reaction monitoring (MRM) experiments with the collision gas (nitrogen) set to 5 psi. The declustering potential (DP) and collision energy (CE) for individual MRM transitions were determined with further DP and CE optimization. A specific set of MRM transitions was monitored for each period according to the metabolites that were eluted within this period.

Using this method, a hydrophilic metabolite library was constructed by measuring a total of 68 representative samples (7, 11, 12, 12, 8, 12, and 6 ducks in R1, R2, R3, R4, R5, R6, and R7). Two thousand four hundred and eighty‐one hydrophilic metabolite features that were found to be stable after performing quality control were detected.

### Lipid Profiling

After the sample was thawed, 20 ± 1 mg of powder from each sample was weighed and extracted overnight at 4°C. Then, 1 mL of lipid extraction solution (methyl tert‐butyl ether: methanol = 3:1) was added to the homogenized centrifuge tube to extract the lipids. The steel ball was removed, and the mixture was swirled for 2 min. Then, 200 µL of water was added, and the mixture was swirled for 15 min and centrifuged at 12000 rpm at 4°C for 10 min. Next, 300 µL of supernatant was pipetted, concentrated, and stored at ‐80°C. Before LC‒MS/MS analysis, the dried supernatant was dissolved in 200 µL of mobile phase B (acetonitrile/isopropanol, 10/90, 0.1% acetic acid, and 10 mmol/L ammonium formate) and then stored at ‐80°C. The analysis was performed using an LC‒ESI‒MS/MS system (HPLC, Shim‐pack UFLC Shimadzu CBM30A system, MS, Applied Biosystems 6500 QTRAP) equipped with an ESI Turbo Ionspray interface controlled by Analyst 1.6.3 software (ABSciex).

The LC analysis conditions were as follows: column, Thermo Accucore C30 (2.6 µm, 2.1 mm×100 mm); mobile phase, A: acetonitrile/water (60/40, V/V, 0.1% formic acid, 10 mmol L^−1^ ammonium formate), B: acetonitrile/isopropanol (10/90, V/V, 0.1% formic acid, 10 mmol L^−1^ ammonium formate); gradient program, 20% B at 0 min, 30% B at 2.0 min, 60% B at 4 min, 85% B at 9 min, 90% B at 14 min, 95% B at 15.5 min, 95% B at 17.3 min, 20% B at 17.5 min, 20% B at 20 min; flow rate, 0.35 ml min^−1^; temperature, 45°C; and injection volume, 2 µL.

The ESI source operation parameters were as follows: an ion source, turbo spray; source temperature 500°C; ion spray voltage (IS) (+) 5500 V and (‐) 4500 V; ion source gas I (GSI), gas II (GSII), and CUR were set at 45, 55, and 35 psi, respectively; and the CAD was medium. Instrument tuning and mass calibration were performed with 10 and 100 µmol L^−1^ polypropylene glycol solutions in QQQ and LIT modes, respectively. QQQ scans were acquired as MRM experiments with the collision gas (nitrogen) set to 5 psi. DP and CE for individual MRM transitions were performed with further DP and CE optimization. A specific set of MRM transitions was monitored for each period according to the metabolites eluted within this period.

Using this method, a lipid library was constructed by measuring a total of 81 representative samples (7, 11, 12, 12, 8, 12, and 6 ducks in R1, R2, R3, R4, R5, R6, and R7). Nine hundred and fifty lipid features that were found to be stable after performing quality control were detected.

### Volatile Profiling

The HS‐SPME procedure was the selected extraction mode. To ensure faster extraction, the vial was agitated during the extraction period. SPME was directly performed in a TriPlus RSH autosampler (Thermo Fisher Scientific, Bremen, Germany).

Samples were prepared as follows: Prior to the assay, the muscles were thawed in a refrigerator at 4 °C for 12 h, vacuum packed in a plastic steaming bag and cooked in a thermostated water bath at 80 °C for 30 min. Once the cooking process was finished, the packaged meat was removed from the water bath and submerged in ice‐cold water for 1 h. Once removed from the package, the cooked meat was ground to powder in liquid nitrogen to retain the highest amount of volatiles. A minced sample (3 g) was introduced into a 20 mL glass vial. Then, the internal standard (IS), 10 µL of 2‐methyl‐3‐heptanone solution (0.05 µg µL^−1^), was added. The vials were immediately closed with a magnetic cap fitted with a PTFE‐silicone septum. The sample vial was incubated at 55 °C for 20 min and extracted at 55 °C for 40 min using a 50/30 µm DVB/CAR/PDMS fiber (Supelco, Inc., Bellefonte, PA, USA). Once done, the extraction fiber was automatically injected into the injector and desorbed at 250 °C for 3 min. Between the consecutive analyses, the fiber was conditioned in the other injector port at 270 °C for 10 min.

Analyses were carried out on a Q‐Exactive Orbitrap mass analyzer equipped with a TriPlus RSH autosampler and Trace 1310 GC (Thermo Fisher Scientific, Bremen, Germany).

The chromatographic conditions were set as follows: a 60 m × 0.25 mm i.d. × 0.25 µm film thickness, VF‐WAX ms (Agilent, Santa Clara, CA) column was used. Helium (99.9999%) with a constant flow rate of 1 mL min^−1^ was used as the carrier gas. The injector temperature was set at 250 °C. The split ratio was set as 5:1. The oven temperature was initially kept at 40 °C for 2.0 min, increased to 230 °C at a rate of 4 °C min^−1^, and held for 5 min. Both transfer line 1 and transfer line 2 were set at 250 °C.

MS was performed using electron impact ionization (EI) at 70 eV, operating in full scan mode at a resolving power of 60 000 full width at half maximum. The scan range was from 30 to 400 m z^−1^ with an automatic‐gain‐control target value of 1E6. The ion source and transfer line temperatures were set at 280 and 250 °C, respectively. TraceFinder 4.1 software was used to analyze the data (Thermo Fisher Scientific, Les Ulis, France). During testing, a blank sample and a QC sample were added every 14 samples to ensure the instrument's stability. Volatiles were identified in accordance with mass spectra and linear retention indices from NIST17 (v2.3), Wiley9, and a domestic library built with authentic reference standards. For identification purposes, the HRF scores should be higher than 95; the match factor based on the MS pattern should be higher than 750; and the difference in the retention index should be less than 20 for the domestic library and within 50 for the NIST library.

### Variant Discovery and Genotyping

Genomic DNA was obtained from whole blood samples collected from the brachial veins of ducks and isolated using the standard phenol/chloroform extraction method. The quantity and quality of genomic DNA were assessed by Nanodrop and agarose gel electrophoresis. After the examinations, eligible DNA samples from the 423 individuals were generated into paired‐end libraries using standard procedures. In addition, the average insert size was 500 bp, and the read length was 150 bp. All libraries were sequenced on an Illumina HiSeq X‐Ten platform to an average raw read sequence coverage of 5×. The 150‐bp paired‐end raw reads were mapped to the duck reference genome (GCA_0 038 50225.1) with Burrows–Wheeler alignment (BWA aln)^[^ ^58^ ^]^ using the default parameters. The paired reads that were mapped to the exact same position on the reference genome were identified with MarkDuplicates in Picard^[^ ^59^ ^]^ to avoid any influence on variant detection. After mapping, SNP calling was performed exclusively using the GATK^[^ ^60^ ^]^ HaplotypeCaller module (version 3.5), and the output was further filtered using VCFtools^[^ ^61^ ^]^ (version 0.1.15). SNPs were filtered based on the following criteria: 1) 3 × <mean sequencing depth (overall included individuals) <30 ×, 2) SNPs had to have a minor allele frequency > 0.05, 3) the maximum missing rate was < 0.1, and 4) SNPs had only two alleles. The identified SNPs were further classified by SnpEff^[^ ^62^ ^]^ based on the gene annotation of the reference genome. A total of 423 ducks were genotyped, and 8665026 SNPs were prepared for subsequent analysis.

### Principal Component Analysis

PCA was performed based on all SNPs using EIGENSOFT software (version 4.2).^[^ ^63^, ^64^ ^]^ The gradient consanguinity segregating population was clearly separated into six groups by the first principal component. PLS‐DA was performed based on the levels of 3431 metabolites and 702 volatiles using SIMCA (version 14.1).

### Heritability Estimation and Coefficient of Variation

The broad‐sense heritability (H^2^) of each metabolite and volatile was estimated using the following formula, H^2^ = var(G)/var(G) + var(E), implemented in GCTA^[^ ^65^ ^]^ v1.26.0, where var(G) and var(E) are the variances derived from genetic and environmental effects. The coefficient of variation values were calculated for each metabolite as follows: s/m, where s and m are the standard deviation and mean of each metabolite, respectively.

### Genome‐Wide Association Analysis and Linkage Disequilibrium

A total of 8665026 SNPs for 423 ducks were used to perform the genome‐wide association analysis. The relative content of metabolites and volatile compounds was log_2_ calculated as phenotypic values. Population structure and cryptic relationships were considered to minimize false positives and increase statistical power. The mixed linear model program EMMAX^[^ ^66^ ^]^ was used for the association analysis. The first three PCA values (eigenvectors) derived from whole genome SNPs, as well as sex, were set as fixed effects in the mixed model to correct for population stratification. The random effect was a kinship matrix estimated based on the identity‐by‐state algorithm among all individual whole‐genome SNPs. The whole‐genome significance cutoff as the Bonferroni test threshold was defined, and the association threshold was set as 0.01/total SNPs (total SNPs: 8665026; −log_10_ (*P*) = 8.938). Linkage disequilibrium (LD) analyses were performed based on the *R* ^2^ value between the SNPs within 2 Mbp of the lead SNP (MAF > 0.05) within the region using PLINK^[^ ^67^ ^]^ (version 1.90).

### Recombinant Event Analysis

The fine‐mapped analyses were performed using IBD analysis. To conduct this analysis, the filtered SNPs reached a standard allele frequency difference (ΔAF) of >0.6 between Pekin ducks and Liancheng ducks, according to previous genotyping results. In the candidate region, recombination breakpoints were identified across the filtered SNPs, and the individuals in each population were subsequently classified by using the recombination breakpoints.

### mGWAS Hotspot Analysis

The whole genome was divided into 1 Mbp partitions to investigate the distribution of significant signals along the genome. Then, the number of significant signals in each segment were counted. A permutation test was used to assess the statistical significance of the deviation compared to the observed significant signal distribution per segment from the expectation assuming a uniform distribution.

All the signals were randomly assigned to each 1 Mbp segment of the genome, and the resulting number of significant signals in each segment was counted. After 10 000 permutations, with *P* < 0.01, the cutoff for significant signals in each 1 Mbp segment by chance alone was 7, and a large number of regions were regarded as hot spots.

### Genome Scanning for Divergent Regions

To detect the regions with genetic divergence, the *F*_ST_ value among 30 Pekin ducks and 30 Liancheng ducks were calculated using a 20 kb window with a 10 kb step across the whole genome using Vcftools^[^ ^61^ ^]^ (version 0.1.15). Windows with the top 1% were selected as the candidate genetic divergence region. The candidate region was detected by searching the regions with high *F*_ST_ values and high differences in genetic diversity (*π* ln ratio). First, among 30 Pekin ducks and 30 Liancheng ducks, the *F*_ST_ values for each SNP site and *π* ln ratio in sliding 5 kb windows were calculated with a 2.5 kb step using vcftools (version 0.1.15).

### Transcriptome Analysis

The breast muscle tissue was lyophilized in liquid nitrogen, dissolved in TRIzol reagent, and prepared for subsequent library construction. All extracted RNA quality and quantity were assessed by Nanodrop and agarose gel electrophoresis. RNA samples were reverse transcribed to cDNA with PrimerScript RT Master Mix (RR036A, Takara, Dalian, China) following the manufacturer's instructions. Forty‐two library preparations were sequenced on an Illumina X‐Ten platform, and 150‐bp single‐end reads were generated. The average output was 6 Gb per library. Sequencing adaptors and low‐complexity reads were removed by Trimmomatic^[^ ^68^ ^]^ version 0.36 software in the initial data filtering step. Then, clean data were mapped to the duck reference genome (GCA_0 038 50225.1) using TopHat^[^ ^69^ ^]^ version 2.0.11 software. Read counts per gene were obtained by running HTSeq^[^ ^70^ ^]^ version 0.6.1 software. The counts per million mapped sequence read (CPM) for each gene were calculated by edgeR^[^ ^71^ ^]^ version 3.20.9 packages.

### Quantitative PCR Analysis

qPCR was conducted for *AOX1*, *GADL1*, *CARNMT2* and 3 haplotypes of the *ACBD5* gene. Primers were designed with Primer 5 software. The primer sequences were listed in Table S29 (Supporting Information). Breast muscle tissues from gradient consanguinity segregating population were collected. Complementary DNA synthesis from total RNA and two‐step quantitative PCR were performed using the Applied Biosystems QuantStudio system. All samples were assayed in at least three technical replicates. The collected data were analyzed using the 2^−ΔΔCt^ method,^[^ ^72^ ^]^ and all the results were normalized to the duck *β*‐*actin* gene.

### Western Blot Analysis for TMEM189

Frozen breast muscle (≈30 mg) was weighed and minced the samples in liquid nitrogen. Afterward, 300 µL RIPA‐proteinase K inhibitor (Beijing Solarbio Science & Technology, Beijing, China) was added for protein extraction. The BCA protein quantification kits (Thermo Fisher Scientific, Waltham, MA) were used for the quantitation of proteins in breast muscles. Protein samples (33 µg) were separated on 4–20% Bis‐Tris SurePAGETM gels (Genscript, Nanjing, China) and electro‐transferred to PVDF membranes (Pall, Pensacola, FL, America). Then, the membranes were blocked with 5% nonfat milk (BD Difco, Sparks, MD, USA) for 2 h at room temperature and incubated overnight at 4 °C with primary antibodies against TMEM189 (1:1000, ABclonal, Wuhan, China) and *β* ‐Tubulin (1:5000, Huaxingbio, Beijing, China). After washing with 1 × TBST three times, the membranes were incubated at room temperature for 1 h with appropriate secondary antibodies (1:5000 dilution, goat anti‐rabbit, Solarbio). Subsequently, the blot bands were visualized with an ECL reagent (Beijing Lan Y Science & Technology). The optical densities of the blot bands were analyzed using Tanon Gis 1D software (Tanon Sciences & Technology, Beijing, China). Finally, the protein expression was normalized by *β*‐tubulin and calibrated with the CON value.

### Luciferase Reporter Assay

Three haplotypes of the *ACBD5* gene (HAP1, HAP2. HAP3) and their 50 bp upstream and 50 bp downstream were generated via PCR and cloned into the pGL3 enhancer vector; these fragments included HAP1 (100 bp), HAP2 (105 bp), HAP3 (110 bp). The *Xhol* and *HindIII* enzyme sites were selected as the insertion sites of PCR products. Duck embryo fibroblasts cell were plated at a density of 1 × 10^5^ per well in 48‐well plates 1 day before transfection and were cultured under adherent conditions in high‐glucose DMEM (HyClone) +10% FBS (fetal bovine serum, Gibco). Cells were transfected with 220 ng (per well) of serial plasmids containing different segments of the three haplotypes sequence and 20 ng (per well) of the pRL‐ TK Renilla Luciferase plasmid using Lipofectamine 8000 (Invitrogen). Luciferase activities were determined by using the Dual‐Luciferase Reporter Assay System (Promega), according to the manufacturer's instructions. Luciferase bioluminescence measurements were performed with a Veritas Microplate Luminometer (Promega). All of the experiments were conducted in triplicate, and the firefly luciferase activity was normalized to the Renilla luciferase activity of each sample.

### Statistical Analysis

Data were presented as the mean ± standard error of the mean. Statistical analyses were performed in R 3.6.3. Pearson correlation analysis was used to determine the pairwise correlations between metabolites and volatile compounds. For gene expression, the significance of the values among the groups was compared by using Student's *t*‐test. The *P* values from the tests mentioned above were adjusted using FDR correction (BH method) for multiple testing. The filtering criteria for statistical significance were *P* < 0.05 and FDR < 0.05.

## Conflict of Interest

The authors declare no conflict of interest.

## Author Contributions

D.L., H.Z., Y.Y., T.L., and Z.G. contributed equally to this work. Z.Z. and S.H. conceived and coordinated the study. Z.Z., Z.G., D.L., T.L., W.F., D.Y., S.Y., B.Z., J.C., J.T., Ji.H., and K.G. constructed the segregating population and collected the phenotype data. D.L. and H.Z. carried out the hydrophilic metabolite and lipid analyses. T.L., Y.Y., and D.L. carried out the volatile analysis. D.L., H.Z., T.L., H.T., Z.W., B.Z., H.L., Q.M., and X.Y. carried out the bioinformatics and experimental analyses. D.L., Y.Y., and Z.Z. wrote the manuscript. All authors read and approved the final manuscript.

## Supporting information

Supporting Information

Supporting Information

Supporting Information

Supporting Information

## Data Availability

All sequences have been deposited in the Sequence Read Archive (https://www.ncbi.nlm.nih.gov/sra) under the accession codes PRJNA844232 and PRJNA859501. The SNPs, Code and GWAS results have also been deposited into Figshare database (https://figshare.com/projects/Meat_Metabolome/148516).
