# Supplementary material for: Metabolome‐Based Genome‐Wide Association Study of Duck Meat Leads to Novel Genetic and Biochemical Insights
Source: Adv Sci (Weinh). 2023 Apr 4;10(18):2300148. doi: 10.1002/advs.202300148 (PMC10288243; doi:10.1002/advs.202300148)
Supplement: Supplementary file 1 — Supporting Information [file ADVS-10-2300148-s004.pdf]

ADVANCED SCIENCE
Open Access

Supporting Information

for *Adv. Sci.*, DOI 10.1002/advs.202300148

Metabolome-Based Genome-Wide Association Study of Duck Meat Leads to Novel Genetic and Biochemical Insights

*Dapeng Liu, He Zhang, Youyou Yang, Tong Liu, Zhanbao Guo, Wenlei Fan, Zhen Wang, Xinting Yang, Bo Zhang, Hongfei Liu, Hehe Tang, Daxin Yu, Simeng Yu, Kai Gai, Qiming Mou, Junting Cao, Jian Hu, Jing Tang, Shuisheng Hou* and Zhengkui Zhou**

# Supplemental Information for

## Metabolome-based genome-wide association study of duck meat leads to novel genetic and biochemical insights

## This file includes:

Supplementary Figure 1-21

Supplementary text 1

## 1. List of Supplementary Figures


**Supplementary Figure 1** Breed information for Pekin duck and Liancheng duck.

**Supplementary Figure 2** The detailed population and sample design used in this study.

**Supplementary Figure 3** Classification of annotated metabolites identified in hydrophilic metabolites, lipids and volatiles of the 423 ducks.

**Supplementary Figure 4** The broad-sense heritability (H2) and the coefficient of variation (CV) results for hydrophilic metabolites, lipids and volatiles.

**Supplementary Figure 5** Partial least squares discriminant analysis (PLS-DA) based on the levels of hydrophilic metabolites, lipids and volatiles.

**Supplementary Figure 6** The metabolites and volatiles with increasing or decreasing trends from R1 to R7.

**Supplementary Figure 7** Differential hydrophilic metabolites, lipids and volatiles between Pekin ducks and Liancheng ducks at 6 weeks.

**Supplementary Figure 8** A heatmap depicting Pearson's correlation between annotated hydrophilic metabolites, lipids and volatiles.

**Supplementary Figure 9** Pathways for the formation of 3-octen-2-one and heptanal by fatty acid.

**Supplementary Figure 10** Correlations in the systematic relationship between differential hydrophilic metabolites, lipids and volatiles.

**Supplementary Figure 11** Distribution of significant signals for the mGWAS results and scanned the extreme genetic divergence between Pekin duck and Liancheng duck.

**Supplementary Figure 12** The mGWAS results identified 43 candidate genes modulating metabolites of potential meat nutritional or flavor importance.

**Supplementary Figure 13** The levels of *TMEM189* gene and protein expression.

**Supplementary Figure 14** Box plot of 2-pyrrolidone content of three genotypes at SNP Chr7: 4,810,144 and *AOX1* gene expression at seven developmental stages.

**Supplementary Figure 15** Fixation index ($F_{ST}$, gray dot) and ratio of nucleotide diversity ($\pi$, 5 kb window, 2.5 kb step) for Chr2: 16.80 Mbp-16.90 Mbp SNPs between Pekin duck (red line) and Liancheng duck (blue line).

**Supplementary Figure 16** Fixation index (Fst) and ratio of nucleotide diversity ($\pi$, 5kb windows, 2.5kb step) for candidate region of Chr2 and Chr7 between Pekin duck and Liancheng duck.

**Supplementary Figure 17** 49 carnosine and related metabolites in the m/z–retention time (RT) plane, a group of unidentified features sharing the RT region with histidine and its derivatives.

**Supplementary Figure 18** 7 unknown metabolites were annotated by mGWAS results and verified by high-resolution mass spectrometry.

**Supplementary Figure 19** GWAS results of 7 meat quality traits (lightness (L*), redness (a*), yellowness (b*), shear force, $pH_{24h}$, crude fat, and water loss).

**Supplementary Figure 20** The GWAS results showed that *RAP1GAP2* was significantly associated with yellowness (b*).

**Supplementary Figure 21** A heatmap depicting Pearson's correlation between metabolites and meat quality traits**.**

## 2. List of Supplementary Tables

**Supplementary Table 1**
List of 423 ducks used in the study.

**Supplementary Table 2**
Widely targeted hydrophilic metabolites analysis in meat.

**Supplementary Table 3**
Targeted lipids analysis in meat.

**Supplementary Table 4**
Volatile compounds analysis in meat.

**Supplementary Table 5**
The metabolites and volatiles with increasing or decreasing trends from R1 to R7.

**Supplementary Table 6**
Significantly changed metabolites and volatiles in meat between Pekin duck and Liancheng duck.

**Supplementary Table 7**
The list of total 174 important metabolites and volatiles in the meat between Pekin duck and Liancheng duck.

**Supplementary Table 8**
The levels of hydrophilic metabolites in Pekin duck and Liancheng duck at seven development stages.

**Supplementary Table 9**
The levels of lipids in Pekin duck and Liancheng duck at seven development stages.

**Supplementary Table 10**
The differential preferentially accumulated hydrophilic metabolites and lipids between Pekin duck and Liancheng duck at seven development stages.

**Supplementary Table 11**
The pearson's correlation between secondary categories of hydrophilic metabolites, lipids and volatiles.

**Supplementary Table 12**
The list of total 1696 significant SNPs detected in hydrophilic metabolites.

**Supplementary Table 13**
The list of total 126 significant SNPs detected in lipids.

**Supplementary Tables 14**
The list of total 1040 significant SNPs detected in volatiles.

**Supplementary Table 15**
The list of 1063 loci detected in the meat of 423 duck populations by mGWAS.

**Supplementary Table 16**
241 mGWAS signals that were repeatedly detected.

**Supplementary Table 17**
Statistics of significant signals on the autosomes.

**Supplementary Table 18**
The top 1% genetic divergence region overlap with mGWAS signals.

**Supplementary Table 19**
Expression of 48 candidate genes in 9 different tissues of Pekin duck.

**Supplementary Table 20**
Expression of 48 Candidate genes in breast muscle in Pekin duck and Liancheng duck at seven development stages.

**Supplementary Table 21**
The expression of *AOX1* in breast muscle.

**Supplementary Table 22**
Details of the three haplotypes of *ACBD5* in 423 ducks.

**Supplementary Table 23**
The expression of *ACBD5* in breast muscle of three haplotypes.

**Supplementary Table 24**
The levels of 49 metabolites in R1-R7 line.

**Supplementary Table 25**
The expression of *GADL1* in breast muscle.

**Supplementary Table 26**
The expression of *CARNMT2* in breast muscle.

**Supplementary Table 27**
The mass spectrometry information of 7 metabolites annotated by mGWAS.

**Supplementary Table 28**
Composition of feed from hatch to 14 days of age and basal diet from 15 to 42 days of age.

**Supplementary Table 29**
Primers used in this study.

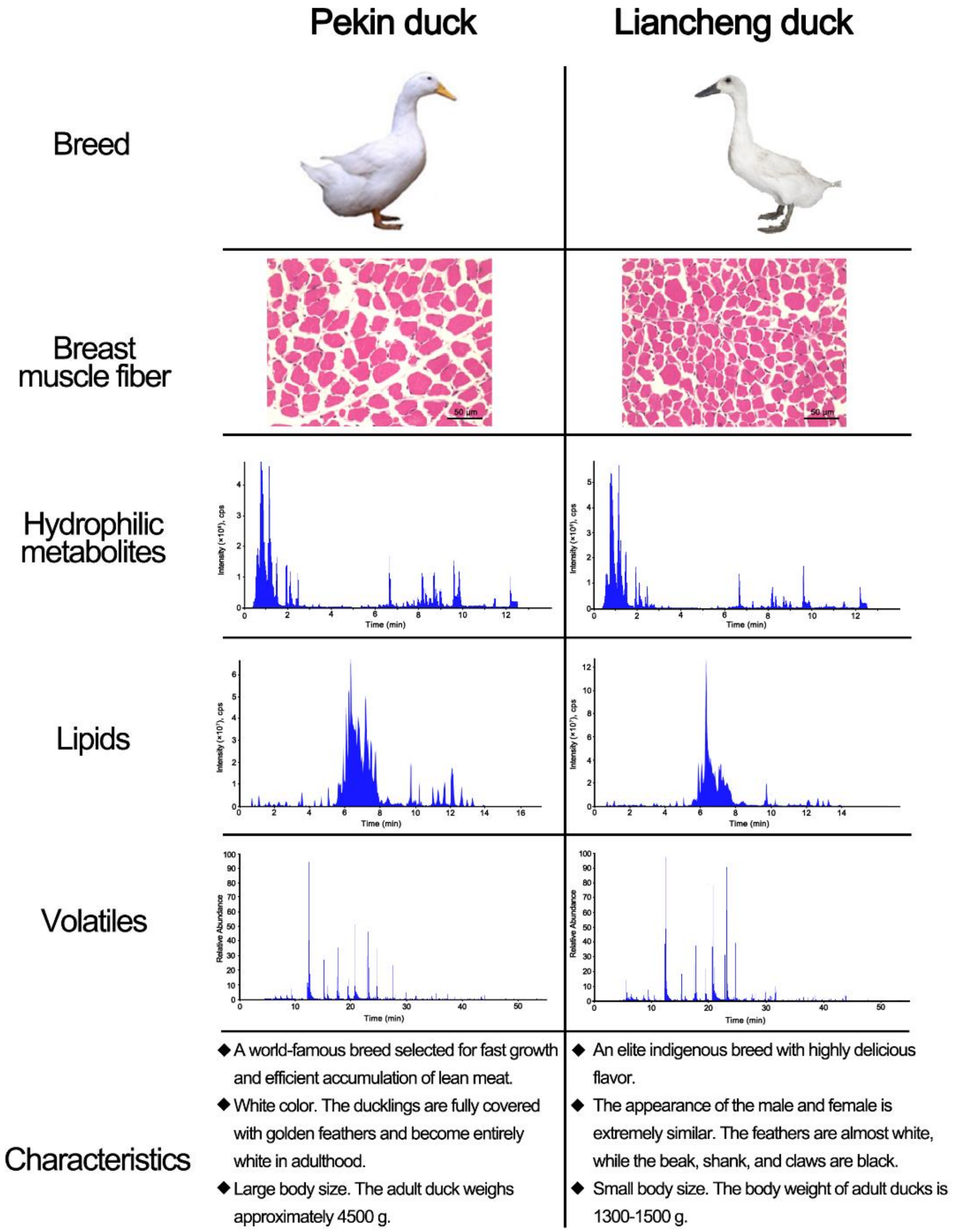


**Supplementary Figure 1** Breed information for Pekin duck and Liancheng duck. The differences characteristics between Pekin duck and Liancheng duck, including breast muscle fiber trait, hydrophilic metabolites were analyzed by LC–MS/MS and volatiles were analyzed by SPME-GC–MS.

a

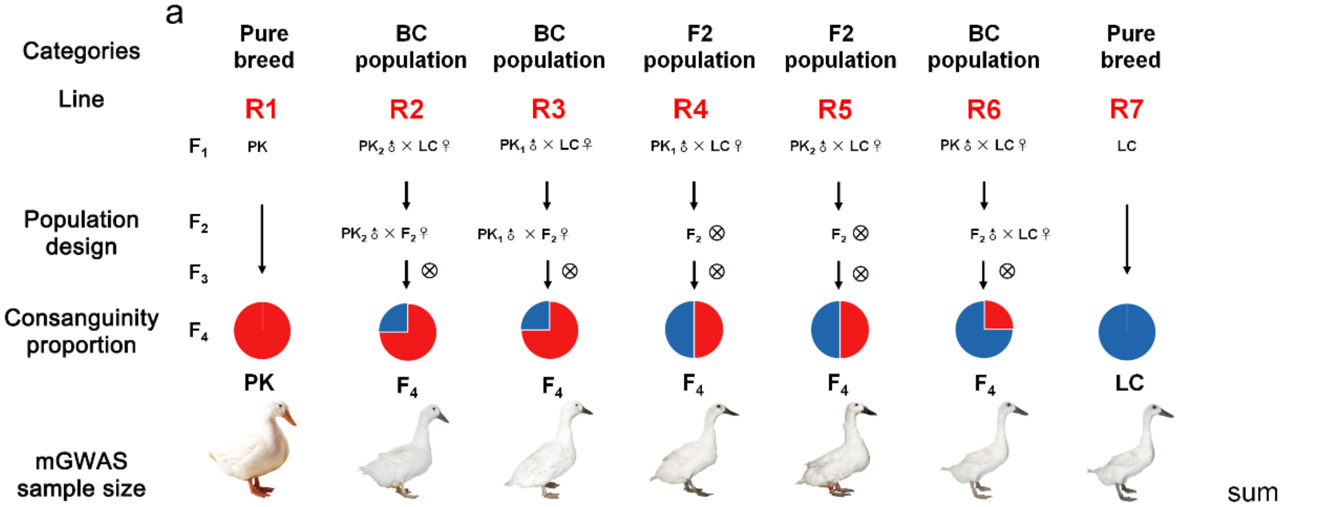


| | R1 | R2 | R3 | R4 | R5 | R6 | R7 | sum |
|---|---|---|---|---|---|---|---|---|
| n | 30 | 74 | 75 | 75 | 75 | 64 | 30 | 423 |
| ♂ | 18 | 33 | 37 | 38 | 41 | 32 | 15 | 214 |
| ♀ | 12 | 41 | 38 | 37 | 34 | 32 | 15 | 209 |

b

RNA-seq samples of 7 development stages

| Time | 1d | 1w | 2w | 5w | 4w | 5w | 6w |
|---|---|---|---|---|---|---|---|
| Pekin duck | 3 | 3 | 3 | 3 | 3 | 3 | 3 |
| Liancheng duck | 3 | 3 | 3 | 3 | 3 | 3 | 3 |

c

Hydrophilic metabolites and Lipids samples of 7 development stages

| Time | 1d | 1w | 2w | 3w | 4w | 5w | 6w |
|---|---|---|---|---|---|---|---|
| Pekin duck | 5 | 5 | 5 | 5 | 5 | 5 | 5 |
| Liancheng duck | 5 | 5 | 5 | 5 | 5 | 5 | 5 |

**Supplementary Figure 2** The detailed population and sample design used in this study.

(a) Gradient consanguinity design used for the two purebreds and the fourth generation of a large segregating duck population used for the genetic and biochemical basis of metabolites and volatiles. (b) Detailed information on the 7 developmental stages of RNA-seq samples used in this study. (c) Detailed information on the 7 development stages of hydrophilic metabolites and lipid samples used in this study.

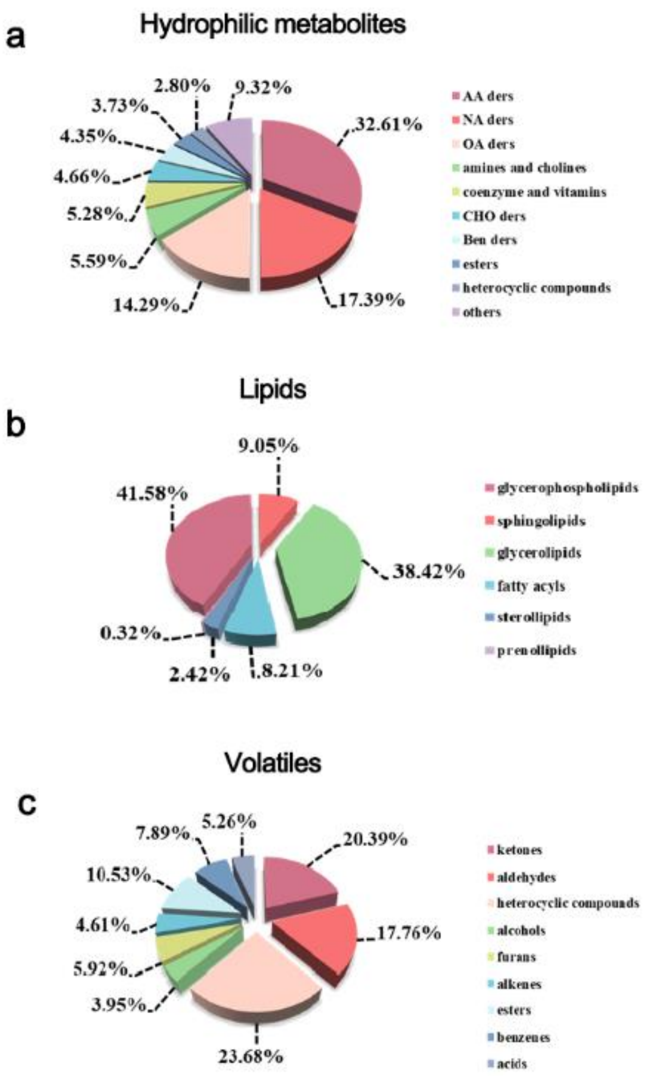


**Supplementary Figure 3** Classification of annotated metabolites identified in hydrophilic metabolites, lipids and volatiles of the 423 ducks.

(a) 10 subclasses of 321 annotated metabolites identified in hydrophilic metabolites. (b) 6 categories of 950 annotated metabolites identified in lipids. (c) 9 subclasses of 152 annotated metabolites identified in volatiles.

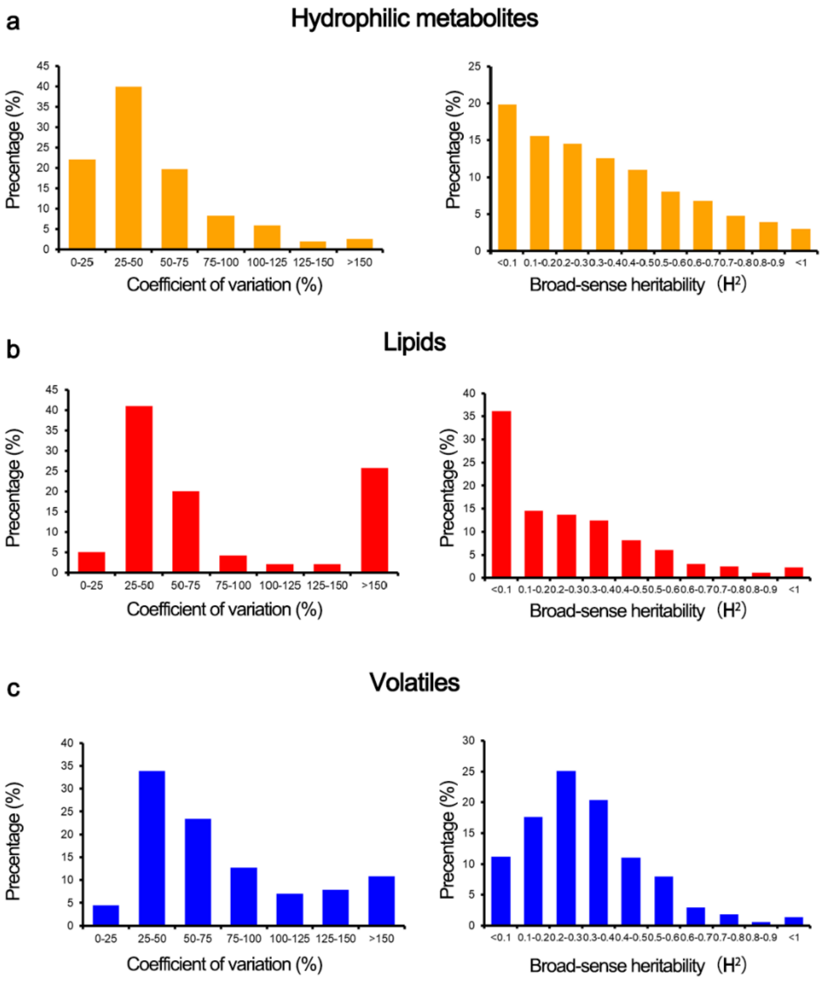


**Supplementary Figure 4** The broad-sense heritability (H2) and the coefficients of variation (CV) results for each metabolite.

(a) Distribution of broad-sense heritability ($H^2$) and coefficients of variation (CV) of hydrophilic metabolites (n= 2481). (b) Distribution of broad-sense heritability ($H^2$) and coefficients of variation (CV) of lipids (n= 950). (c) Distribution of broad-sense heritability ($H^2$) and coefficients of variation (CV) of volatiles (n= 702).

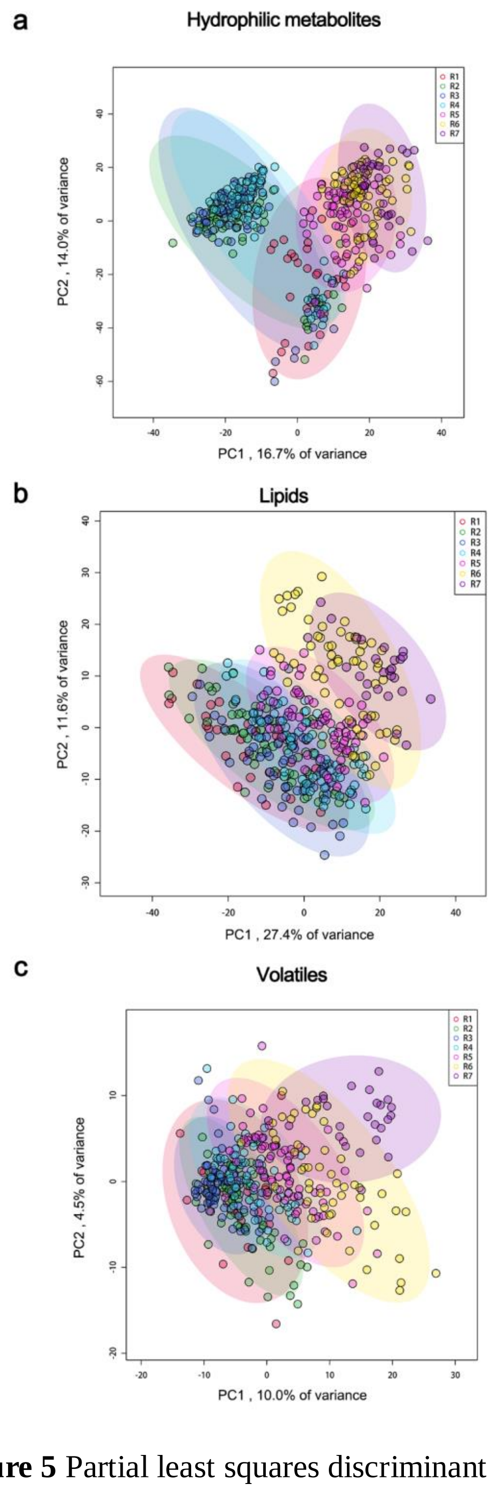


**Supplementary Figure 5** Partial least squares discriminant analysis (PLS-DA) based on the levels of hydrophilic metabolites, lipids and volatiles.

(a) 423 individuals could be divided into seven independent clusters by the levels of hydrophilic metabolites (n=2481). (b) 423 individuals could be divided into seven independent clusters by the levels of lipids (n= 950). (c) 423 individuals could be divided into seven independent clusters by the levels of volatiles (n= 702).

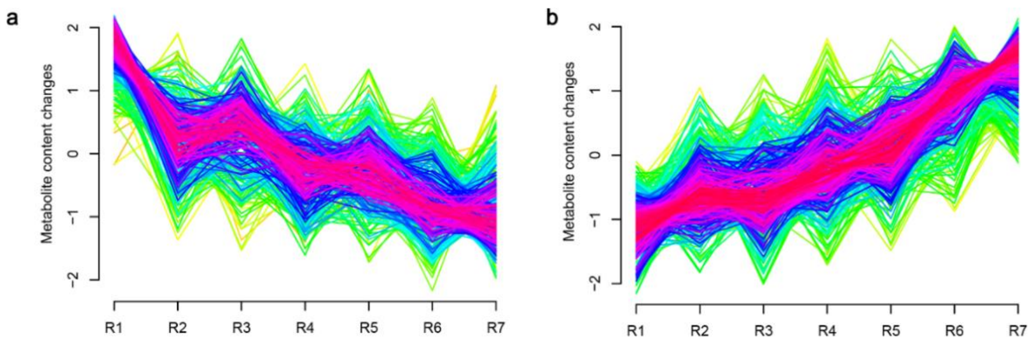


**Supplementary Figure 6** The metabolites and volatiles with increasing or decreasing trends from R1 to R7.

(a) The metabolites and volatiles showing a decreasing trend from R1 to R7. (b) The metabolites and volatiles showing an increasing trend from R1 to R7.

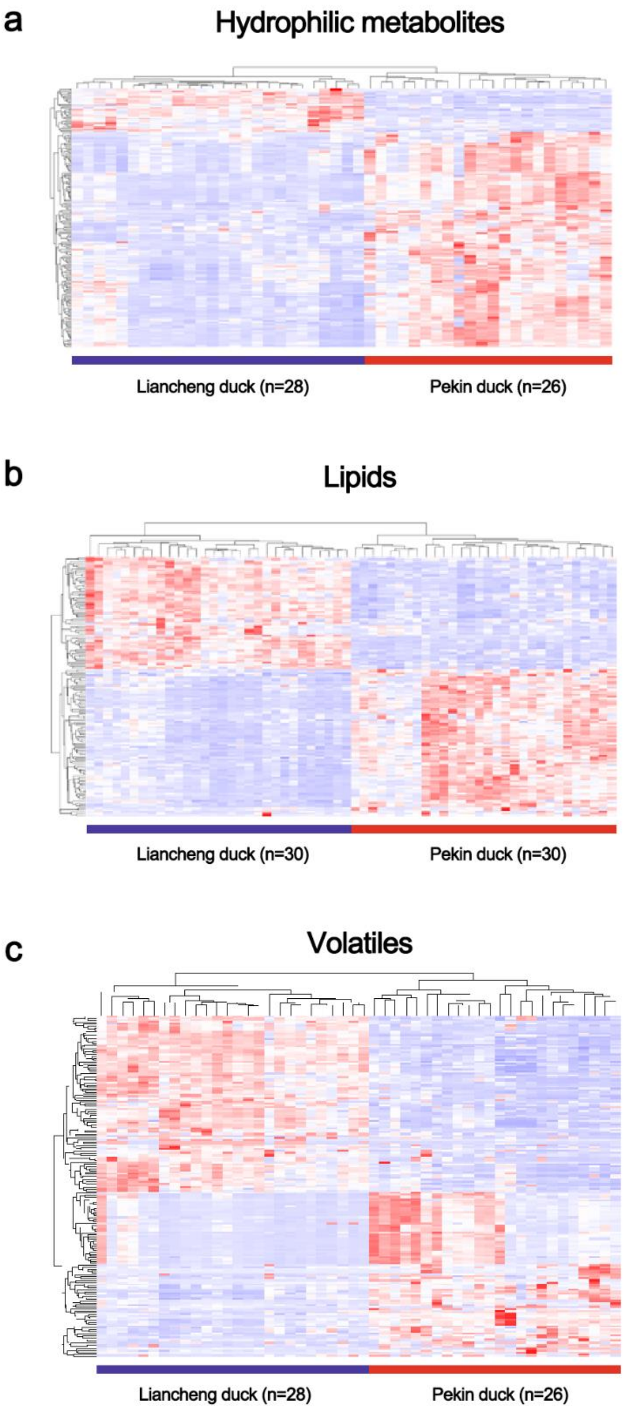


**Supplementary Figure 7** Differential hydrophilic metabolites, lipids and volatiles between Pekin ducks and Liancheng ducks at 6 weeks.

(a) 145 differential hydrophilic metabolites were identified. (b) 144 differential lipids were identified. (c) 180 differential volatiles were identified.

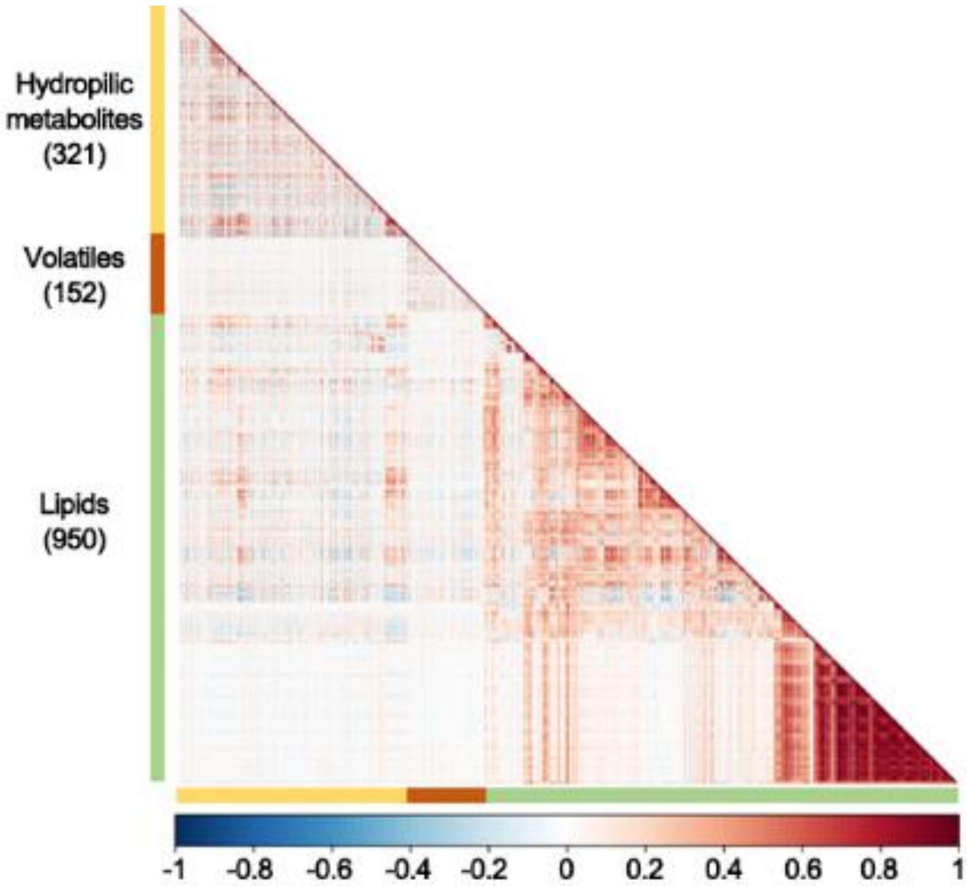


**Supplementary Figure 8** A heatmap depicting Pearson's correlation between annotated hydrophilic metabolites, lipids and volatiles.

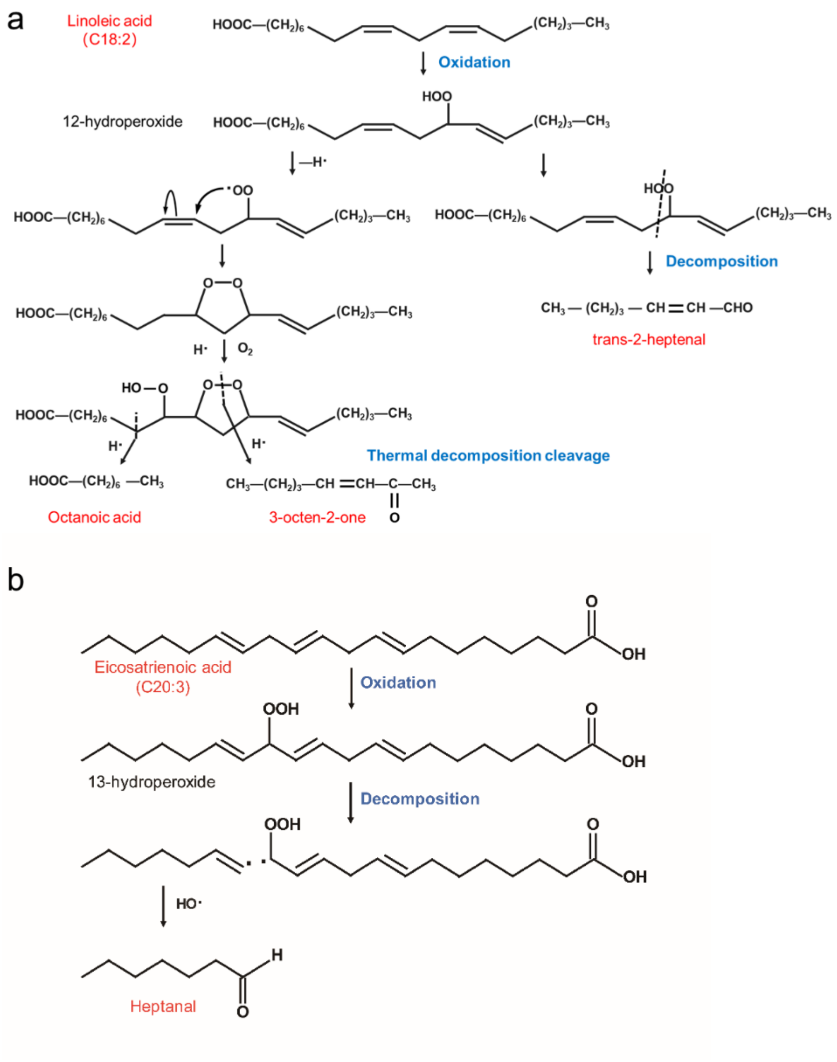


**Supplementary Figure 9** Pathways for the formation of heptanal and 3-octen-2-one. (a) Linoleic acid can be generated 3-octen-2-one by automatic oxidation and (b) Heptanal was generated by eicosatrienoic acid automatic oxidation and thermal decomposition cleavage.

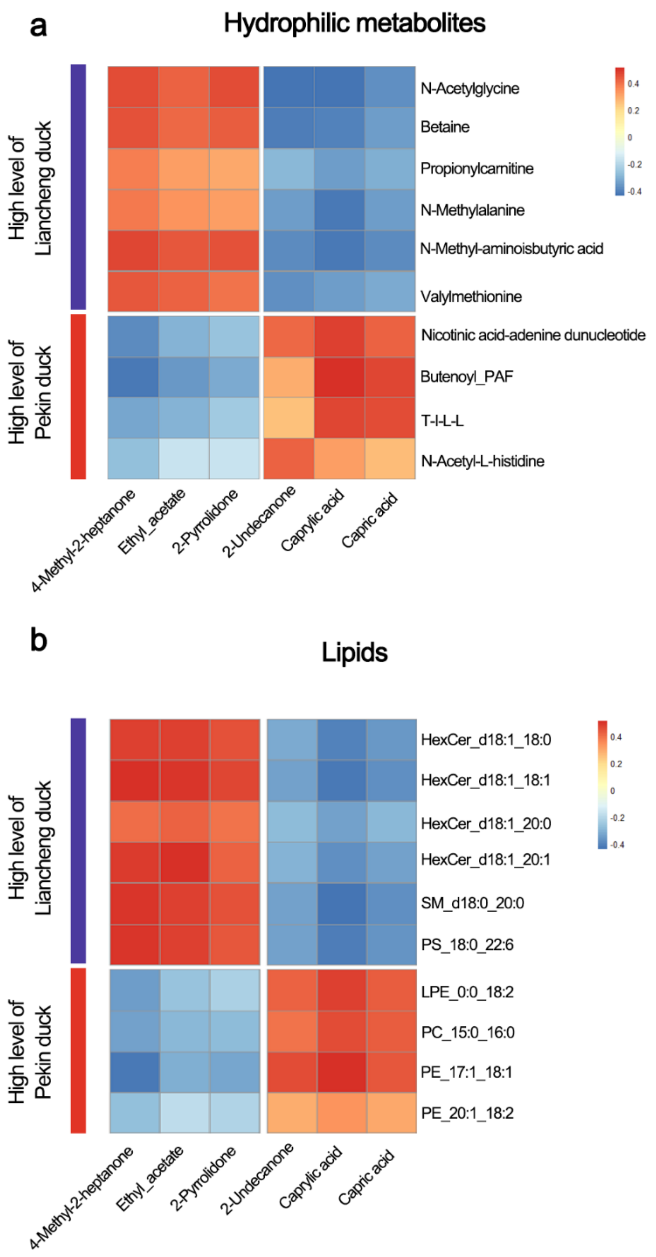


**Supplementary Figure 10** Correlations in the systematic relationship between differential hydrophilic metabolites, lipids and volatiles.

(a) Differential hydrophilic metabolites was correlated with characteristical volatiles between Pekin duck and Liancheng duck. (b) Differential lipids was correlated with characteristical volatiles between Pekin duck and Liancheng duck.

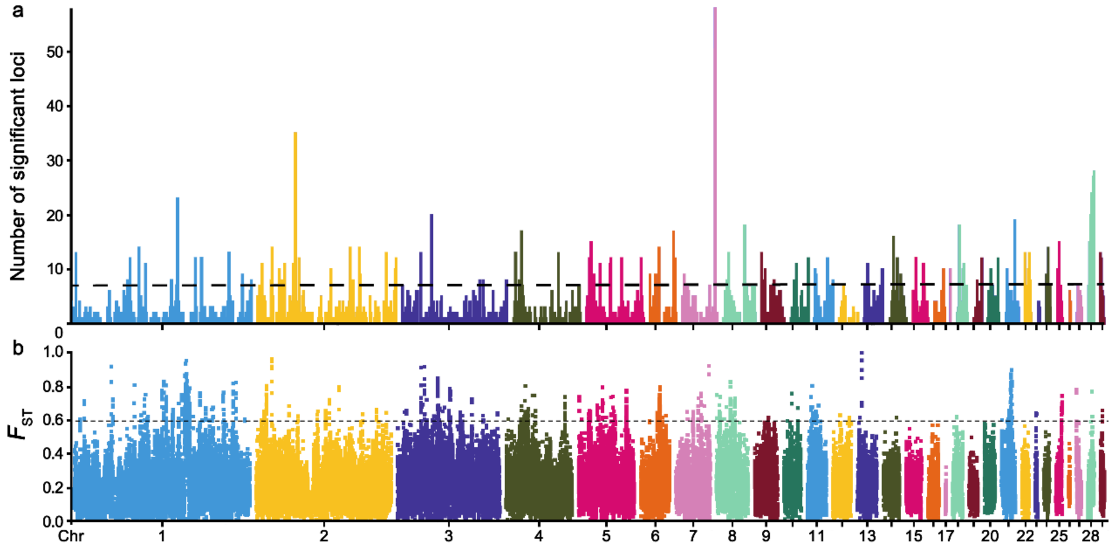


**Supplementary Figure 11** Distribution of significant signals for the mGWAS results and scanned the extreme genetic divergence between Pekin duck and Liancheng duck. (a) Statistics of the number of the significant signals for the mGWAS results, the horizontal dashed line indicates the threshold (permutation test < 0.01) for signals hotspots. (b) The genetic divergence between Pekin duck and Liancheng duck, the horizontal dashed line indicates the threshold (TOP 1%) for genetic divergence region.

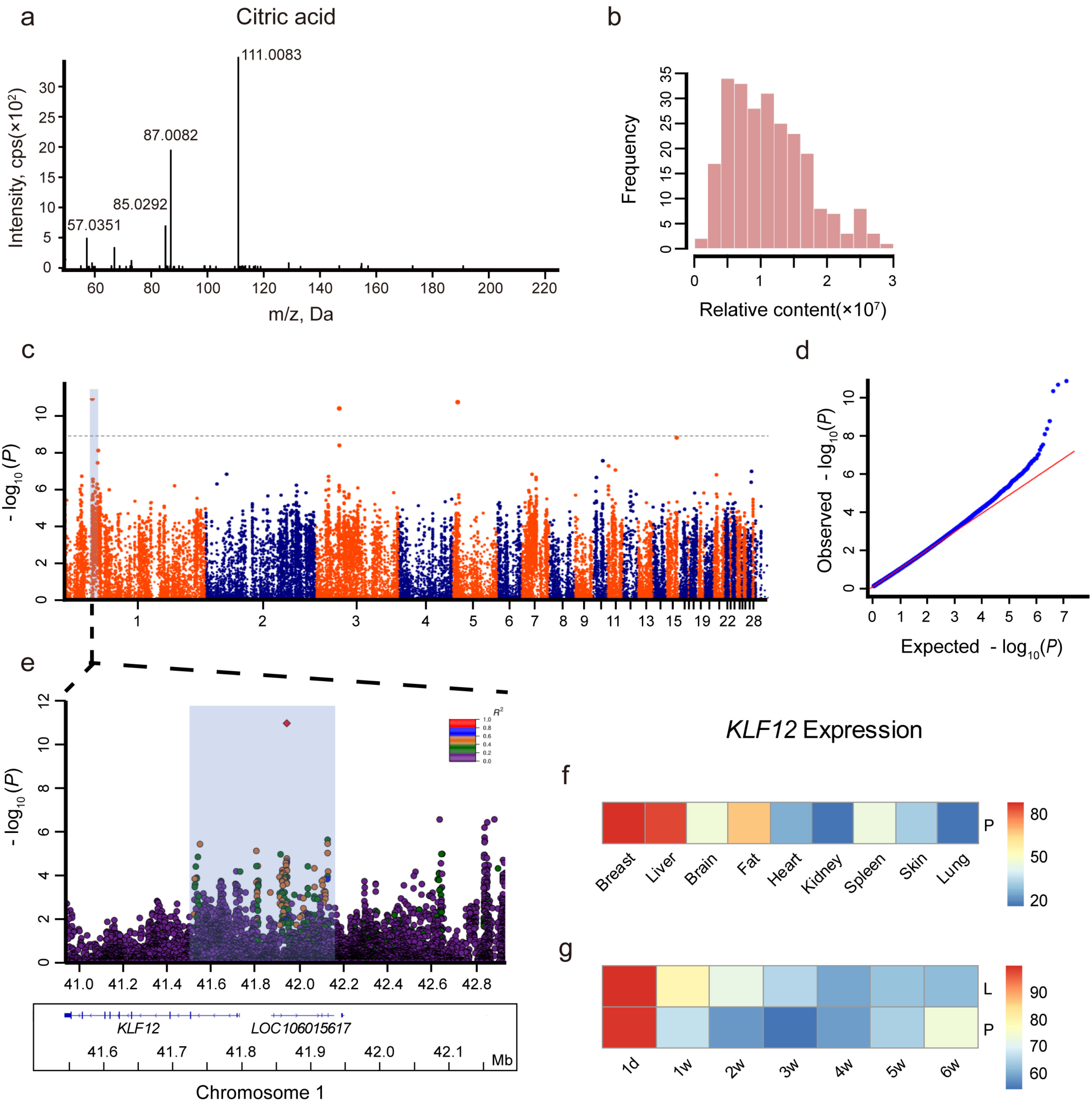
a
Citric acid
111.0083
87.0082
85.0292
57.0351
Intensity, cps(×10²)
m/z, Da
b
Frequency
Relative content(×10⁷)
c
- log10(P)
d
Observed - log10(P)
Expected - log10(P)
e
KLF12
LOC106015617
Mb
Chromosome 1
KLF12 Expression
f
Breast
Liver
Brain
Fat
Heart
Kidney
Spleen
Skin
Lung
P
g
L
P
1d
1w
2w
3w
4w
5w
6w

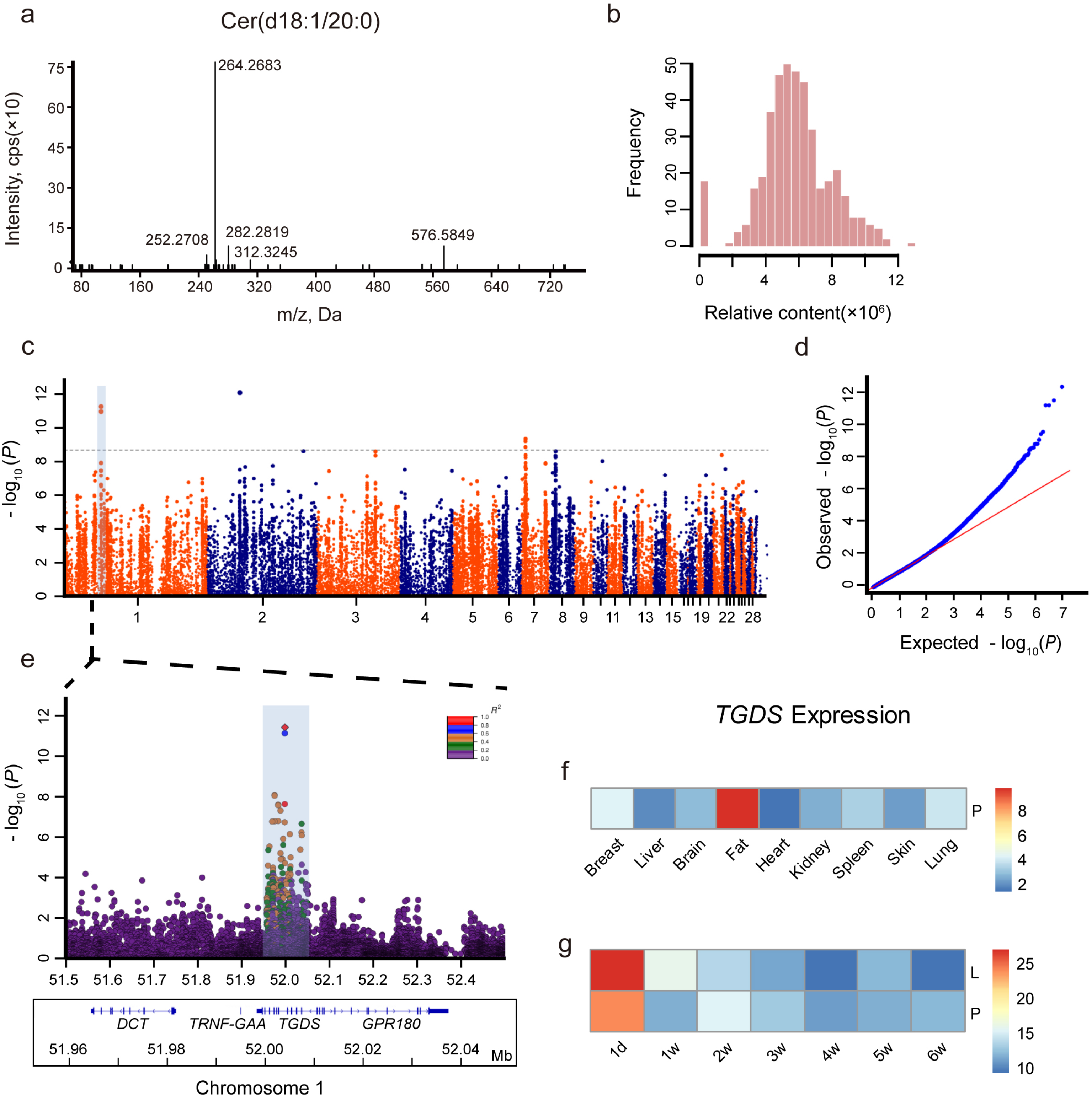
a
Cer(d18:1/20:0)
Intensity, cps(×10)
264.2683
252.2708
282.2819
312.3245
576.5849
m/z, Da
b
Frequency
Relative content(×10⁶)
c
- log10 (P)
d
Observed - log10(P)
Expected - log10(P)
e
DCT
TRNF-GAA
TGDS
GPR180
Mb
Chromosome 1
TGDS Expression
f
Breast
Liver
Brain
Fat
Heart
Kidney
Spleen
Skin
Lung
P
g
1d
1w
2w
3w
4w
5w
6w
L
P

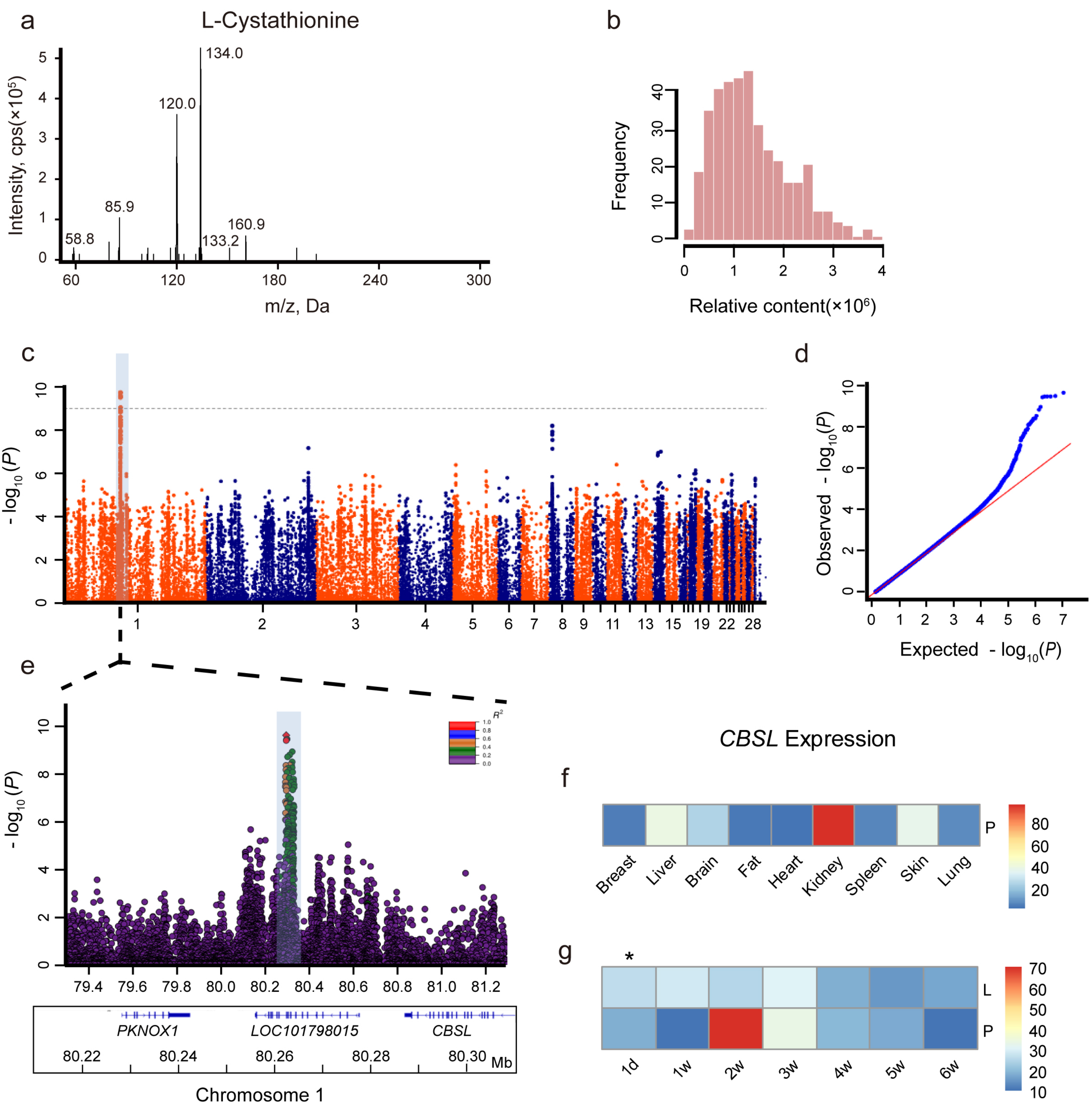

a
L-Cystathionine
Intensity, cps(×10⁵)
m/z, Da
134.0
120.0
85.9
160.9
58.8
133.2
b
Frequency
Relative content(×10⁶)
c
- log10(P)
d
Observed - log10(P)
Expected - log10(P)
e
R2
PKNOX1
LOC101798015
CBSL
Mb
Chromosome 1
CBSL Expression
f
Breast
Liver
Brain
Fat
Heart
Kidney
Spleen
Skin
Lung
P
g
*
L
P
1d
1w
2w
3w
4w
5w
6w

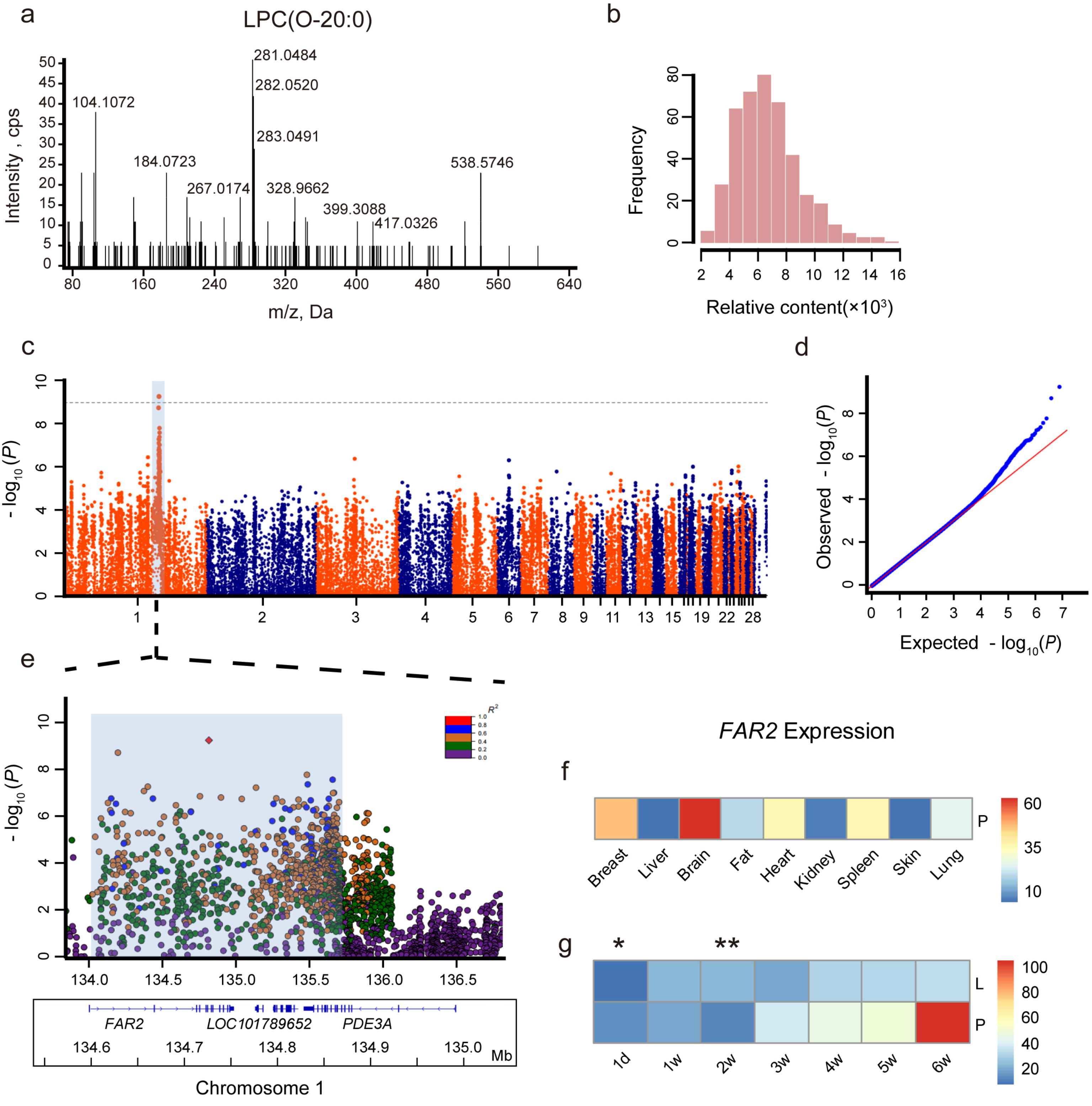

a
LPC(O-20:0)
Intensity , cps
m/z, Da
104.1072
184.0723
267.0174
281.0484
282.0520
283.0491
328.9662
399.3088
417.0326
538.5746
b
Frequency
Relative content(×10³)
c
- log10(P)
d
Observed - log10(P)
Expected - log10(P)
e
- log10(P)
FAR2
LOC101789652
PDE3A
Mb
Chromosome 1
FAR2 Expression
f
Breast
Liver
Brain
Fat
Heart
Kidney
Spleen
Skin
Lung
P
g
*
**
L
P
1d
1w
2w
3w
4w
5w
6w

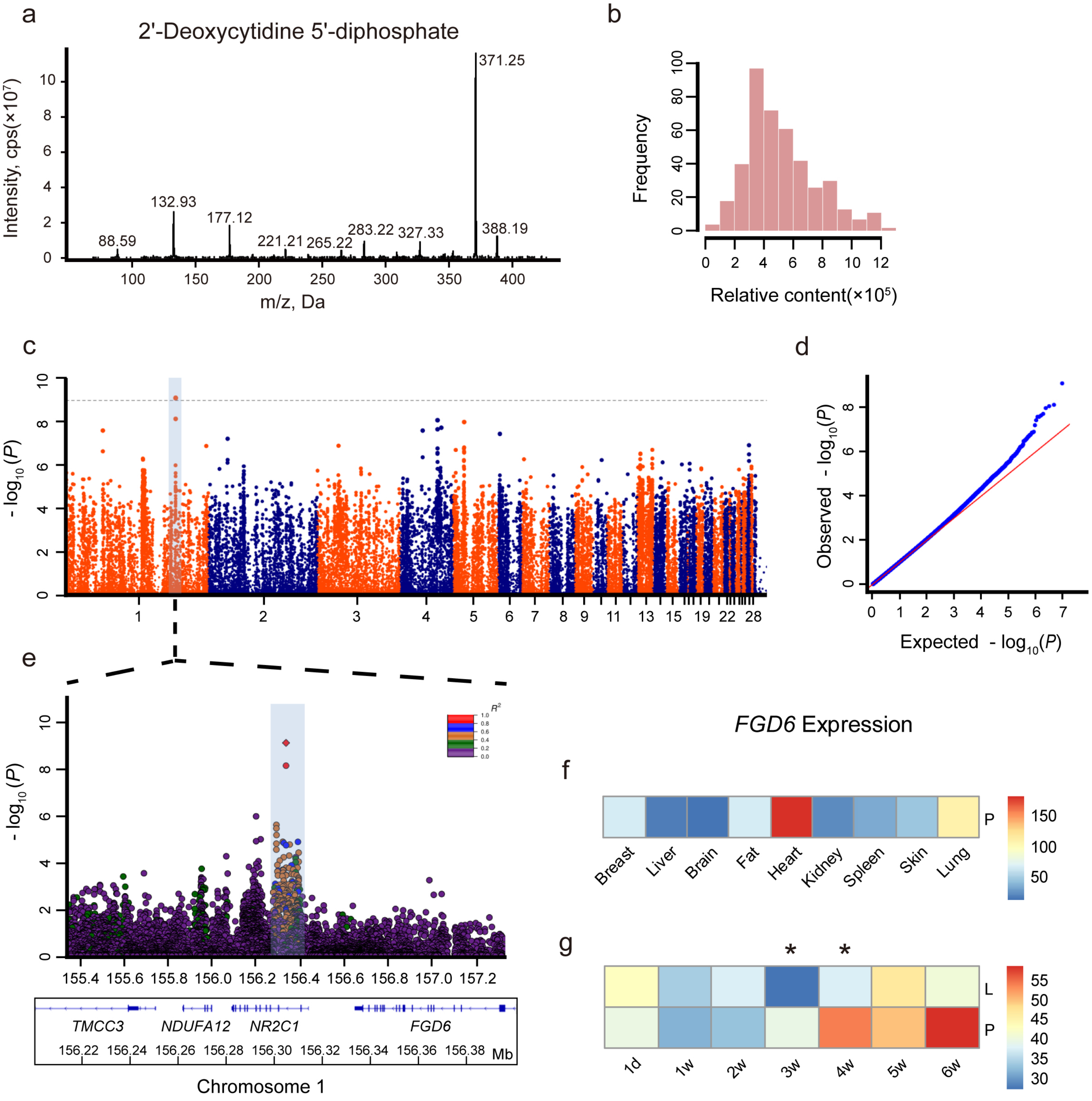

a
2'-Deoxycytidine 5'-diphosphate
Intensity, cps(×10^7)
m/z, Da
88.59
132.93
177.12
221.21
265.22
283.22
327.33
371.25
388.19
b
Frequency
Relative content(×10^5)
c
- log10(P)
d
Observed - log10(P)
Expected - log10(P)
e
- log10(P)
R^2
TMCC3
NDUFA12
NR2C1
FGD6
Mb
Chromosome 1
FGD6 Expression
f
Breast
Liver
Brain
Fat
Heart
Kidney
Spleen
Skin
Lung
P
g
L
P
1d
1w
2w
3w
4w
5w
6w

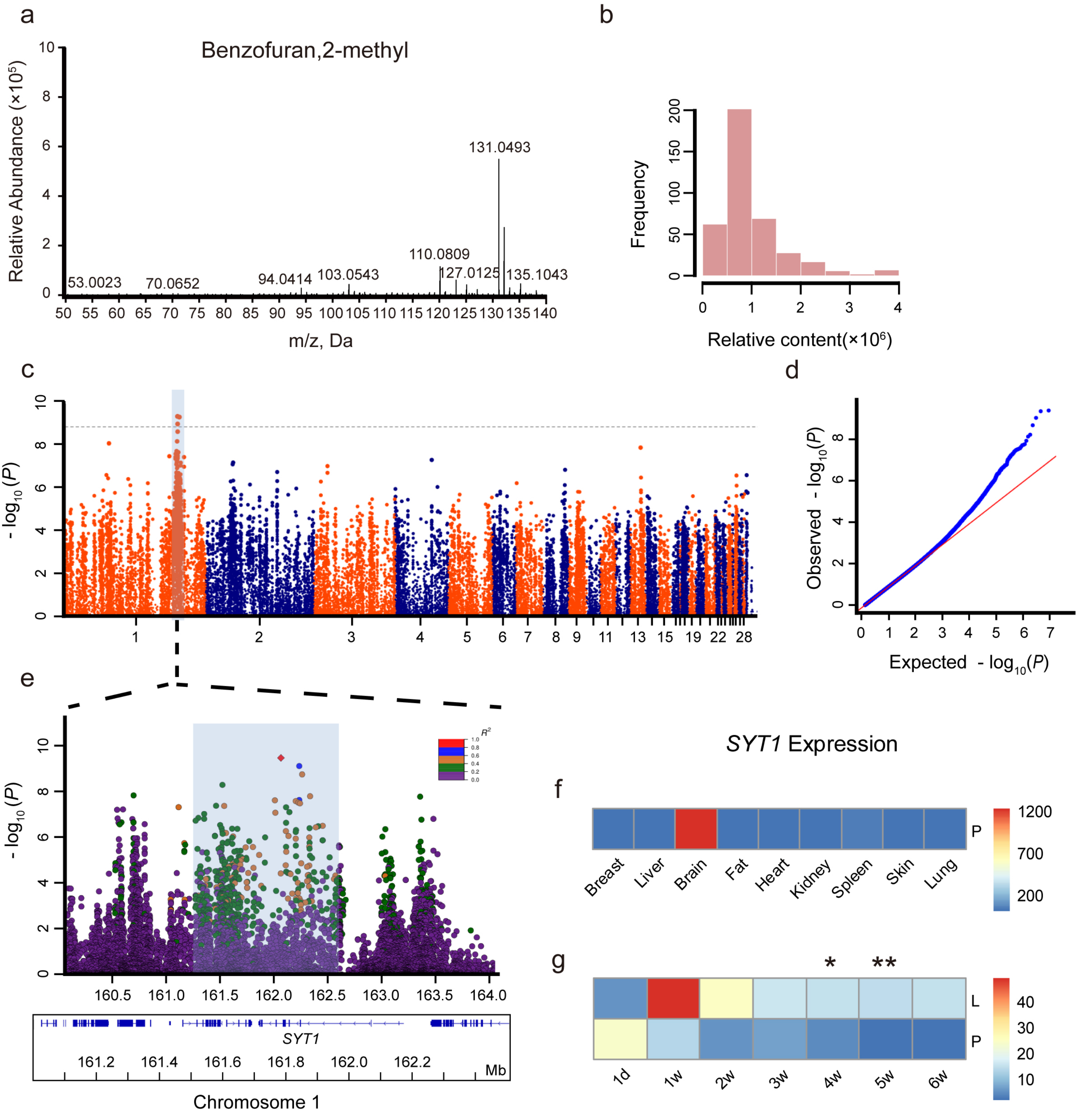

a
b
c
d
e
f
g
Benzofuran,2-methyl
Relative Abundance (×10⁵)
131.0493
110.0809
127.0125
135.1043
103.0543
94.0414
70.0652
53.0023
m/z, Da
Frequency
Relative content(×10⁶)
- log10(P)
Observed - log10(P)
Expected - log10(P)
SYT1
Chromosome 1
Mb
SYT1 Expression
Breast
Liver
Brain
Fat
Heart
Kidney
Spleen
Skin
Lung
P
L
1d
1w
2w
3w
4w
5w
6w
*
**

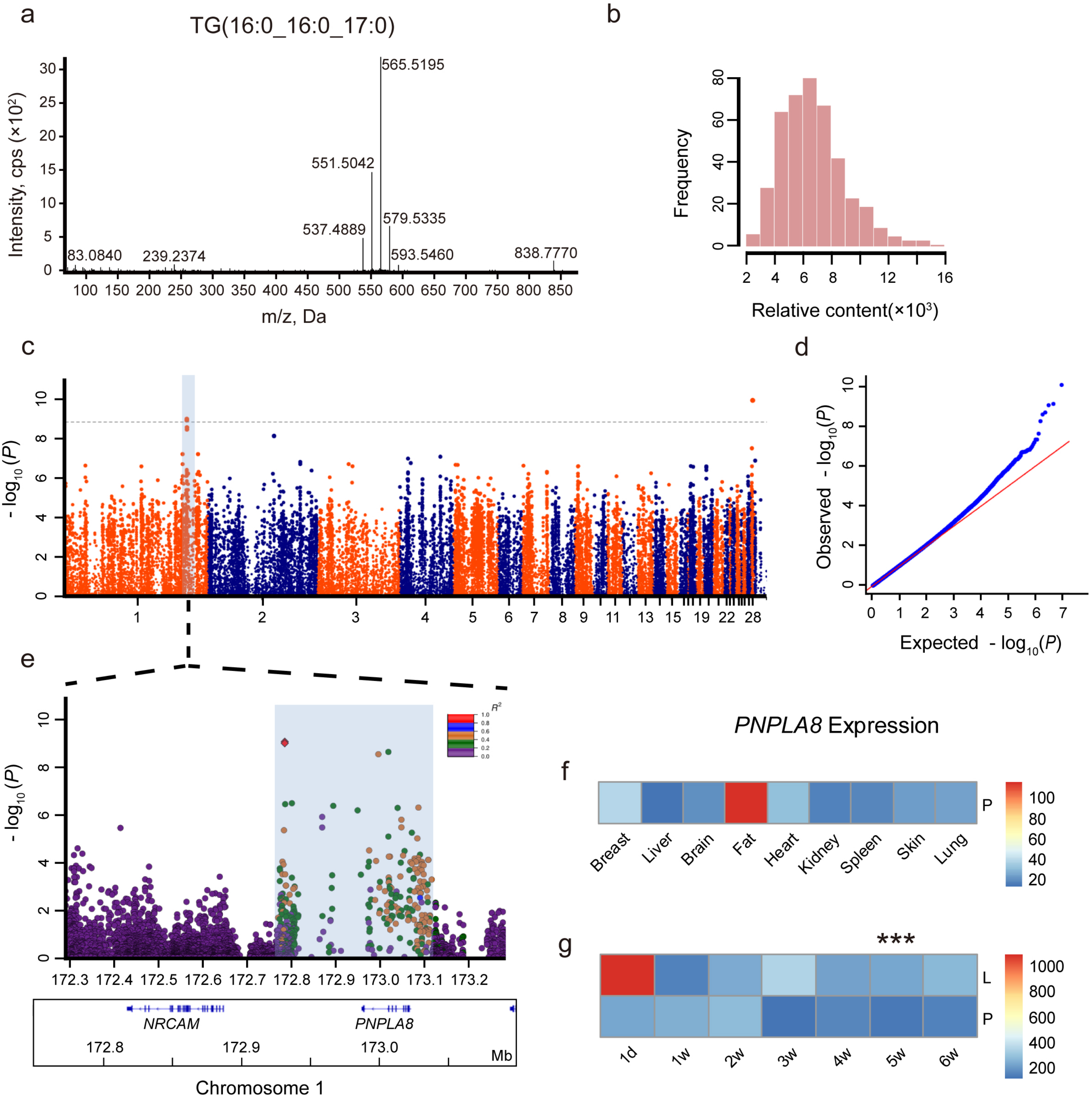

a
TG(16:0_16:0_17:0)
Intensity, cps (×10²)
565.5195
551.5042
537.4889
579.5335
593.5460
838.7770
83.0840
239.2374
m/z, Da
b
Frequency
Relative content(×10³)
c
- log10(P)
d
Observed - log10(P)
Expected - log10(P)
e
NRCAM
PNPLA8
Mb
Chromosome 1
PNPLA8 Expression
f
Breast
Liver
Brain
Fat
Heart
Kidney
Spleen
Skin
Lung
P
g
***
L
P
1d
1w
2w
3w
4w
5w
6w

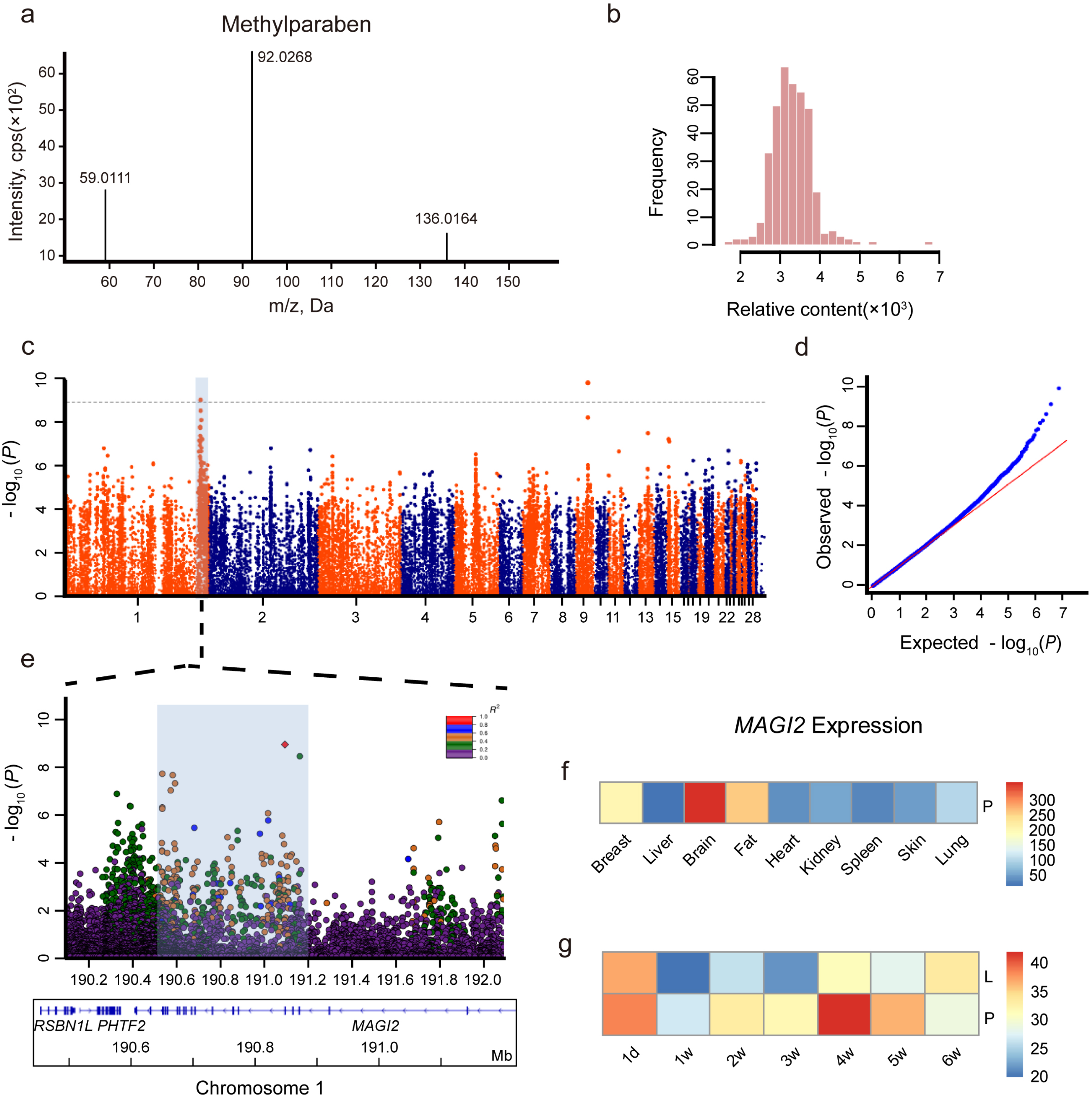
a
Methylparaben
92.0268
59.0111
136.0164
Intensity, cps(×10²)
m/z, Da
b
Frequency
Relative content(×10³)
c
- log10(P)
d
Observed - log10(P)
Expected - log10(P)
e
R²
- log10(P)
RSBN1L PHTF2
MAGI2
Mb
Chromosome 1
MAGI2 Expression
f
Breast
Liver
Brain
Fat
Heart
Kidney
Spleen
Skin
Lung
P
g
1d
1w
2w
3w
4w
5w
6w
L
P

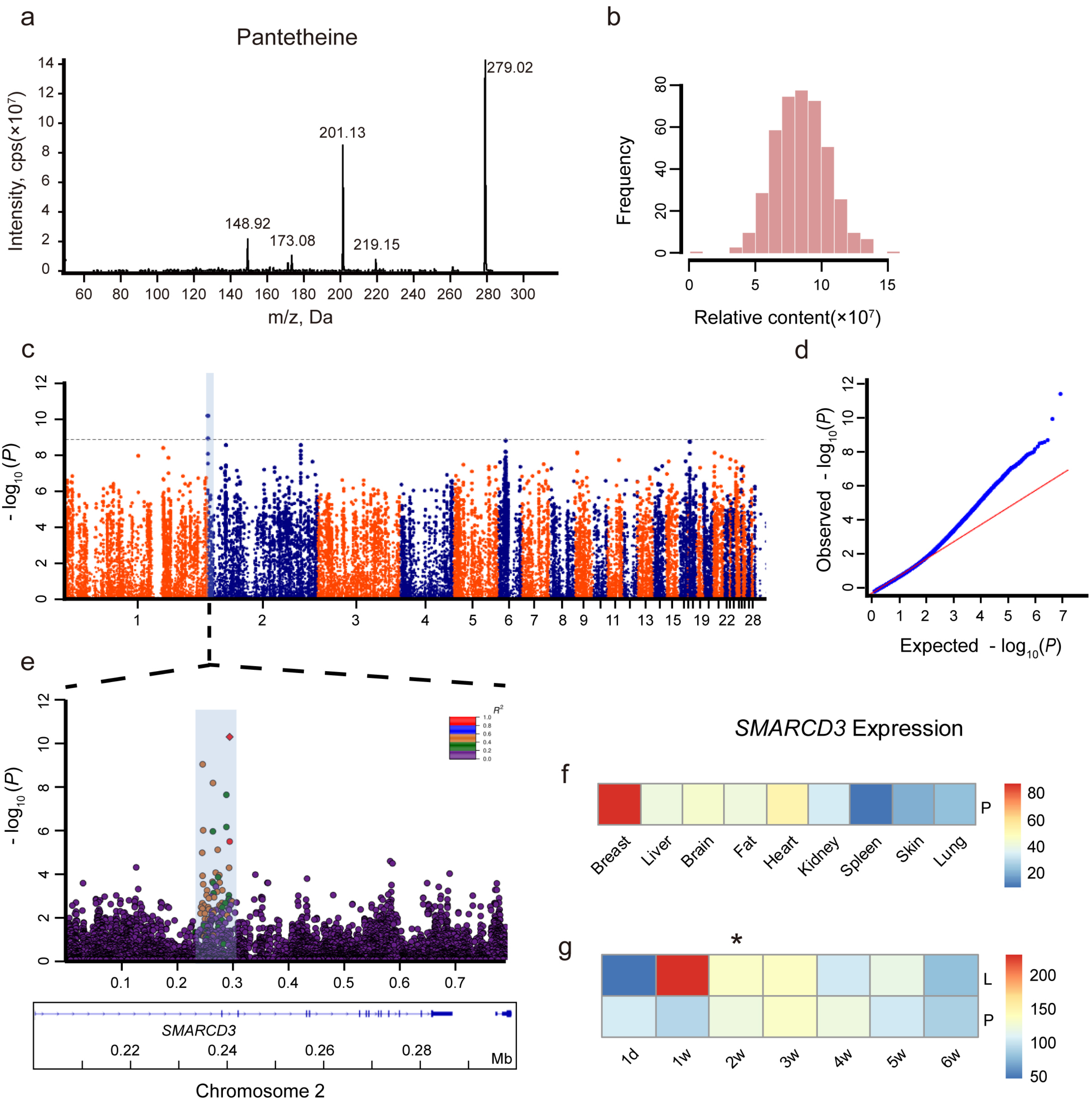

a
Pantetheine
Intensity, cps(×10^7)
279.02
201.13
148.92
173.08
219.15
m/z, Da
b
Frequency
Relative content(×10^7)
c
- log10(P)
d
Observed - log10(P)
Expected - log10(P)
e
SMARCD3
Chromosome 2
Mb
SMARCD3 Expression
f
Breast
Liver
Brain
Fat
Heart
Kidney
Spleen
Skin
Lung
P
g
L
P
1d
1w
2w
3w
4w
5w
6w

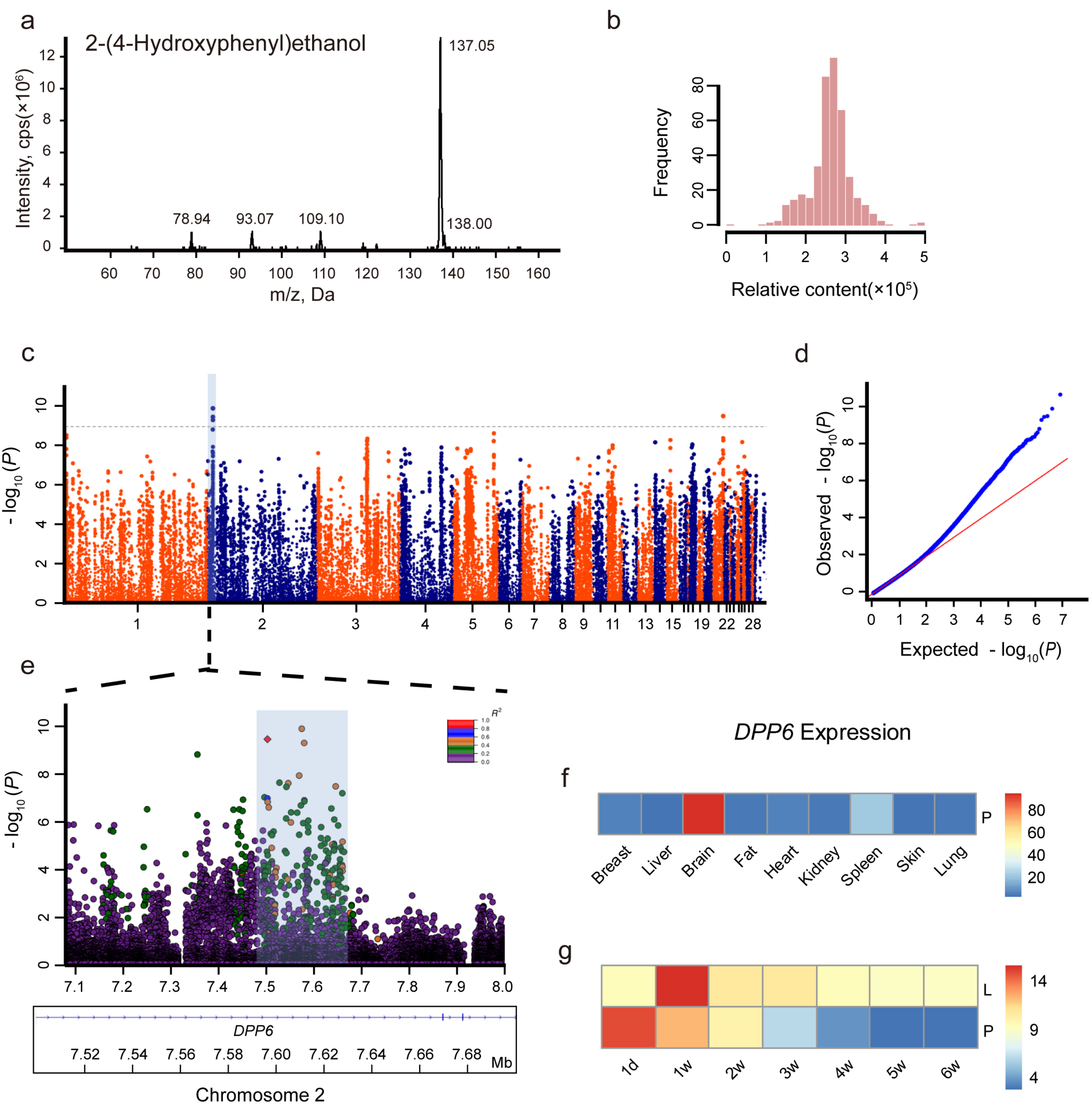
a
2-(4-Hydroxyphenyl)ethanol
Intensity, cps(×10⁶)
137.05
78.94
93.07
109.10
138.00
m/z, Da
b
Frequency
Relative content(×10⁵)
c
- log10(P)
d
Observed - log10(P)
Expected - log10(P)
e
DPP6
Chromosome 2
Mb
DPP6 Expression
f
Breast
Liver
Brain
Fat
Heart
Kidney
Spleen
Skin
Lung
P
g
L
P
1d
1w
2w
3w
4w
5w
6w
