# Supplementary material for: Metabolome‐Based Genome‐Wide Association Study of Duck Meat Leads to Novel Genetic and Biochemical Insights
Source: Adv Sci (Weinh). 2023 Apr 4;10(18):2300148. doi: 10.1002/advs.202300148 (PMC10288243; doi:10.1002/advs.202300148)
Supplement: Supplementary file 2 — Supporting Information [file ADVS-10-2300148-s003.pdf]

ADVANCED SCIENCE
Open Access

Supporting Information

for *Adv. Sci.*, DOI 10.1002/advs.202300148

Metabolome-Based Genome-Wide Association Study of Duck Meat Leads to Novel Genetic and Biochemical Insights

Dapeng Liu, He Zhang, Youyou Yang, Tong Liu, Zhanbao Guo, Wenlei Fan, Zhen Wang, Xinting Yang, Bo Zhang, Hongfei Liu, Hehe Tang, Daxin Yu, Simeng Yu, Kai Gai, Qiming Mou, Junting Cao, Jian Hu, Jing Tang, Shuisheng Hou and Zhengkui Zhou**

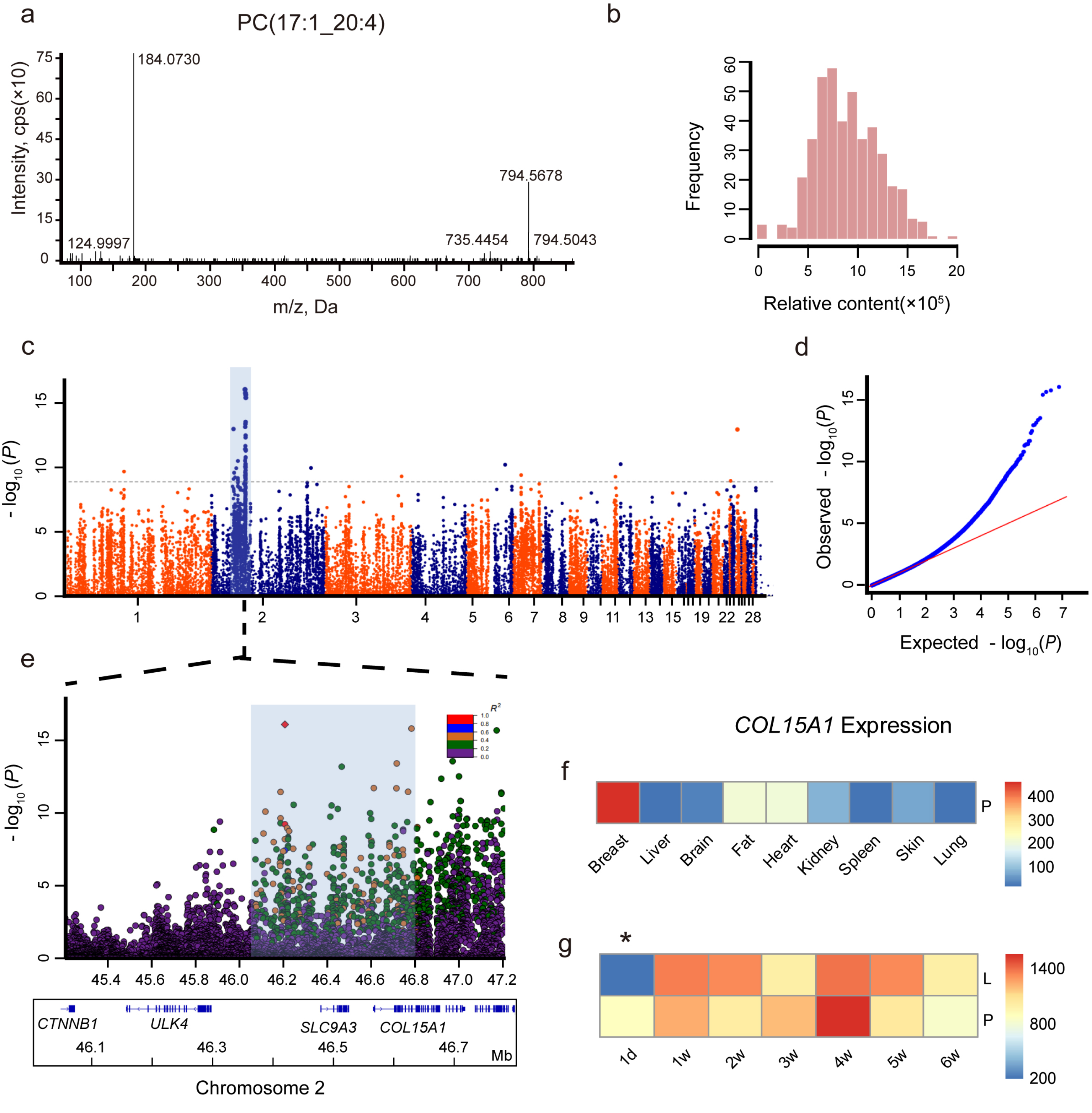

a
PC(17:1_20:4)
Intensity, cps(×10)
184.0730
794.5678
735.4454
794.5043
124.9997
m/z, Da
b
Frequency
Relative content(×10⁵)
c
- log10 (P)
d
Observed - log10(P)
Expected - log10(P)
e
R²
CTNNB1
ULK4
SLC9A3
COL15A1
Mb
Chromosome 2
COL15A1 Expression
f
Breast
Liver
Brain
Fat
Heart
Kidney
Spleen
Skin
Lung
P
g
*
L
P
1d
1w
2w
3w
4w
5w
6w

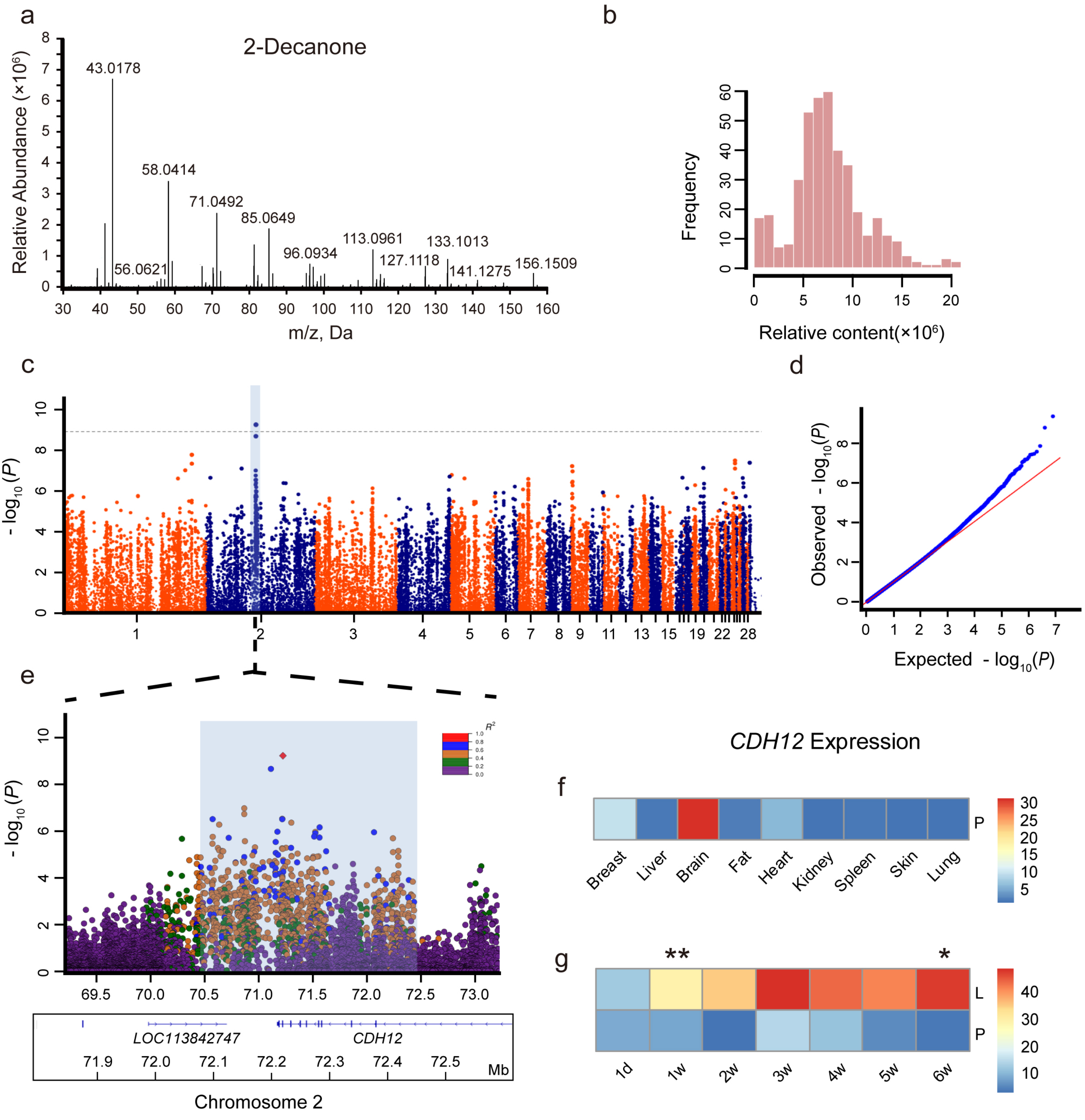
a
2-Decanone
Relative Abundance (×10⁶)
43.0178
58.0414
71.0492
85.0649
96.0934
113.0961
127.1118
133.1013
141.1275
156.1509
56.0621
m/z, Da
b
Frequency
Relative content(×10⁶)
c
- log10(P)
d
Observed - log10(P)
Expected - log10(P)
e
- log10(P)
R2
LOC113842747
CDH12
Mb
Chromosome 2
CDH12 Expression
f
Breast
Liver
Brain
Fat
Heart
Kidney
Spleen
Skin
Lung
P
g
**
*
L
P
1d
1w
2w
3w
4w
5w
6w

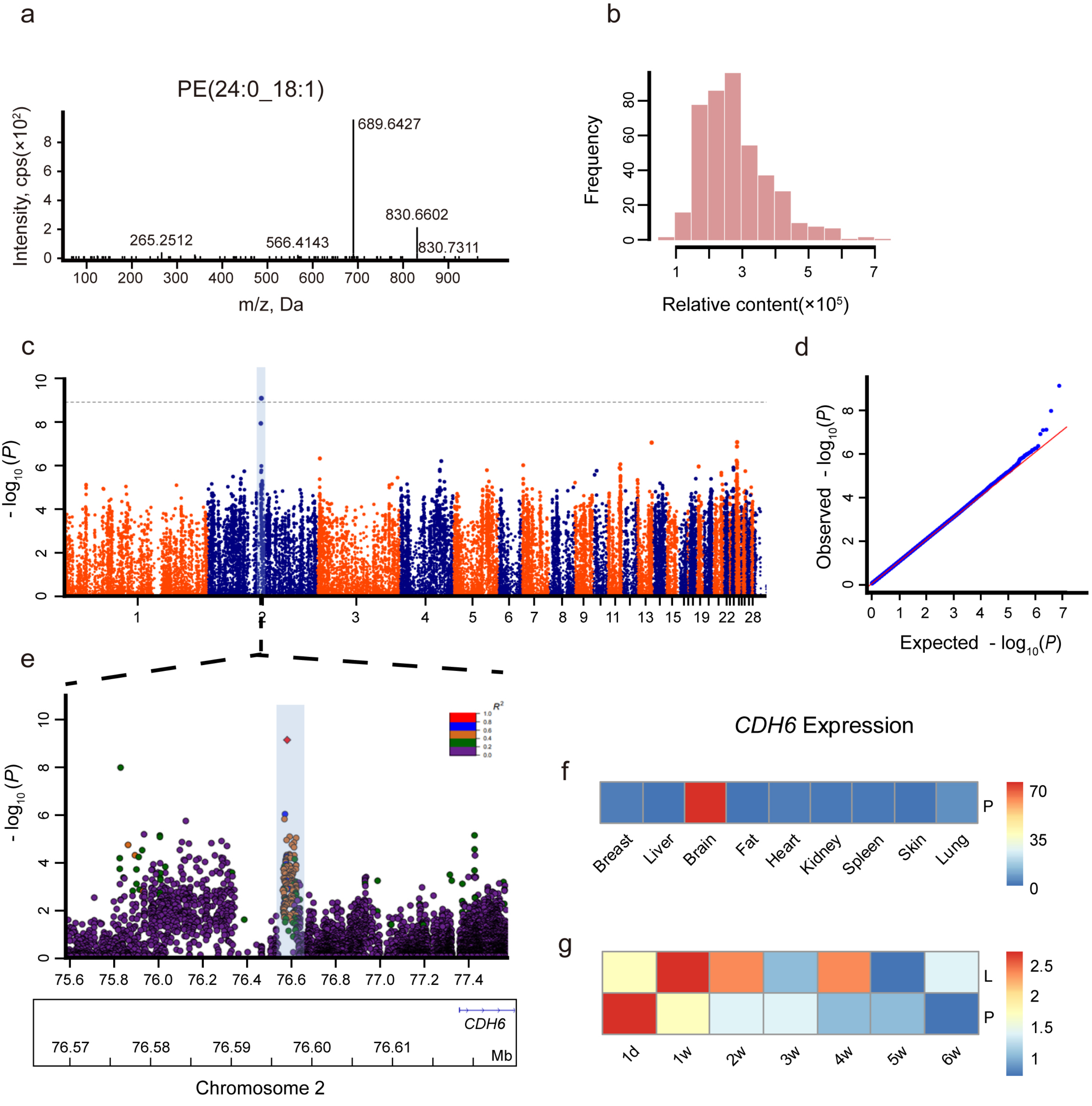
a
PE(24:0_18:1)
Intensity, cps(×10²)
689.6427
830.6602
265.2512
566.4143
830.7311
m/z, Da
b
Frequency
Relative content(×10⁵)
c
- log10 (P)
d
Observed - log10(P)
Expected - log10(P)
e
- log10 (P)
R²
CDH6
Mb
Chromosome 2
CDH6 Expression
f
Breast
Liver
Brain
Fat
Heart
Kidney
Spleen
Skin
Lung
P
g
L
P
1d
1w
2w
3w
4w
5w
6w

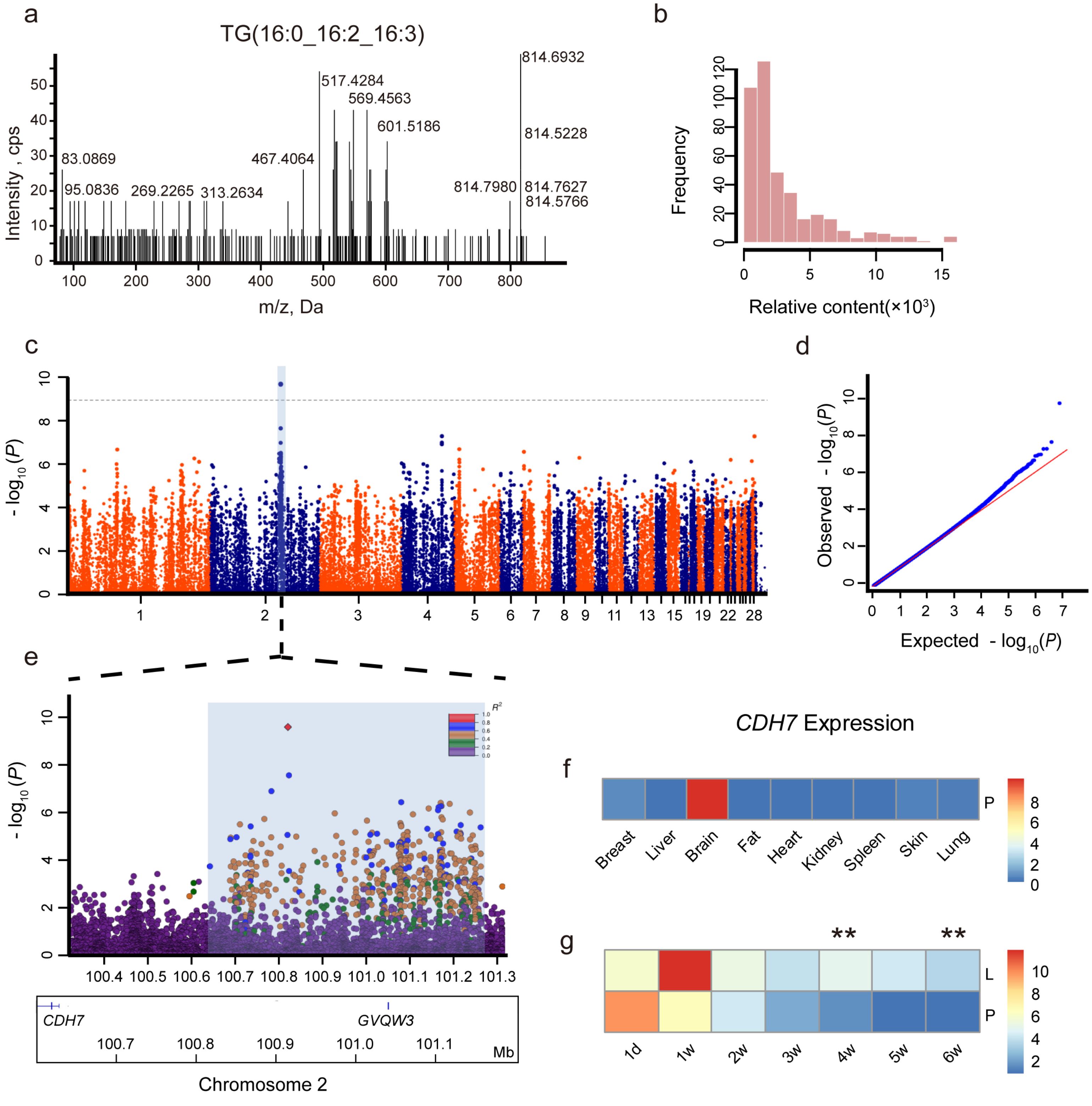
a
TG(16:0_16:2_16:3)
Intensity , cps
m/z, Da
83.0869
95.0836
269.2265
313.2634
467.4064
517.4284
569.4563
601.5186
814.6932
814.5228
814.7980
814.7627
814.5766
b
Frequency
Relative content(×10³)
c
- log10 (P)
d
Observed - log10(P)
Expected - log10(P)
e
- log10 (P)
CDH7
GVQW3
Mb
Chromosome 2
CDH7 Expression
f
Breast
Liver
Brain
Fat
Heart
Kidney
Spleen
Skin
Lung
P
g
**
**
L
P
1d
1w
2w
3w
4w
5w
6w

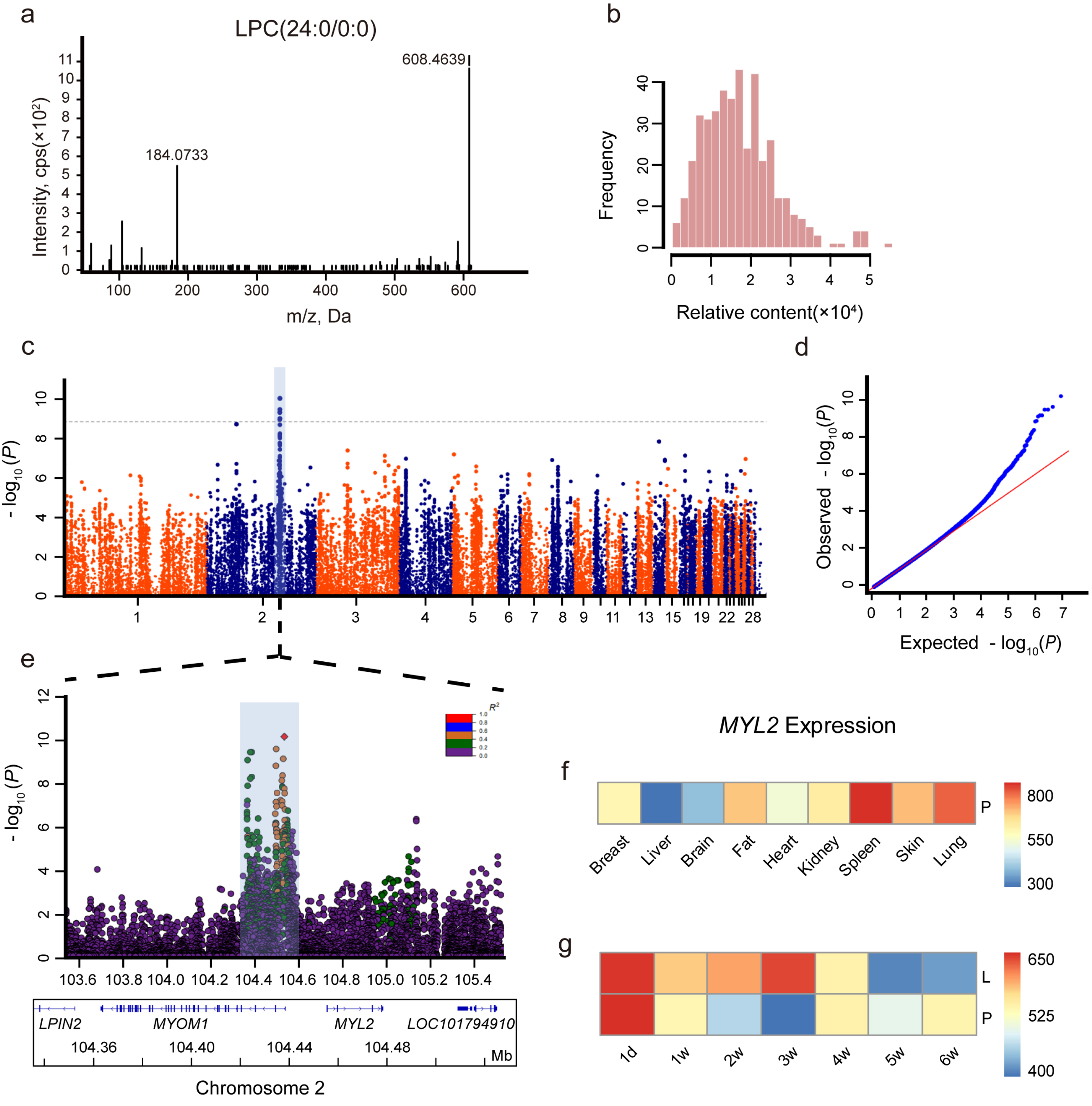
a
LPC(24:0/0:0)
608.4639
184.0733
Intensity, cps(×10²)
m/z, Da
b
Frequency
Relative content(×10⁴)
c
- log10 (P)
d
Observed - log10(P)
Expected - log10(P)
e
- log10 (P)
LPIN2
MYOM1
MYL2
LOC101794910
Mb
Chromosome 2
MYL2 Expression
f
Breast
Liver
Brain
Fat
Heart
Kidney
Spleen
Skin
Lung
P
g
1d
1w
2w
3w
4w
5w
6w
L
P

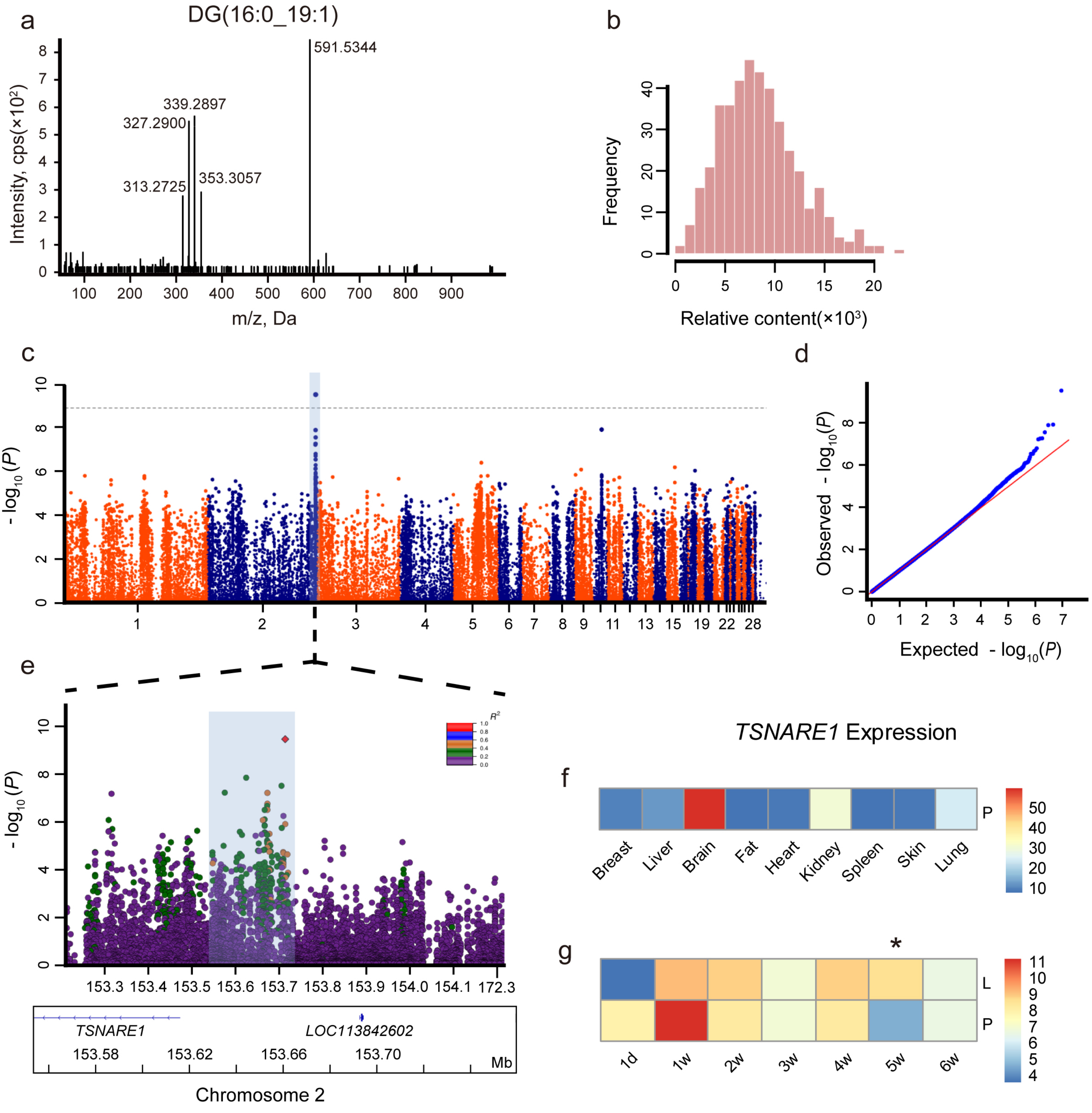

a
DG(16:0_19:1)
591.5344
339.2897
327.2900
313.2725
353.3057
Intensity, cps(×10²)
m/z, Da
b
Frequency
Relative content(×10³)
c
- log10 (P)
d
Observed - log10(P)
Expected - log10(P)
e
- log10 (P)
TSNARE1
LOC113842602
Mb
Chromosome 2
TSNARE1 Expression
f
Breast
Liver
Brain
Fat
Heart
Kidney
Spleen
Skin
Lung
P
g
L
P
1d
1w
2w
3w
4w
5w
6w

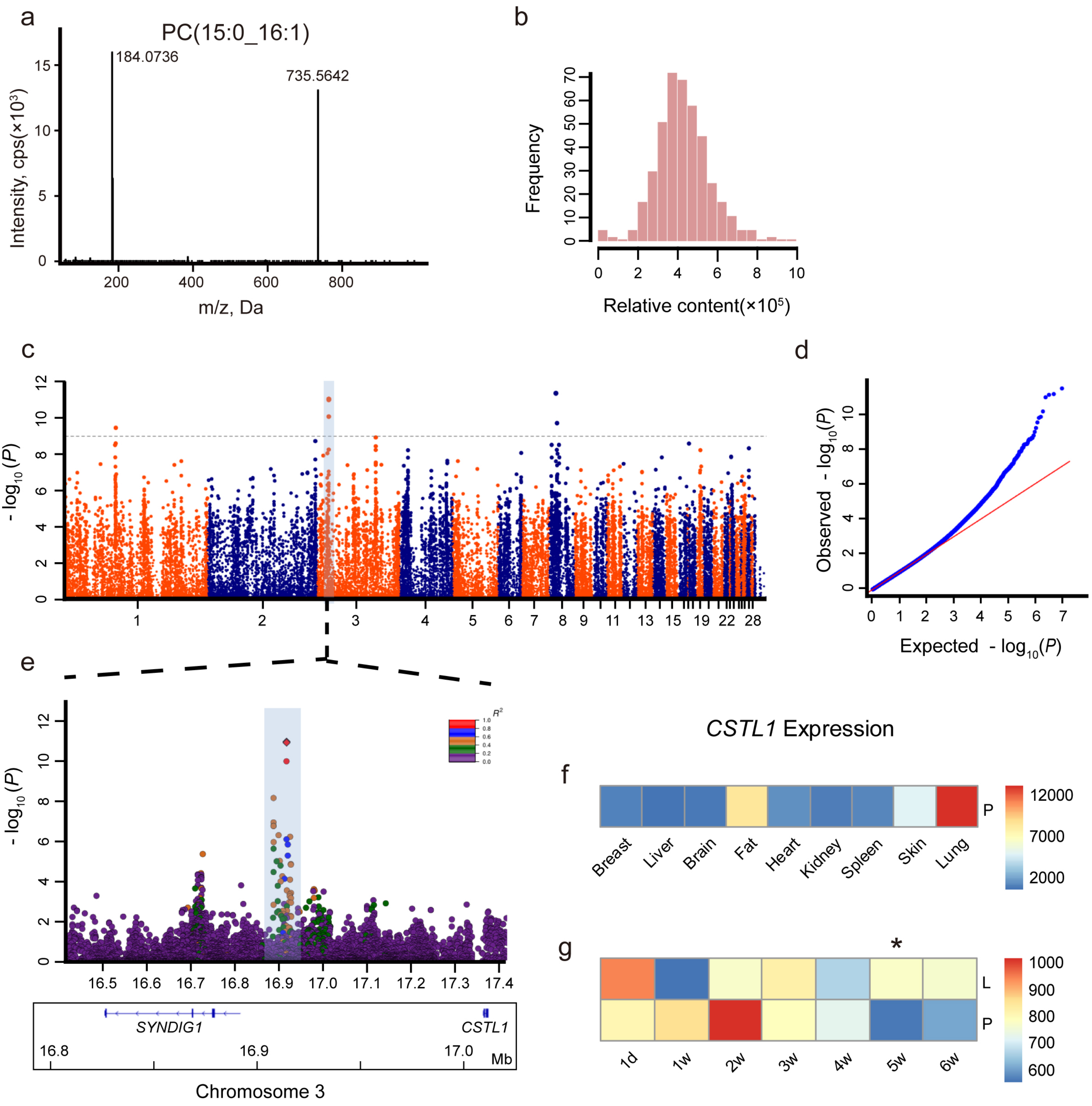

a
PC(15:0_16:1)
184.0736
735.5642
Intensity, cps(×10³)
m/z, Da
b
Frequency
Relative content(×10⁵)
c
- log10 (P)
d
Observed - log10(P)
Expected - log10(P)
e
- log10(P)
SYNDIG1
CSTL1
Chromosome 3
Mb
CSTL1 Expression
f
Breast
Liver
Brain
Fat
Heart
Kidney
Spleen
Skin
Lung
P
12000
7000
2000
g
L
P
1d
1w
2w
3w
4w
5w
6w
1000
900
800
700
600

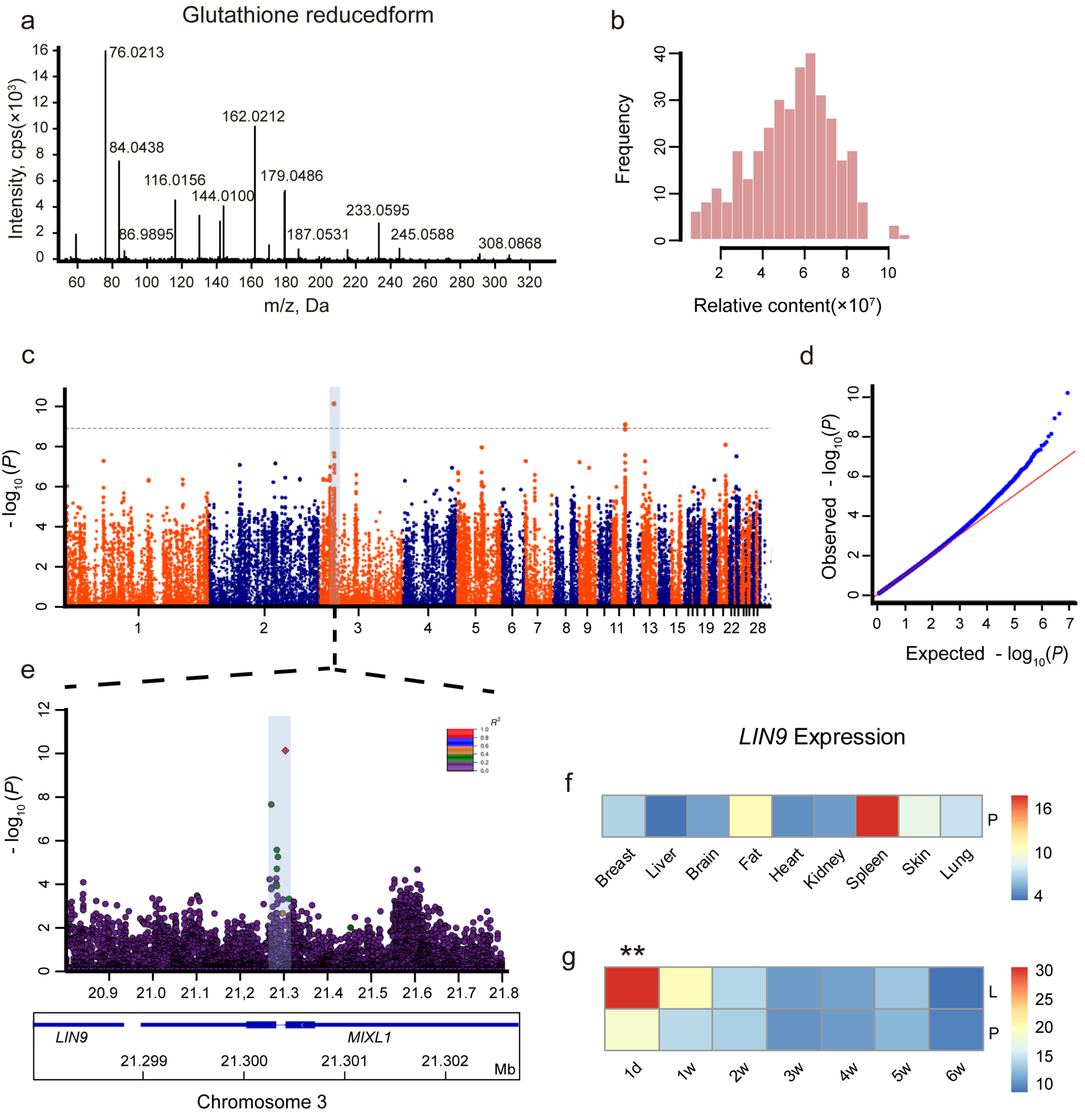
a
Glutathione reducedform
Intensity, cps(×10³)
76.0213
84.0438
86.9895
116.0156
144.0100
162.0212
179.0486
187.0531
233.0595
245.0588
308.0868
m/z, Da
b
Frequency
Relative content(×10⁷)
c
- log10(P)
d
Observed - log10(P)
Expected - log10(P)
e
- log10(P)
LIN9
MIXL1
Mb
Chromosome 3
LIN9 Expression
f
Breast
Liver
Brain
Fat
Heart
Kidney
Spleen
Skin
Lung
P
g
**
L
P
1d
1w
2w
3w
4w
5w
6w

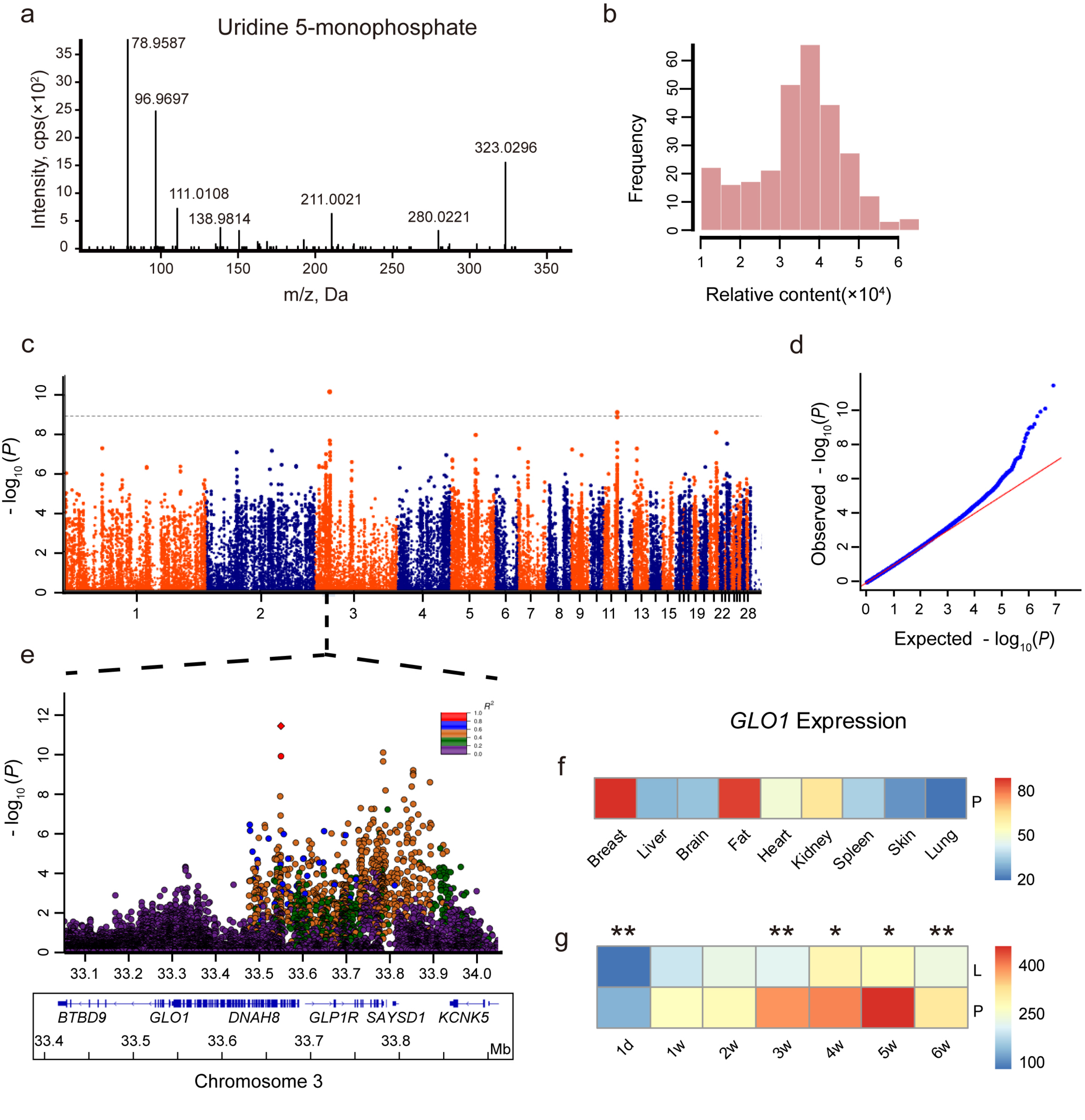

a
Uridine 5-monophosphate
78.9587
96.9697
111.0108
138.9814
211.0021
280.0221
323.0296
Intensity, cps(×10²)
m/z, Da
b
Frequency
Relative content(×10⁴)
c
- log10(P)
d
Observed - log10(P)
Expected - log10(P)
e
- log10(P)
BTBD9
GLO1
DNAH8
GLP1R
SAYSD1
KCNK5
Mb
Chromosome 3
GLO1 Expression
f
Breast
Liver
Brain
Fat
Heart
Kidney
Spleen
Skin
Lung
P
g
L
P
1d
1w
2w
3w
4w
5w
6w

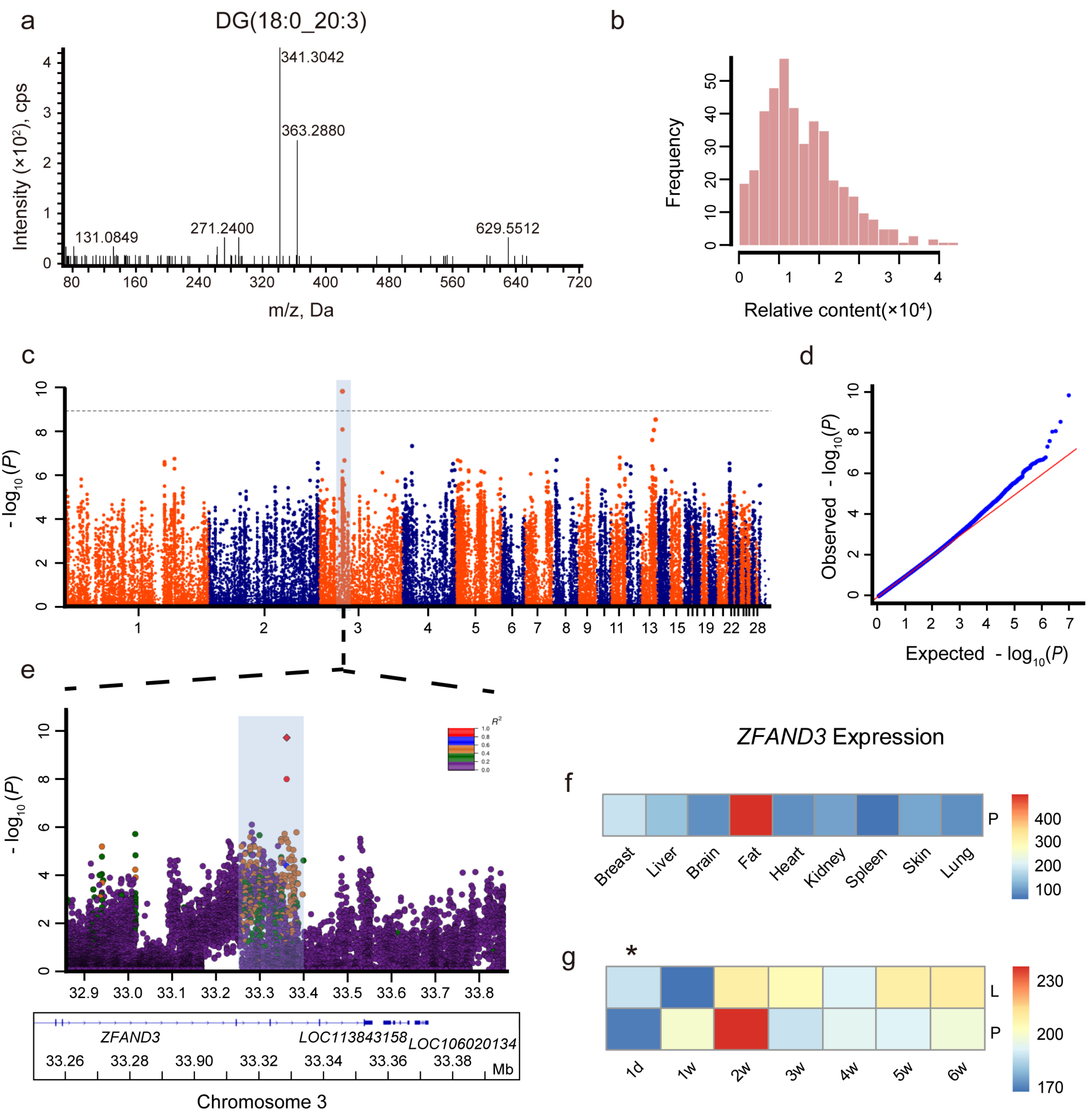
a
DG(18:0_20:3)
Intensity (×10²), cps
341.3042
363.2880
131.0849
271.2400
629.5512
m/z, Da
b
Frequency
Relative content(×10⁴)
c
- log10(P)
d
Observed - log10(P)
Expected - log10(P)
e
- log10(P)
R²
ZFAND3
LOC113843158
LOC106020134
Mb
Chromosome 3
ZFAND3 Expression
f
Breast
Liver
Brain
Fat
Heart
Kidney
Spleen
Skin
Lung
P
g
*
L
P
1d
1w
2w
3w
4w
5w
6w

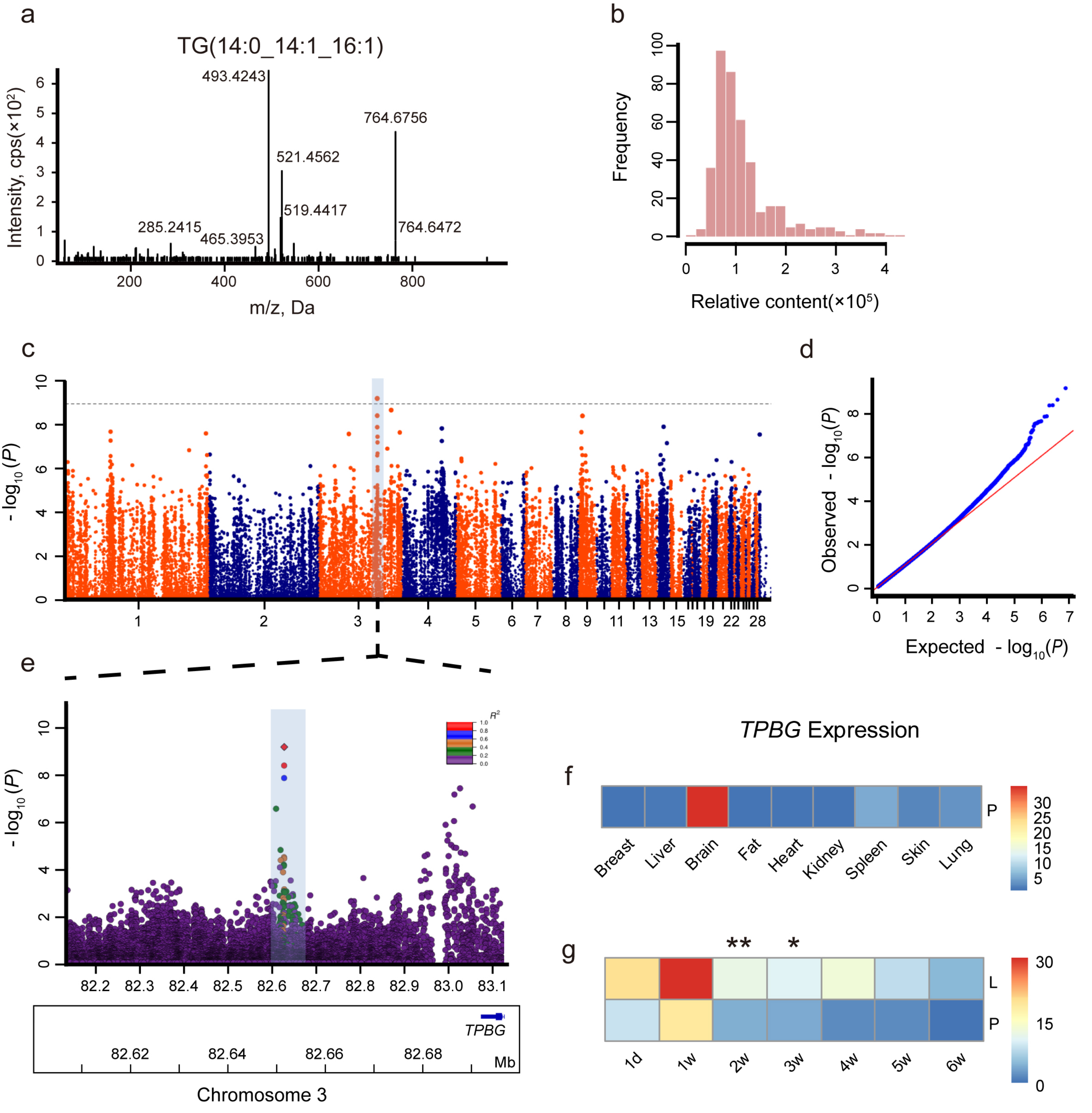
a
TG(14:0_14:1_16:1)
493.4243
764.6756
521.4562
519.4417
285.2415
465.3953
764.6472
Intensity, cps(×10²)
m/z, Da
b
Frequency
Relative content(×10⁵)
c
- log10(P)
d
Observed - log10(P)
Expected - log10(P)
e
- log10(P)
TPBG
82.62
82.64
82.66
82.68
Mb
Chromosome 3
TPBG Expression
f
Breast
Liver
Brain
Fat
Heart
Kidney
Spleen
Skin
Lung
P
g
**
*
L
P
1d
1w
2w
3w
4w
5w
6w

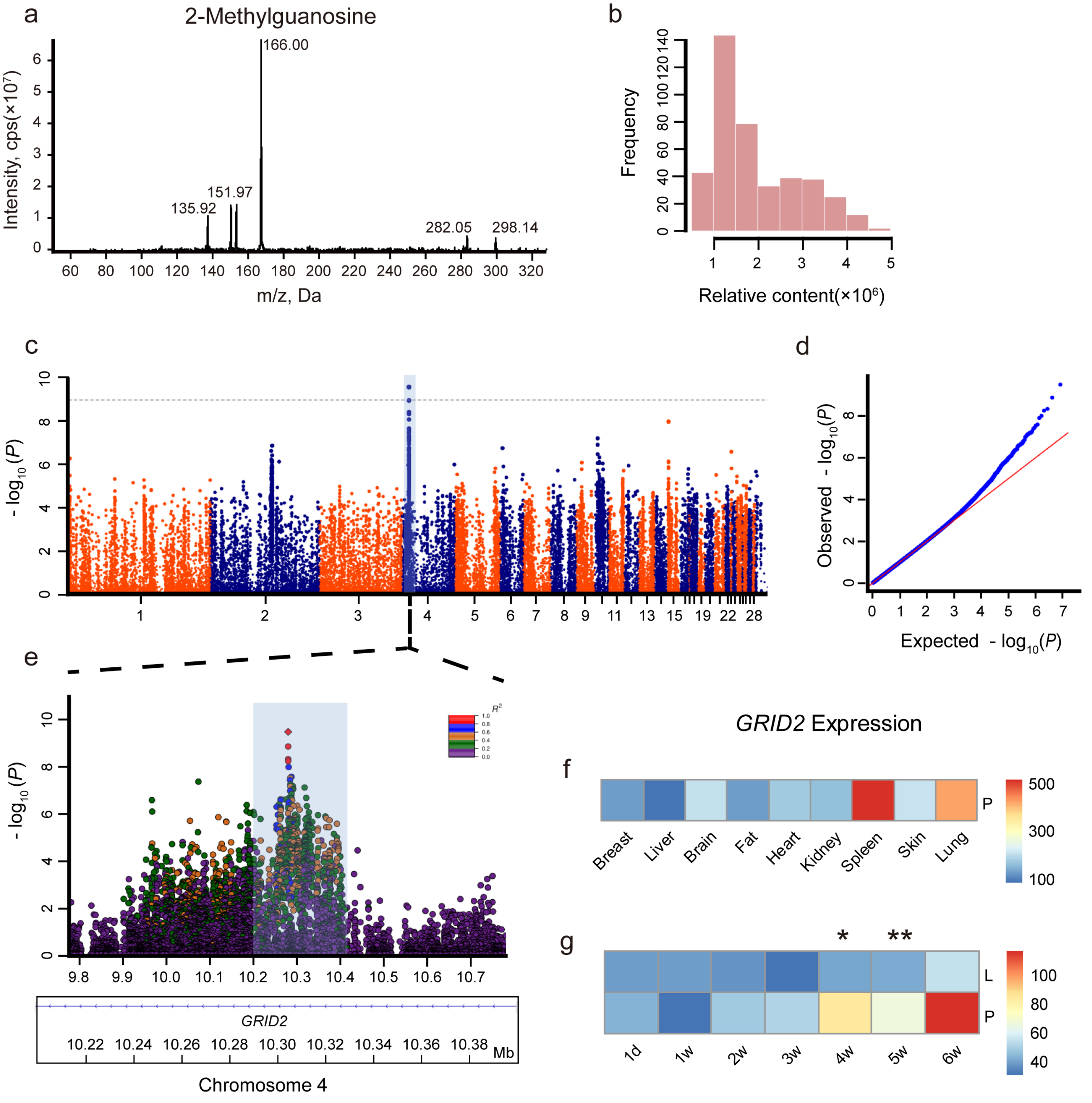

a
2-Methylguanosine
Intensity, cps(×10⁷)
166.00
151.97
135.92
282.05
298.14
m/z, Da
b
Frequency
Relative content(×10⁶)
c
- log10(P)
d
Observed - log10(P)
Expected - log10(P)
e
- log10(P)
GRID2
Mb
Chromosome 4
GRID2 Expression
f
Breast
Liver
Brain
Fat
Heart
Kidney
Spleen
Skin
Lung
P
g
L
P
1d
1w
2w
3w
4w
5w
6w
*
**
