# Supplementary material for: Metabolome‐Based Genome‐Wide Association Study of Duck Meat Leads to Novel Genetic and Biochemical Insights
Source: Adv Sci (Weinh). 2023 Apr 4;10(18):2300148. doi: 10.1002/advs.202300148 (PMC10288243; doi:10.1002/advs.202300148)
Supplement: Supplementary file 3 — Supporting Information [file ADVS-10-2300148-s001.pdf]

ADVANCED SCIENCE
Open Access

Supporting Information

for *Adv. Sci.*, DOI 10.1002/advs.202300148

Metabolome-Based Genome-Wide Association Study of Duck Meat Leads to Novel Genetic and Biochemical Insights

Dapeng Liu, He Zhang, Youyou Yang, Tong Liu, Zhanbao Guo, Wenlei Fan, Zhen Wang, Xinting Yang, Bo Zhang, Hongfei Liu, Hehe Tang, Daxin Yu, Simeng Yu, Kai Gai, Qiming Mou, Junting Cao, Jian Hu, Jing Tang, Shuisheng Hou and Zhengkui Zhou**

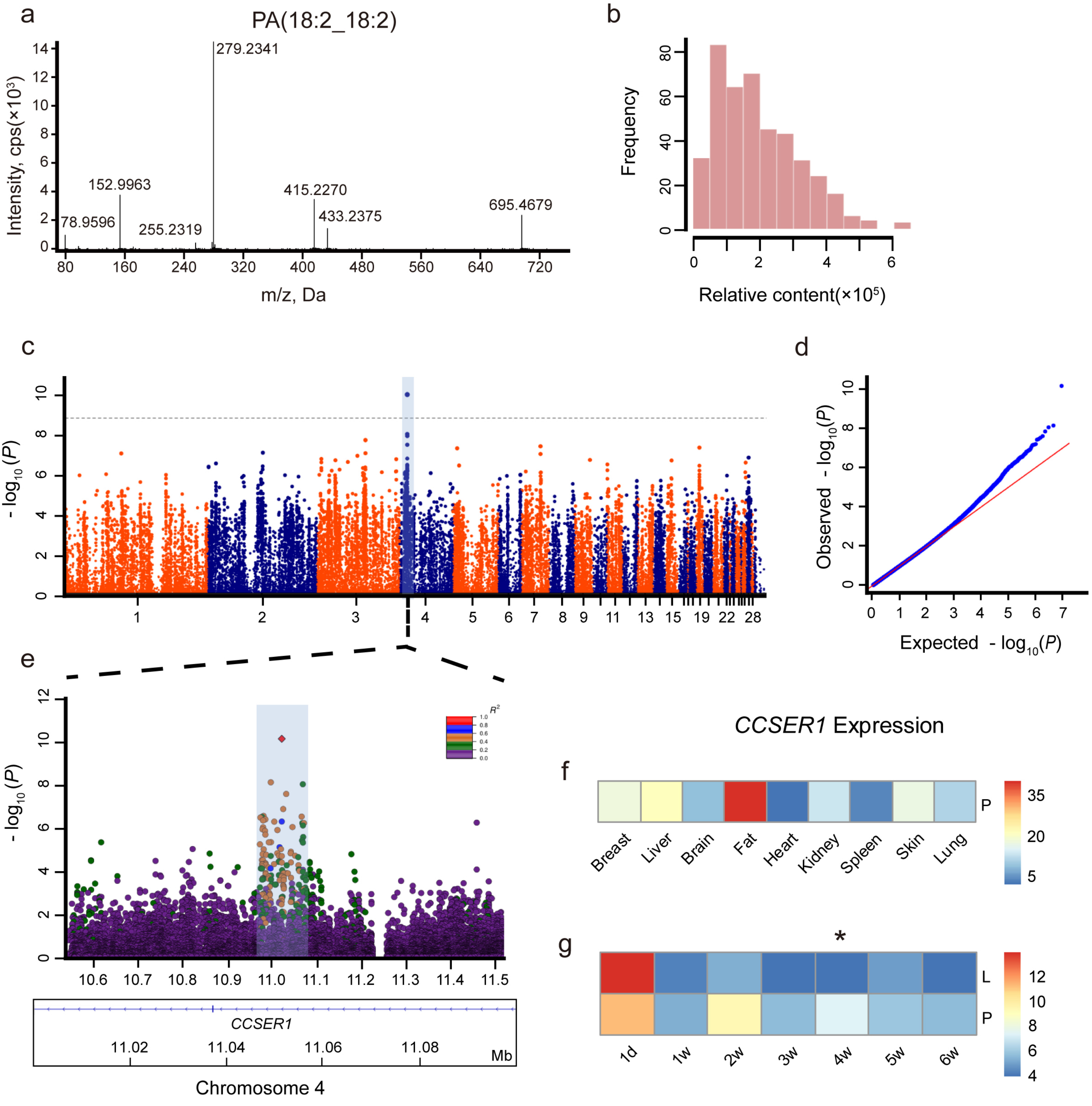
a
PA(18:2_18:2)
Intensity, cps(×10³)
m/z, Da
279.2341
152.9963
78.9596
255.2319
415.2270
433.2375
695.4679
b
Frequency
Relative content(×10⁵)
c
- log10 (P)
d
Observed - log10(P)
Expected - log10(P)
e
R²
CCSER1
Mb
Chromosome 4
CCSER1 Expression
f
Breast
Liver
Brain
Fat
Heart
Kidney
Spleen
Skin
Lung
P
g
*
L
P
1d
1w
2w
3w
4w
5w
6w

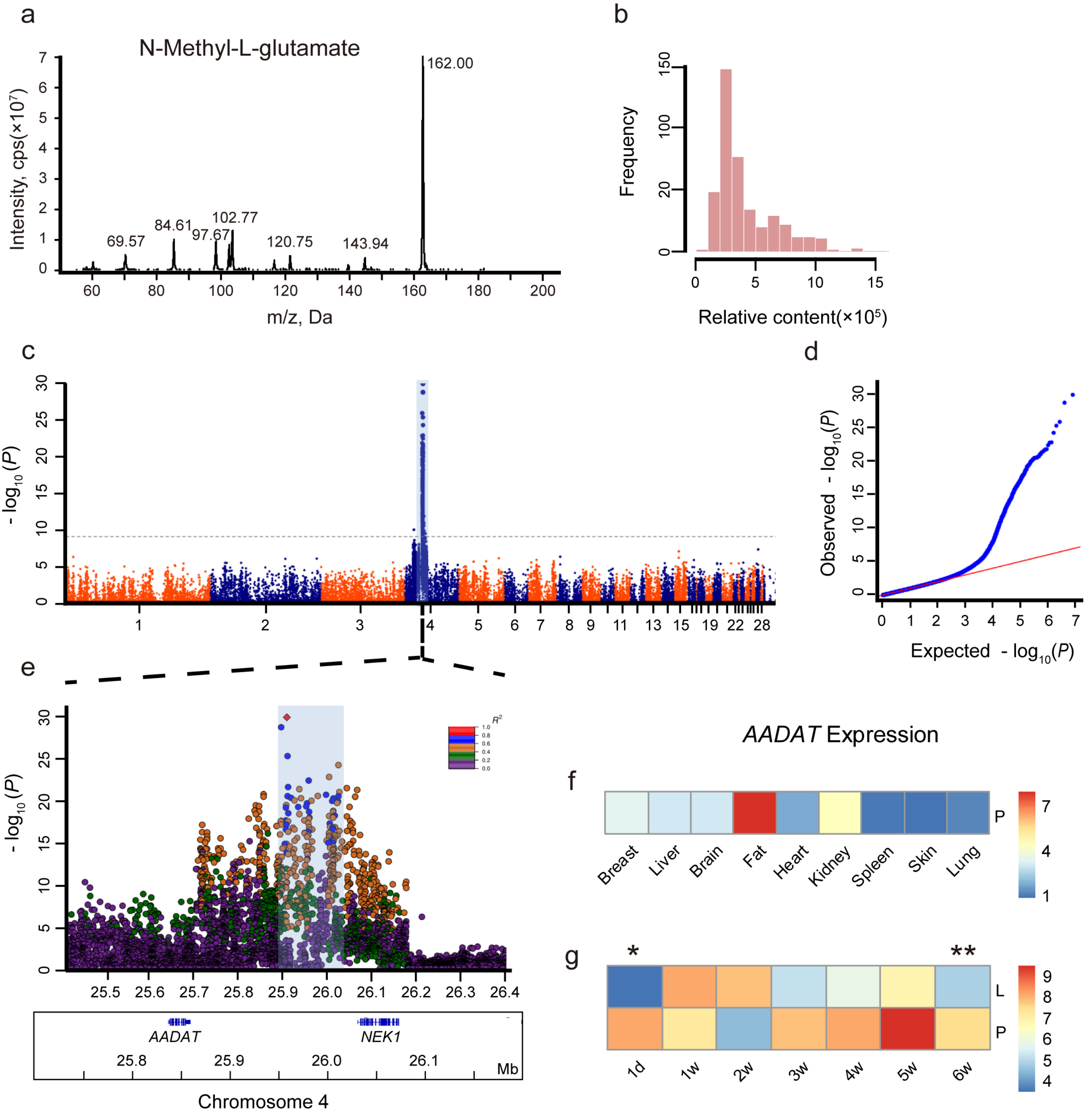

a
N-Methyl-L-glutamate
Intensity, cps(×10^7)
m/z, Da
162.00
69.57
84.61
97.67
102.77
120.75
143.94
b
Frequency
Relative content(×10^5)
c
- log10 (P)
d
Observed - log10(P)
Expected - log10(P)
e
- log10 (P)
AADAT
NEK1
Mb
Chromosome 4
AADAT Expression
f
Breast
Liver
Brain
Fat
Heart
Kidney
Spleen
Skin
Lung
P
g
*
**
L
P
1d
1w
2w
3w
4w
5w
6w

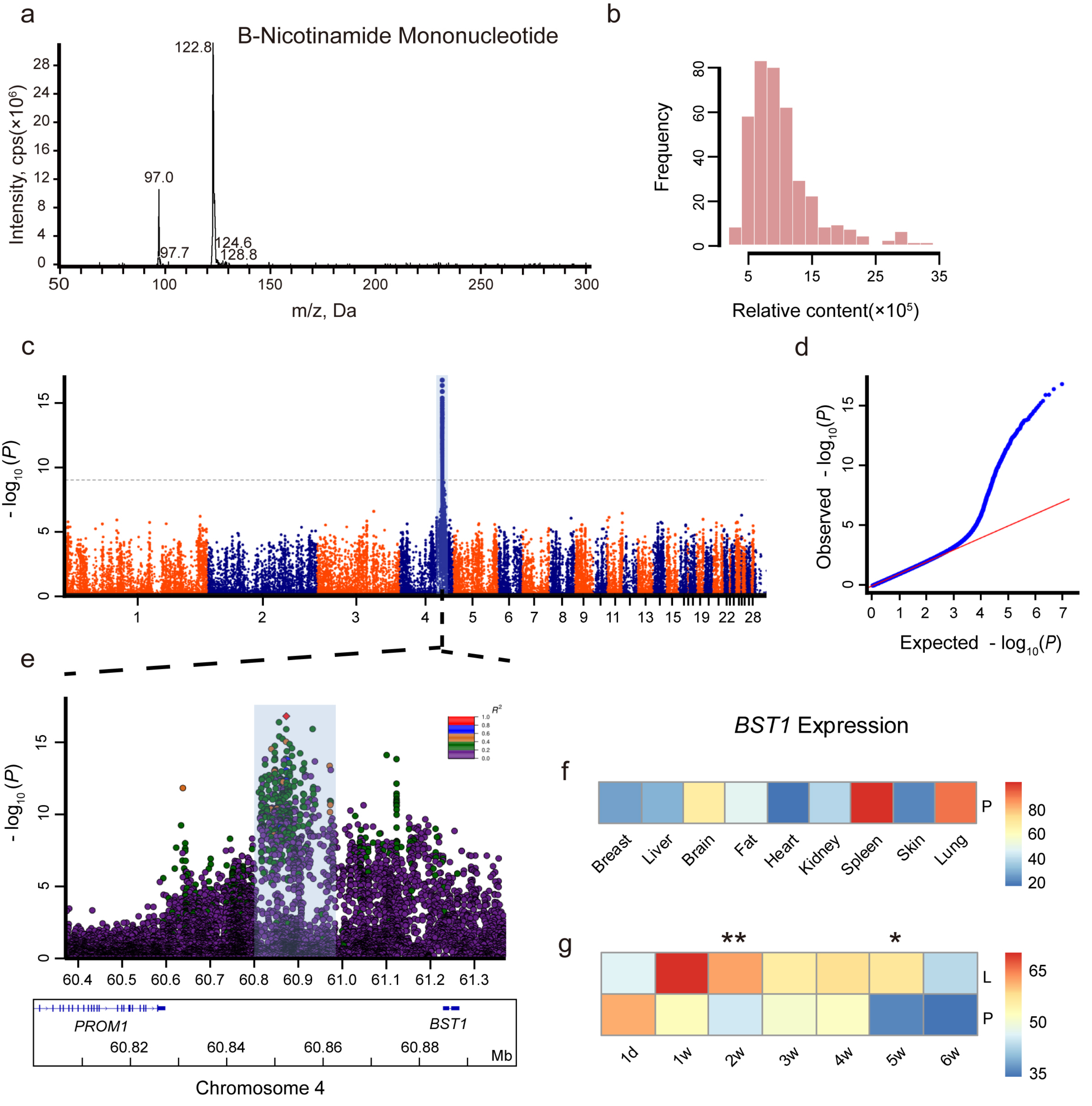

a
B-Nicotinamide Mononucleotide
Intensity, cps(×10⁶)
m/z, Da
122.8
97.0
97.7
124.6
128.8
b
Frequency
Relative content(×10⁵)
c
- log10 (P)
d
Observed - log10(P)
Expected - log10(P)
e
- log10 (P)
PROM1
BST1
Mb
Chromosome 4
BST1 Expression
f
Breast
Liver
Brain
Fat
Heart
Kidney
Spleen
Skin
Lung
P
g
**
*
L
P
1d
1w
2w
3w
4w
5w
6w

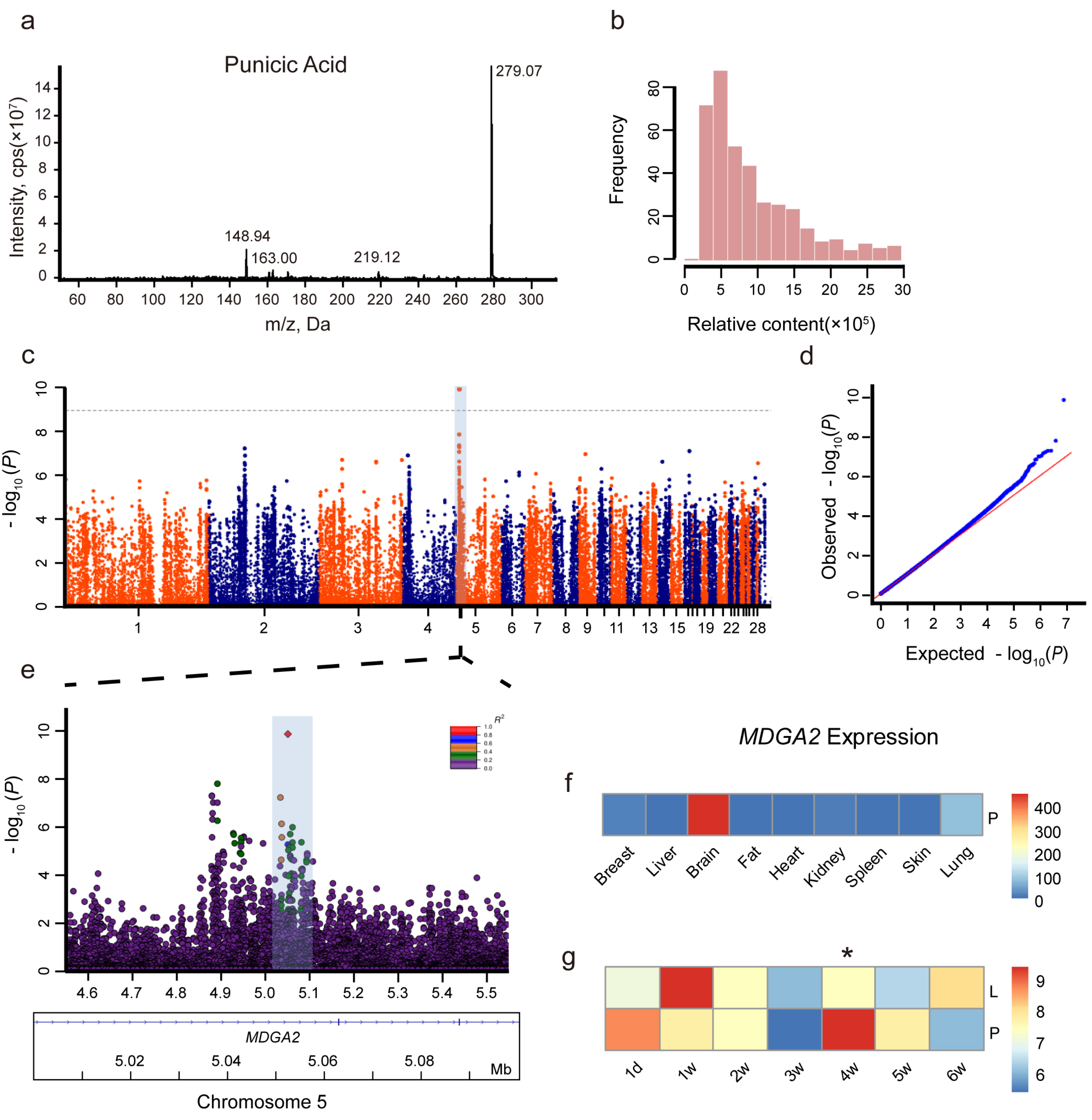

a
Punicic Acid
Intensity, cps(×10^7)
279.07
148.94
163.00
219.12
m/z, Da
b
Frequency
Relative content(×10^5)
c
- log10(P)
d
Observed - log10(P)
Expected - log10(P)
e
- log10(P)
R^2
MDGA2
Mb
Chromosome 5
MDGA2 Expression
f
Breast
Liver
Brain
Fat
Heart
Kidney
Spleen
Skin
Lung
P
g
*
L
P
1d
1w
2w
3w
4w
5w
6w

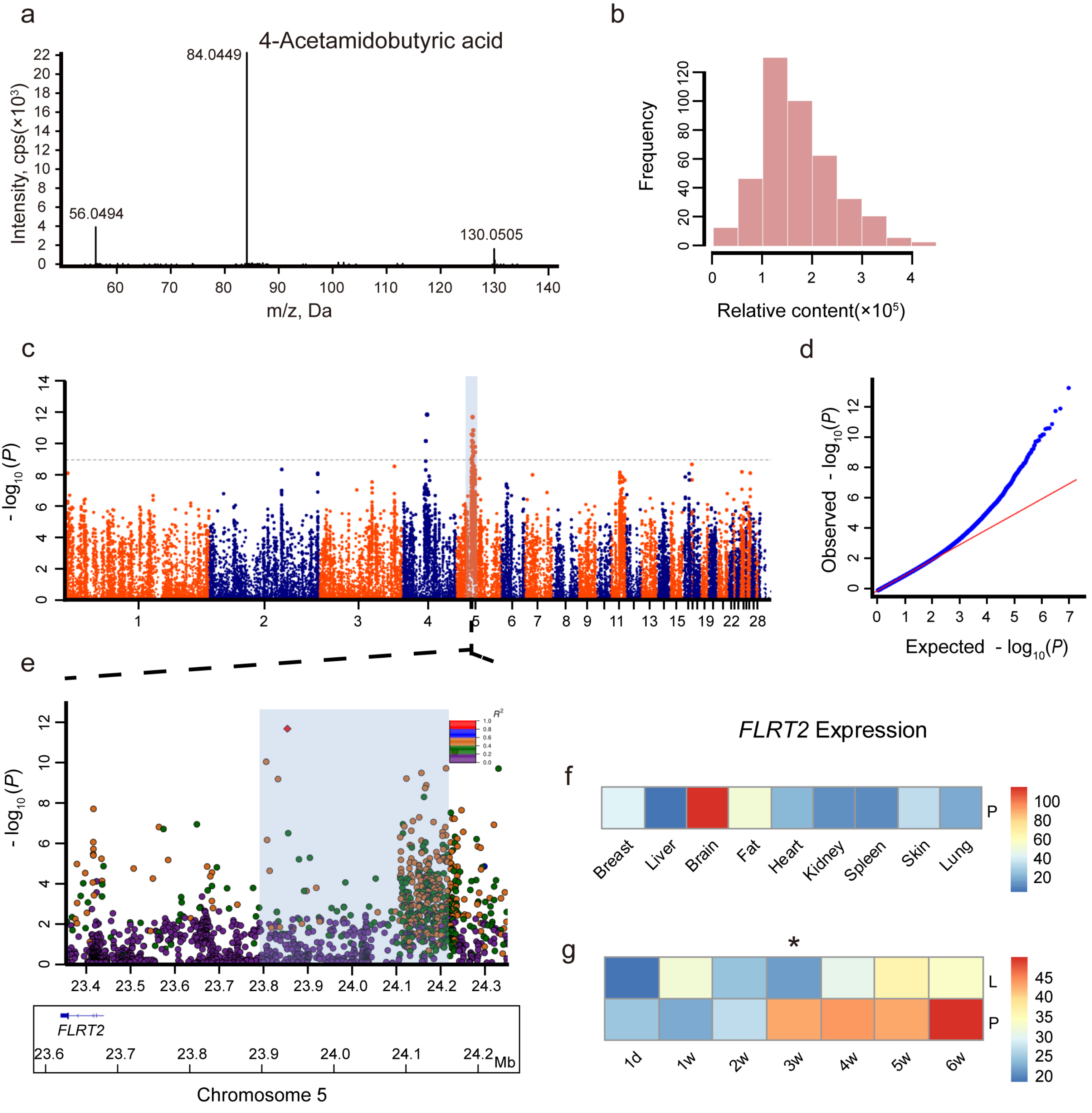
a
4-Acetamidobutyric acid
Intensity, cps(×10³)
84.0449
56.0494
130.0505
m/z, Da
b
Frequency
Relative content(×10⁵)
c
- log10 (P)
d
Observed - log10(P)
Expected - log10(P)
e
- log10 (P)
R²
FLRT2
Chromosome 5
Mb
FLRT2 Expression
f
Breast
Liver
Brain
Fat
Heart
Kidney
Spleen
Skin
Lung
P
g
*
L
P
1d
1w
2w
3w
4w
5w
6w

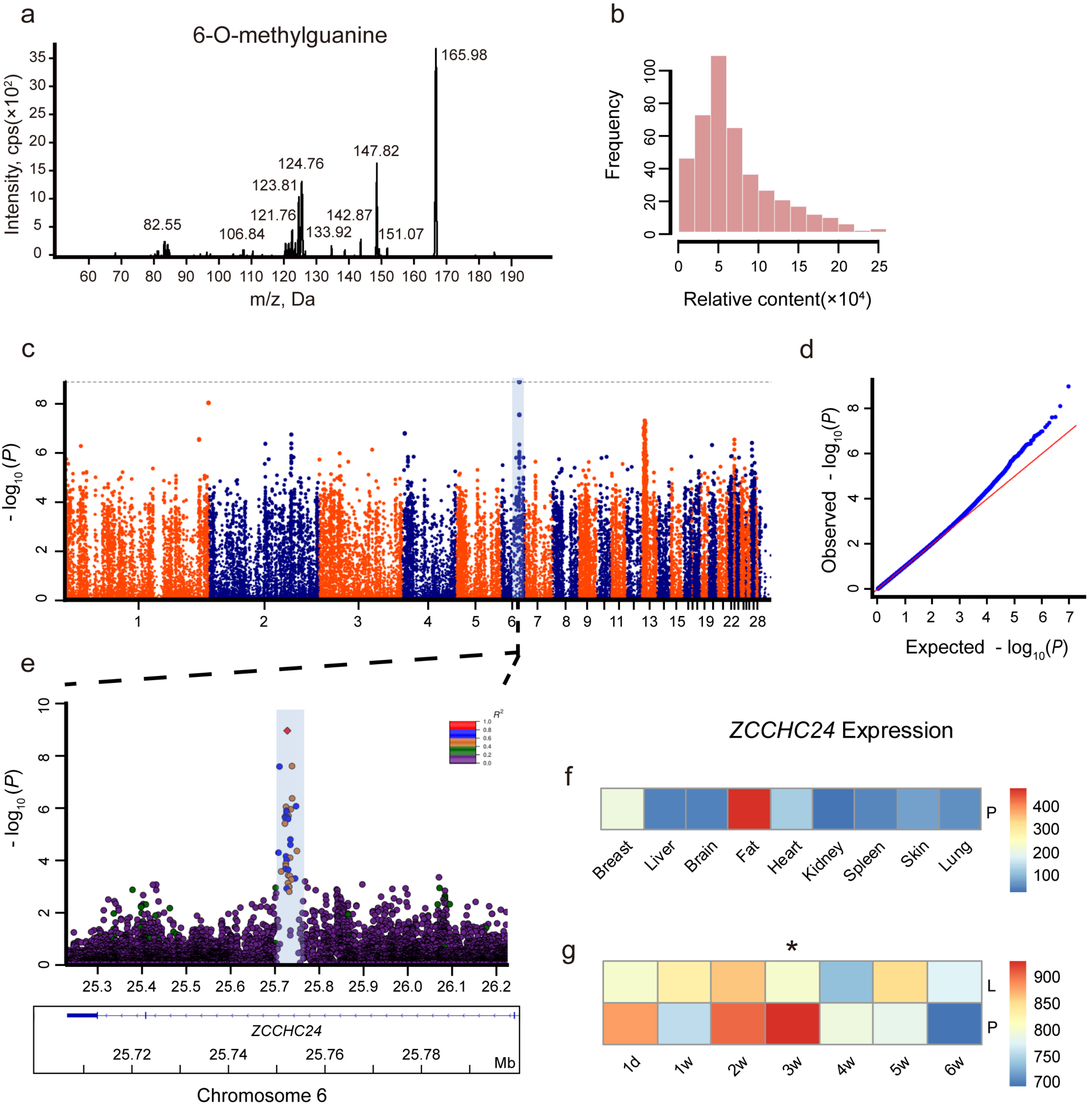
a
6-O-methylguanine
Intensity, cps(×10²)
m/z, Da
165.98
147.82
124.76
123.81
121.76
142.87
133.92
151.07
82.55
106.84
b
Frequency
Relative content(×10⁴)
c
- log10(P)
d
Observed - log10(P)
Expected - log10(P)
e
- log10(P)
ZCCHC24
Chromosome 6
Mb
ZCCHC24 Expression
f
Breast
Liver
Brain
Fat
Heart
Kidney
Spleen
Skin
Lung
P
g
*
L
P
1d
1w
2w
3w
4w
5w
6w

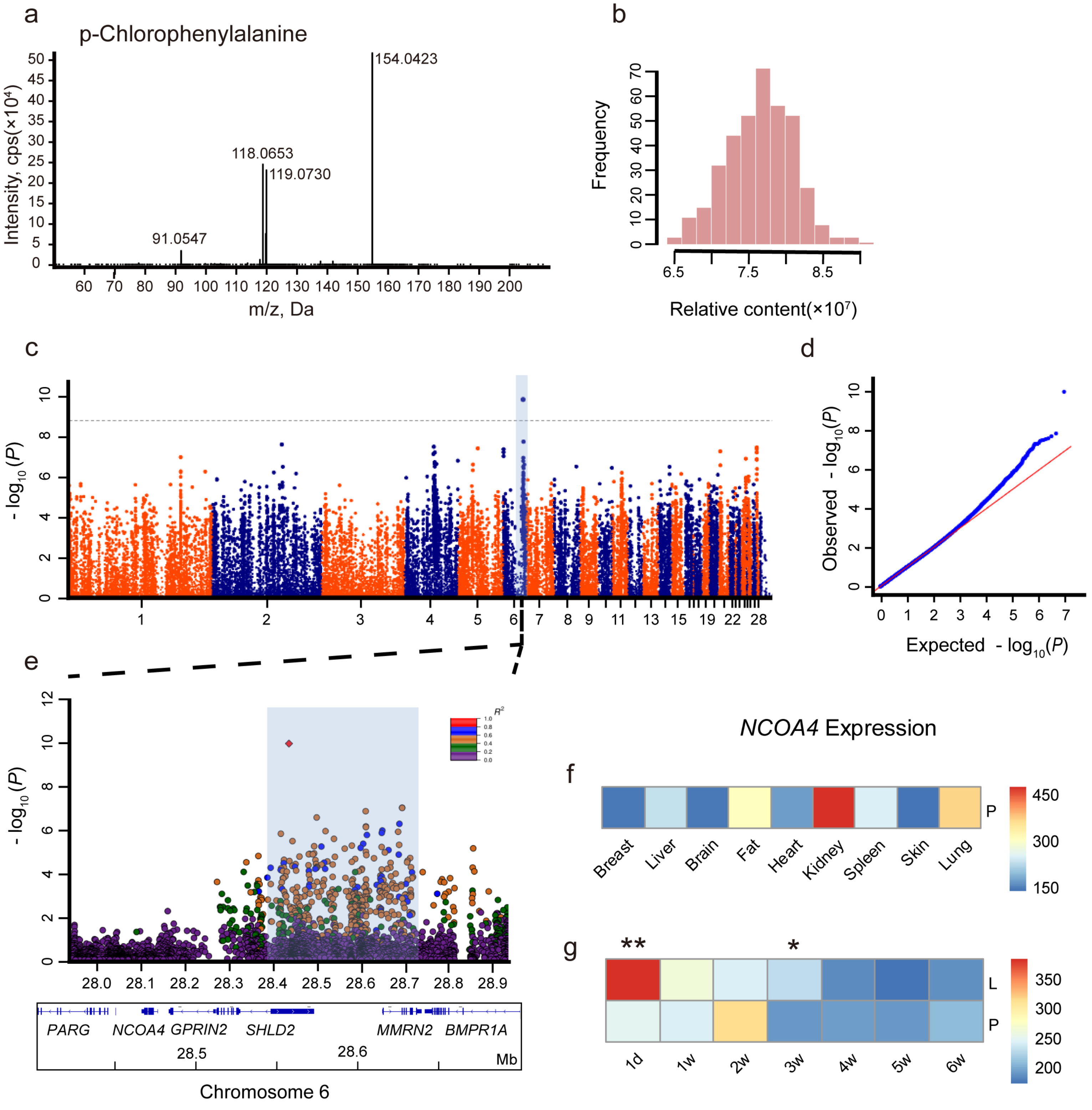

a
p-Chlorophenylalanine
Intensity, cps(×10⁴)
154.0423
118.0653
119.0730
91.0547
m/z, Da
b
Frequency
Relative content(×10⁷)
c
- log10(P)
d
Observed - log10(P)
Expected - log10(P)
e
- log10(P)
PARG
NCOA4
GPRIN2
SHLD2
MMRN2
BMPR1A
Mb
Chromosome 6
NCOA4 Expression
f
Breast
Liver
Brain
Fat
Heart
Kidney
Spleen
Skin
Lung
P
g
L
P
1d
1w
2w
3w
4w
5w
6w

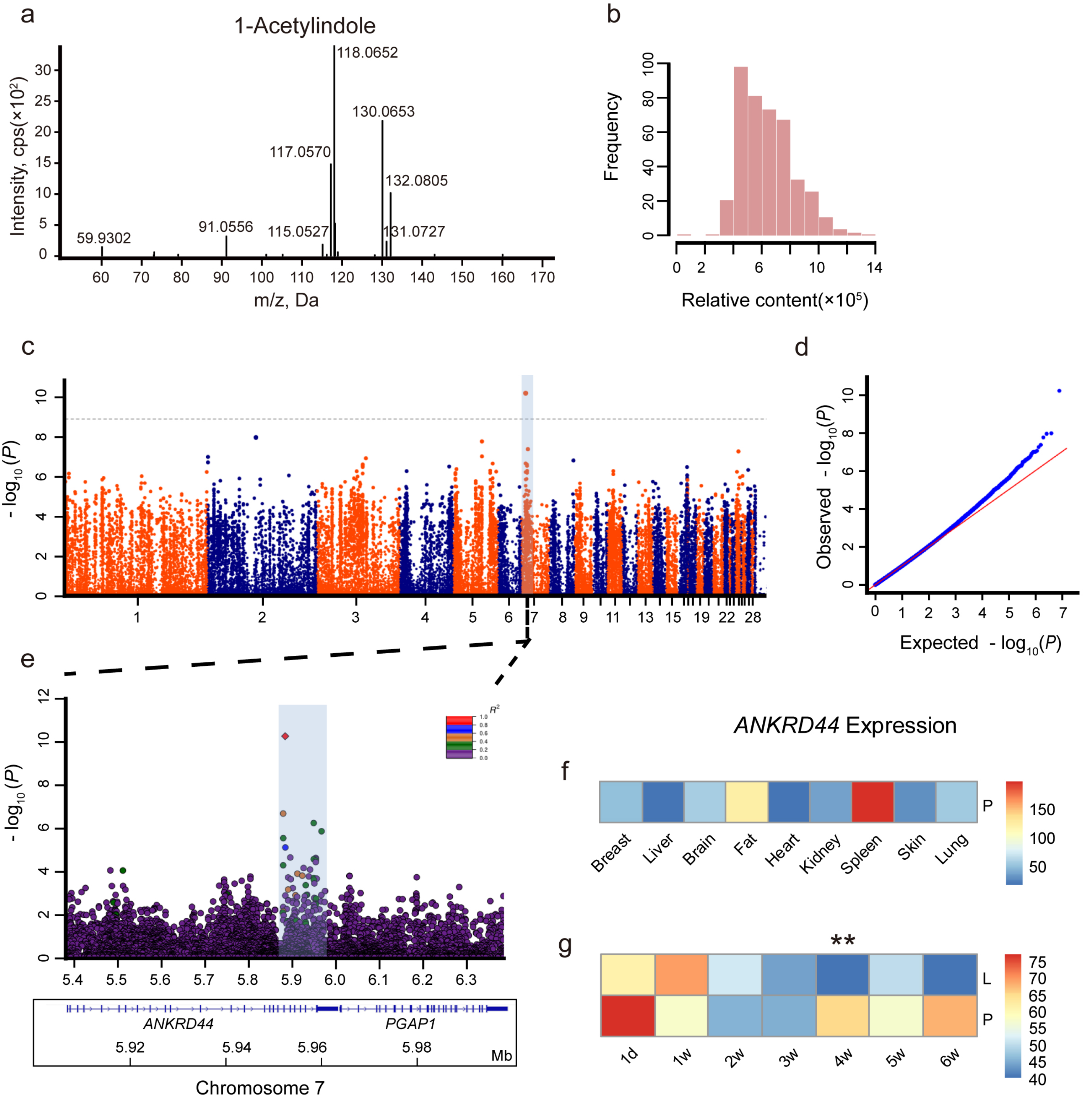
a
1-Acetylindole
Intensity, cps(×10²)
118.0652
130.0653
117.0570
132.0805
59.9302
91.0556
115.0527
131.0727
m/z, Da
b
Frequency
Relative content(×10⁵)
c
- log10(P)
d
Observed - log10(P)
Expected - log10(P)
e
R²
ANKRD44
PGAP1
Mb
Chromosome 7
ANKRD44 Expression
f
Breast
Liver
Brain
Fat
Heart
Kidney
Spleen
Skin
Lung
P
g
**
L
P
1d
1w
2w
3w
4w
5w
6w

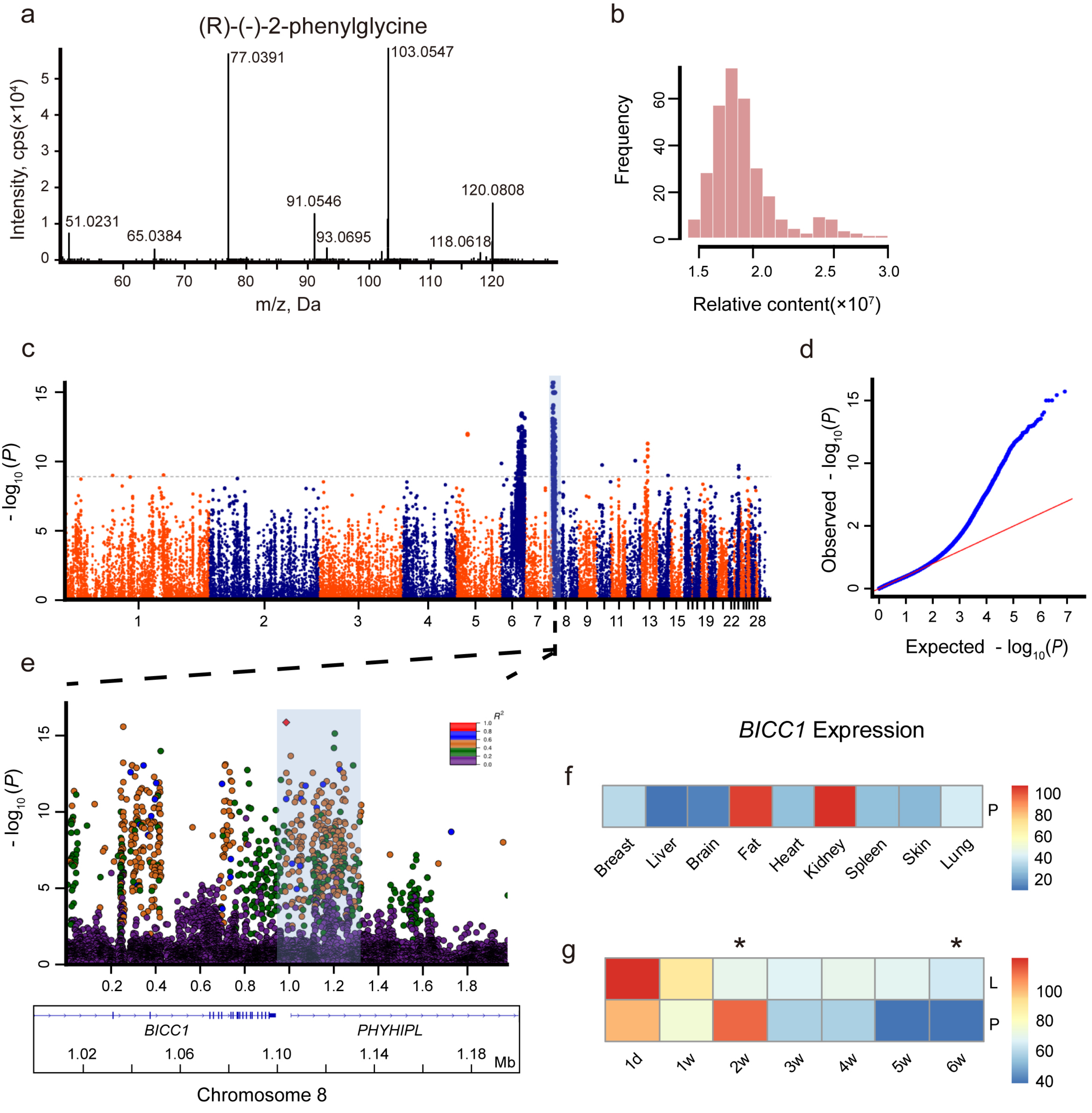

a
(R)-(-)-2-phenylglycine
Intensity, cps(×10⁴)
m/z, Da
51.0231
65.0384
77.0391
91.0546
93.0695
103.0547
118.0618
120.0808
b
Frequency
Relative content(×10⁷)
c
- log10(P)
d
Observed - log10(P)
Expected - log10(P)
e
- log10(P)
R²
BICC1
PHYHIPL
Mb
Chromosome 8
BICC1 Expression
f
Breast
Liver
Brain
Fat
Heart
Kidney
Spleen
Skin
Lung
P
g
L
P
1d
1w
2w
3w
4w
5w
6w

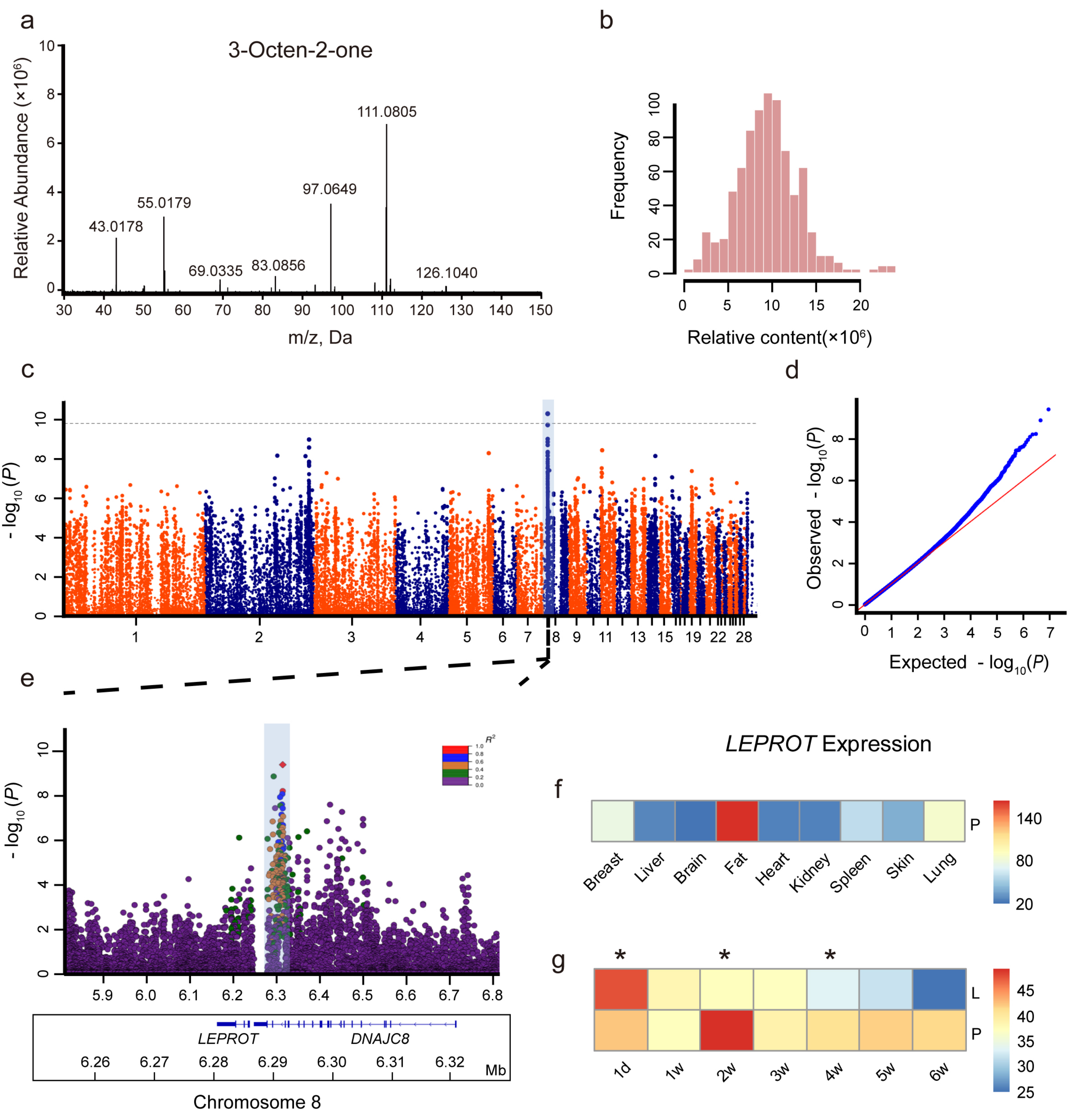
a
3-Octen-2-one
Relative Abundance (×10⁶)
43.0178
55.0179
69.0335
83.0856
97.0649
111.0805
126.1040
m/z, Da
b
Frequency
Relative content(×10⁶)
c
- log10(P)
d
Observed - log10(P)
Expected - log10(P)
e
- log10(P)
LEPROT
DNAJC8
Mb
Chromosome 8
LEPROT Expression
f
Breast
Liver
Brain
Fat
Heart
Kidney
Spleen
Skin
Lung
P
g
L
P
1d
1w
2w
3w
4w
5w
6w

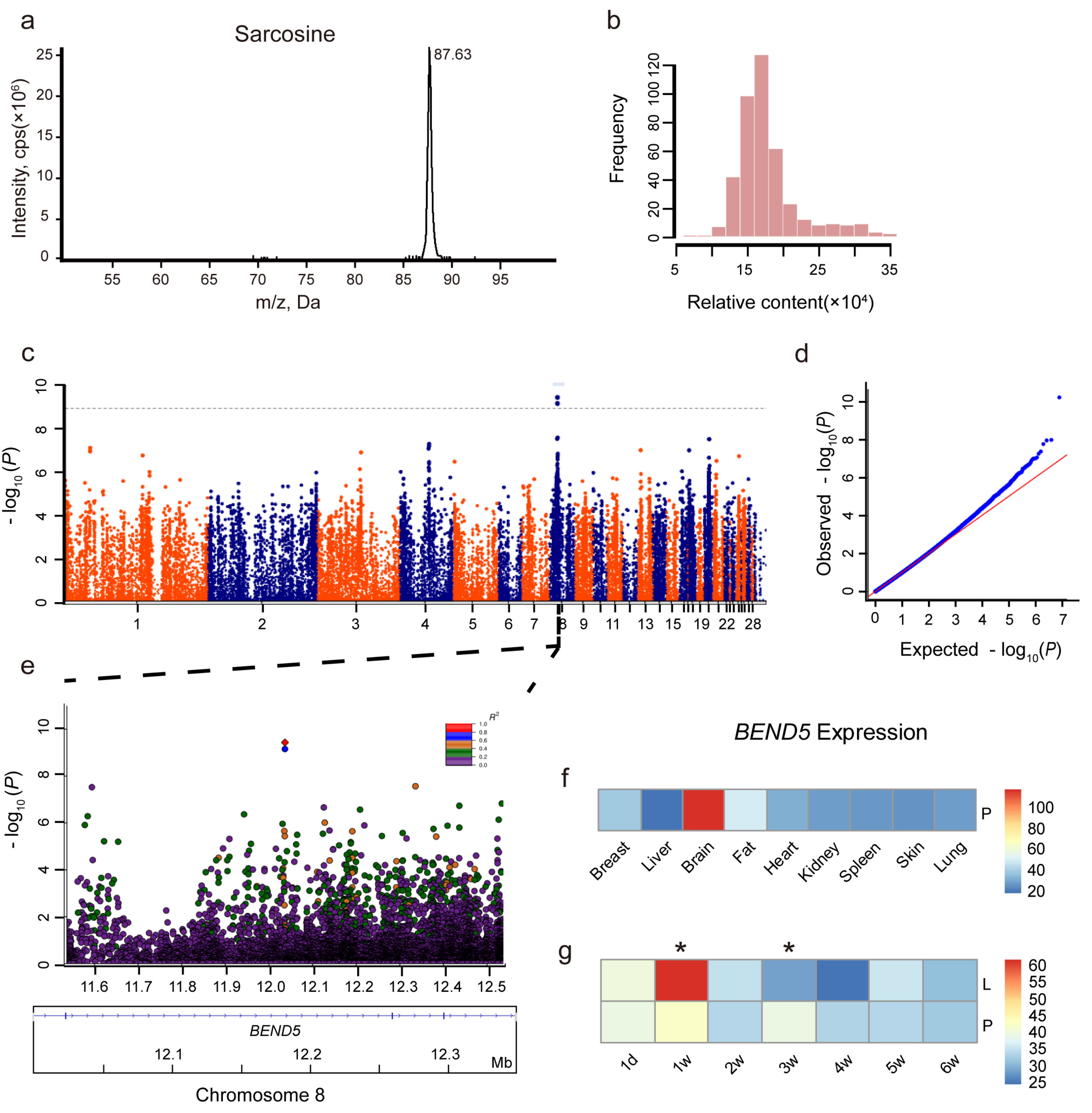
a
Sarcosine
87.63
Intensity, cps(×10⁶)
m/z, Da
b
Frequency
Relative content(×10⁴)
c
- log10(P)
d
Observed - log10(P)
Expected - log10(P)
e
- log10(P)
R²
BEND5
Chromosome 8
Mb
BEND5 Expression
f
Breast
Liver
Brain
Fat
Heart
Kidney
Spleen
Skin
Lung
P
g
L
P
1d
1w
2w
3w
4w
5w
6w

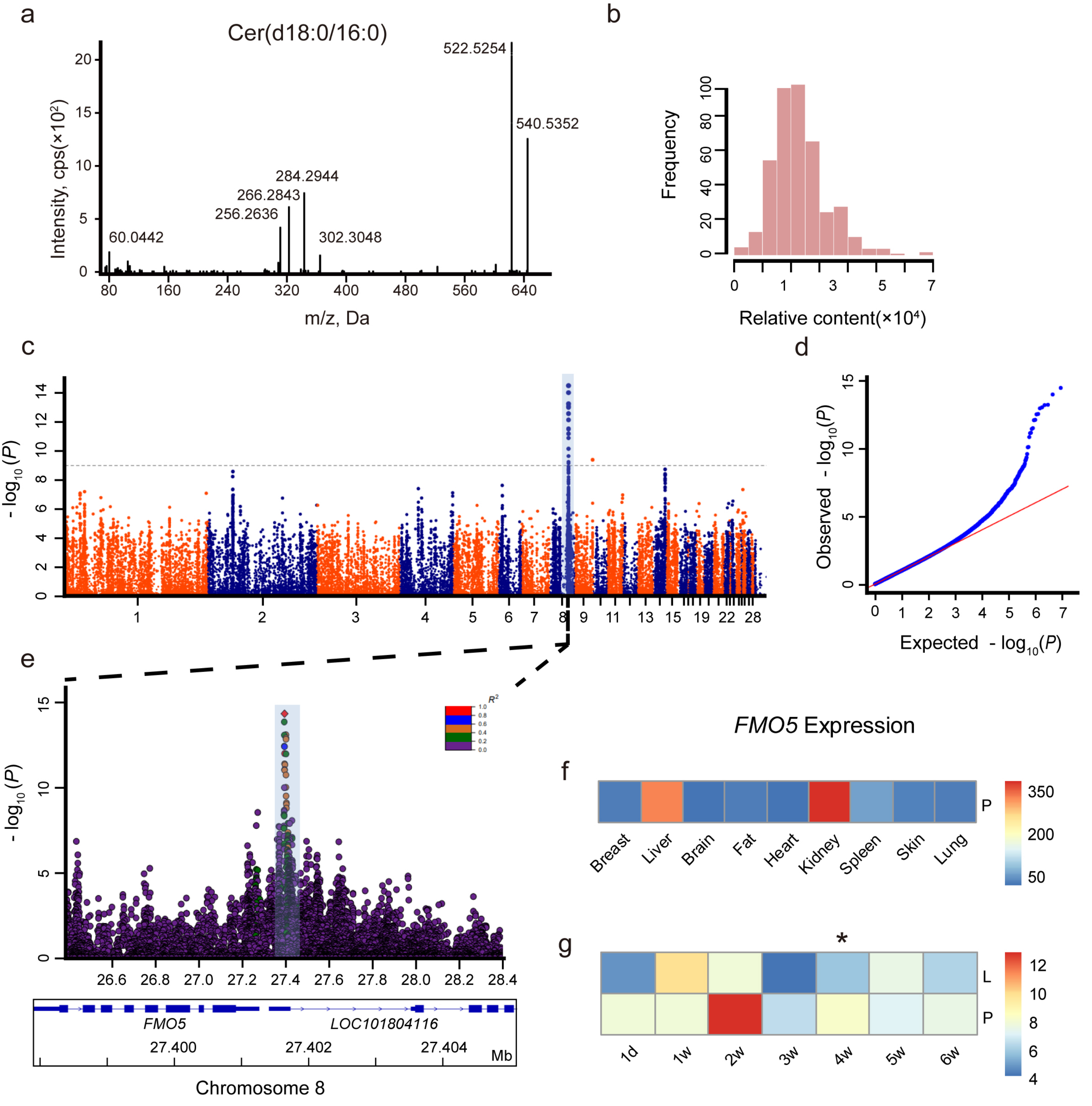

a
Cer(d18:0/16:0)
Intensity, cps(×10²)
m/z, Da
522.5254
540.5352
284.2944
266.2843
256.2636
302.3048
60.0442
b
Frequency
Relative content(×10⁴)
c
- log10(P)
d
Observed - log10(P)
Expected - log10(P)
e
- log10(P)
FMO5
LOC101804116
Mb
Chromosome 8
FMO5 Expression
f
Breast
Liver
Brain
Fat
Heart
Kidney
Spleen
Skin
Lung
P
g
L
P
1d
1w
2w
3w
4w
5w
6w
