# Supplementary material for: Metabolome‐Based Genome‐Wide Association Study of Duck Meat Leads to Novel Genetic and Biochemical Insights
Source: Adv Sci (Weinh). 2023 Apr 4;10(18):2300148. doi: 10.1002/advs.202300148 (PMC10288243; doi:10.1002/advs.202300148)
Supplement: Supplementary file 4 — Supporting Information [file ADVS-10-2300148-s002.pdf]

ADVANCED SCIENCE
Open Access

Supporting Information

for *Adv. Sci.*, DOI 10.1002/advs.202300148

Metabolome-Based Genome-Wide Association Study of Duck Meat Leads to Novel Genetic and Biochemical Insights

Dapeng Liu, He Zhang, Youyou Yang, Tong Liu, Zhanbao Guo, Wenlei Fan, Zhen Wang, Xinting Yang, Bo Zhang, Hongfei Liu, Hehe Tang, Daxin Yu, Simeng Yu, Kai Gai, Qiming Mou, Junting Cao, Jian Hu, Jing Tang, Shuisheng Hou and Zhengkui Zhou**

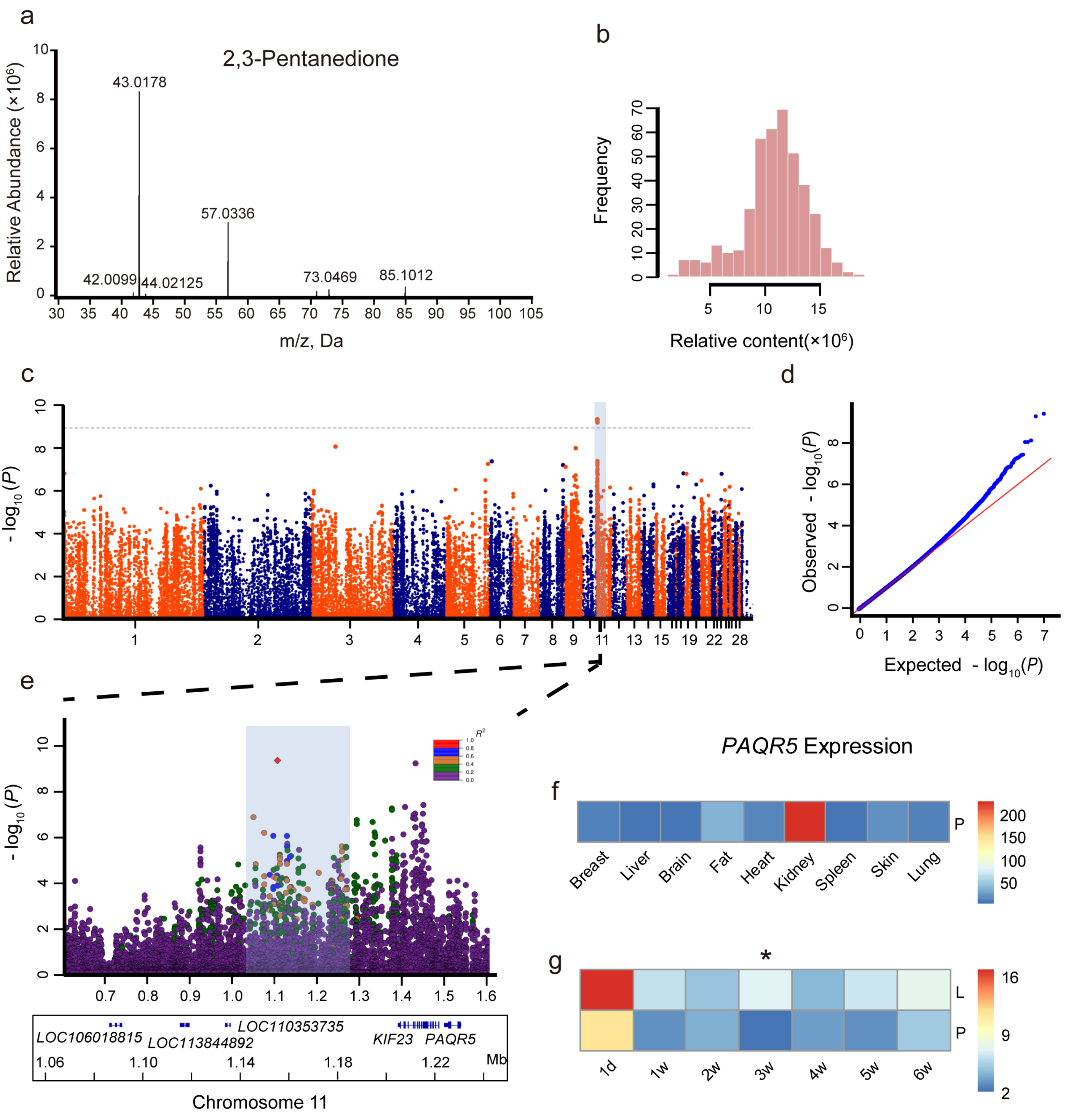

a
2,3-Pentanedione
Relative Abundance (×10⁶)
43.0178
57.0336
42.0099
44.02125
73.0469
85.1012
m/z, Da
b
Frequency
Relative content(×10⁶)
c
- log10(P)
d
Observed - log10(P)
Expected - log10(P)
e
- log10(P)
LOC106018815
LOC113844892
LOC110353735
KIF23
PAQR5
Mb
Chromosome 11
PAQR5 Expression
f
Breast
Liver
Brain
Fat
Heart
Kidney
Spleen
Skin
Lung
P
g
L
P
1d
1w
2w
3w
4w
5w
6w

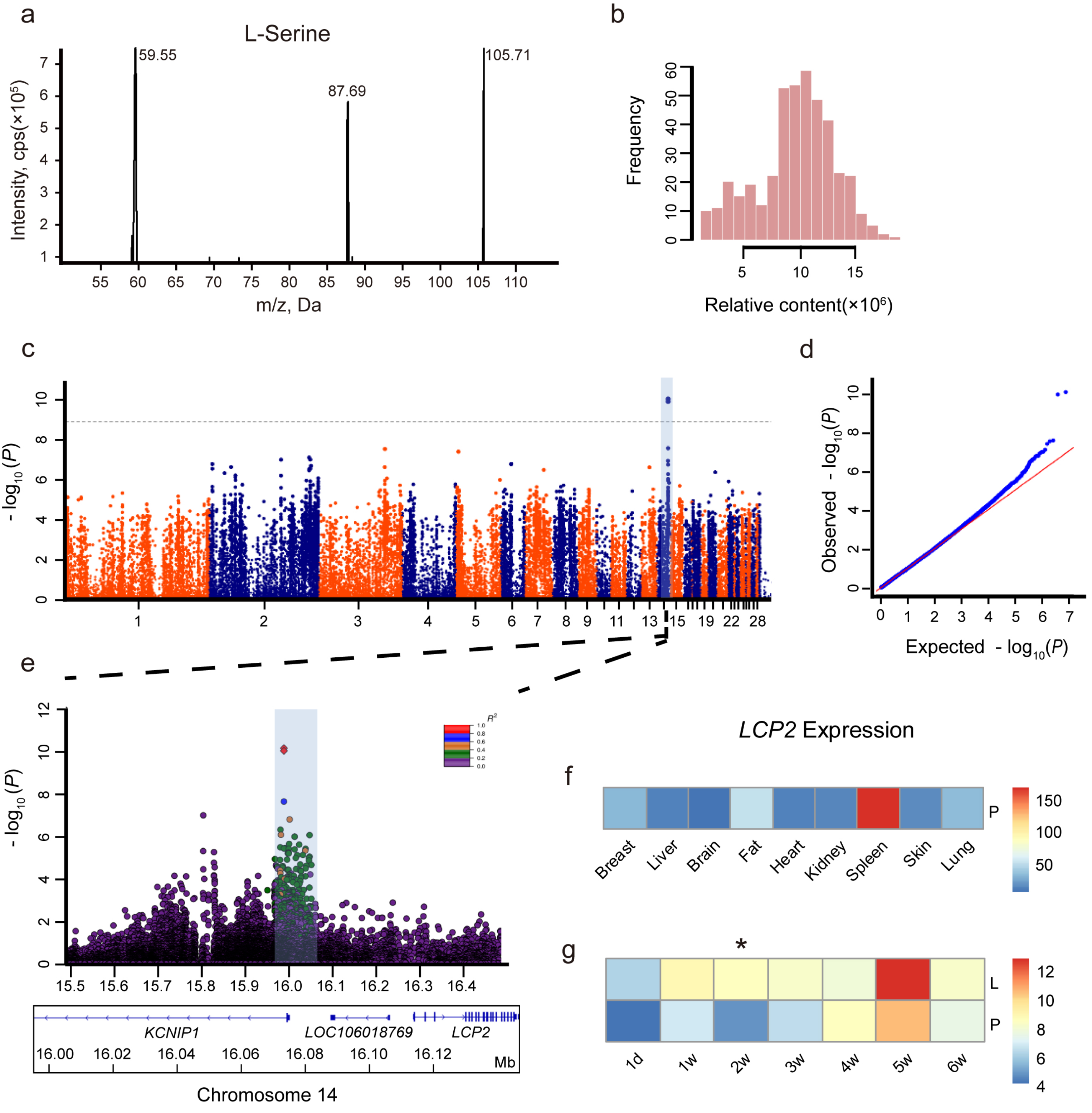
a
L-Serine
59.55
87.69
105.71
Intensity, cps(×10⁵)
m/z, Da
b
Frequency
Relative content(×10⁶)
c
- log10(P)
d
Observed - log10(P)
Expected - log10(P)
e
KCNIP1
LOC106018769
LCP2
Mb
Chromosome 14
LCP2 Expression
f
Breast
Liver
Brain
Fat
Heart
Kidney
Spleen
Skin
Lung
g
1d
1w
2w
3w
4w
5w
6w
L
P

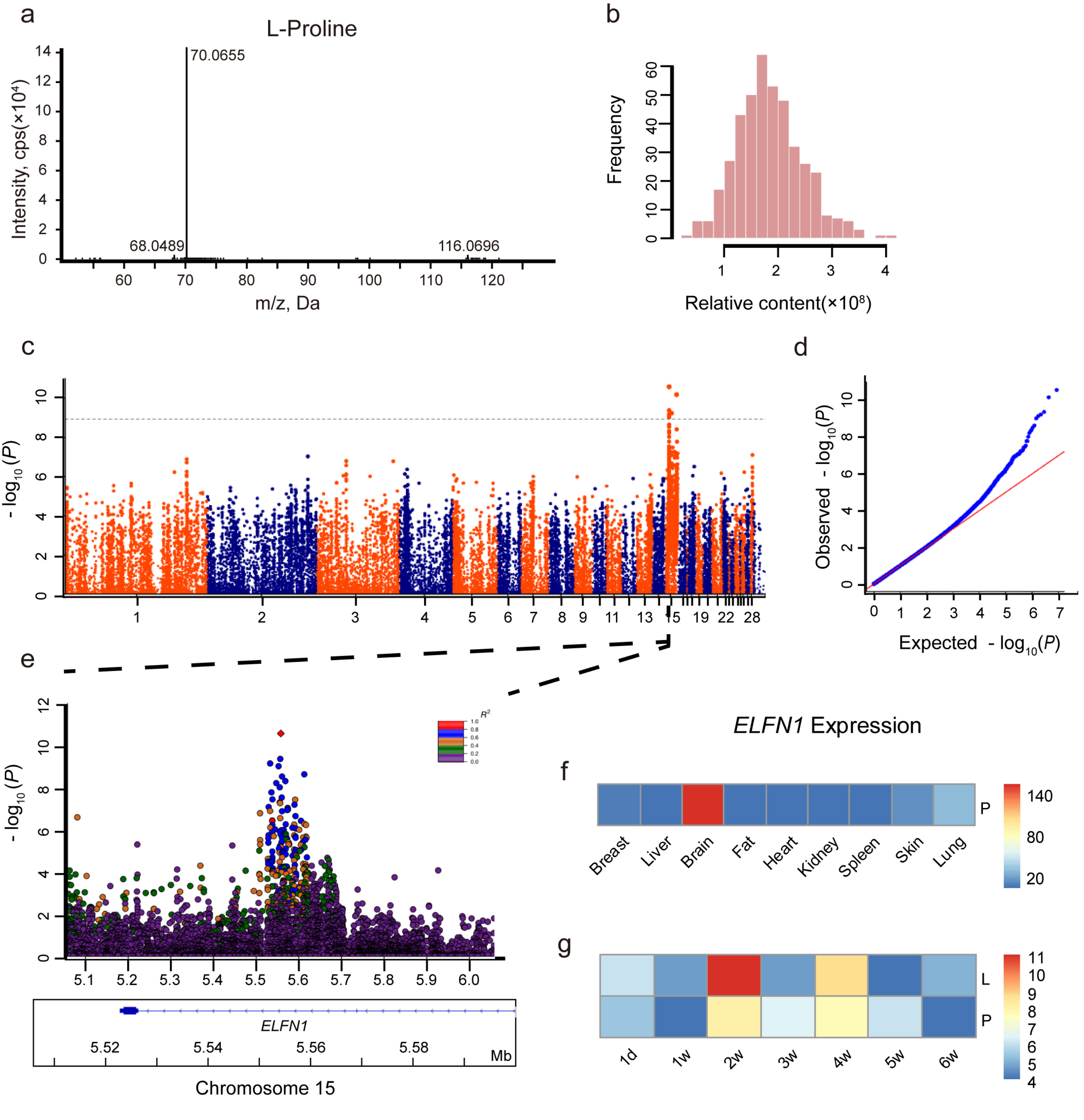

a
L-Proline
Intensity, cps(×10⁴)
70.0655
68.0489
116.0696
m/z, Da
b
Frequency
Relative content(×10⁸)
c
- log10 (P)
d
Observed - log10(P)
Expected - log10(P)
e
- log10 (P)
R²
ELFN1
Mb
Chromosome 15
ELFN1 Expression
f
Breast
Liver
Brain
Fat
Heart
Kidney
Spleen
Skin
Lung
P
g
1d
1w
2w
3w
4w
5w
6w
L
P

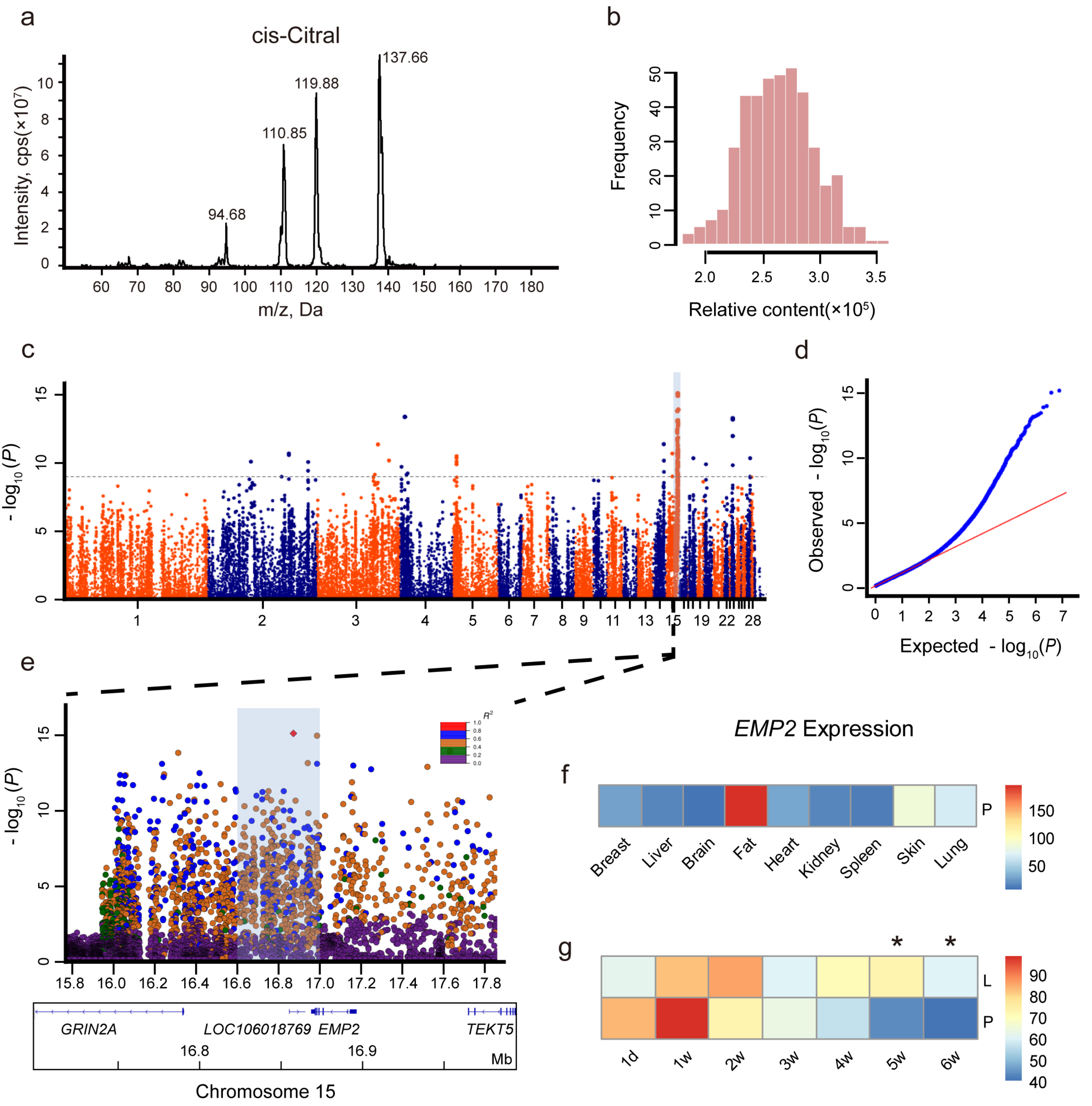
a
cis-Citral
Intensity, cps(×10^7)
137.66
119.88
110.85
94.68
m/z, Da
b
Frequency
Relative content(×10^5)
c
- log10(P)
d
Observed - log10(P)
Expected - log10(P)
e
- log10(P)
R^2
GRIN2A
LOC106018769
EMP2
TEKT5
Mb
Chromosome 15
EMP2 Expression
f
Breast
Liver
Brain
Fat
Heart
Kidney
Spleen
Skin
Lung
P
g
L
P
1d
1w
2w
3w
4w
5w
6w

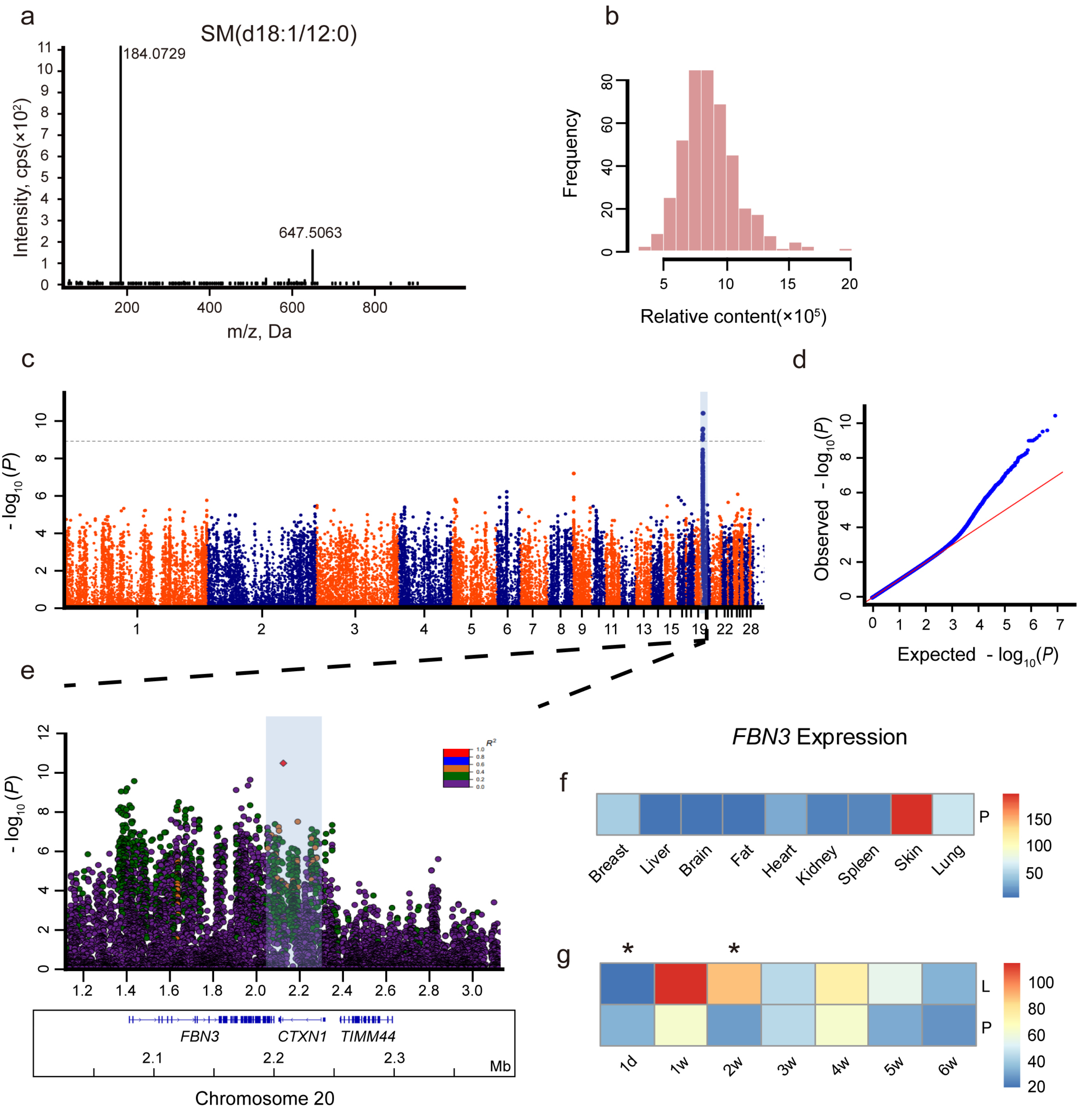

a
SM(d18:1/12:0)
184.0729
647.5063
Intensity, cps(×10²)
m/z, Da
b
Frequency
Relative content(×10⁵)
c
- log10(P)
d
Observed - log10(P)
Expected - log10(P)
e
- log10(P)
R²
FBN3
CTXN1
TIMM44
Mb
Chromosome 20
FBN3 Expression
f
Breast
Liver
Brain
Fat
Heart
Kidney
Spleen
Skin
Lung
P
g
L
P
1d
1w
2w
3w
4w
5w
6w

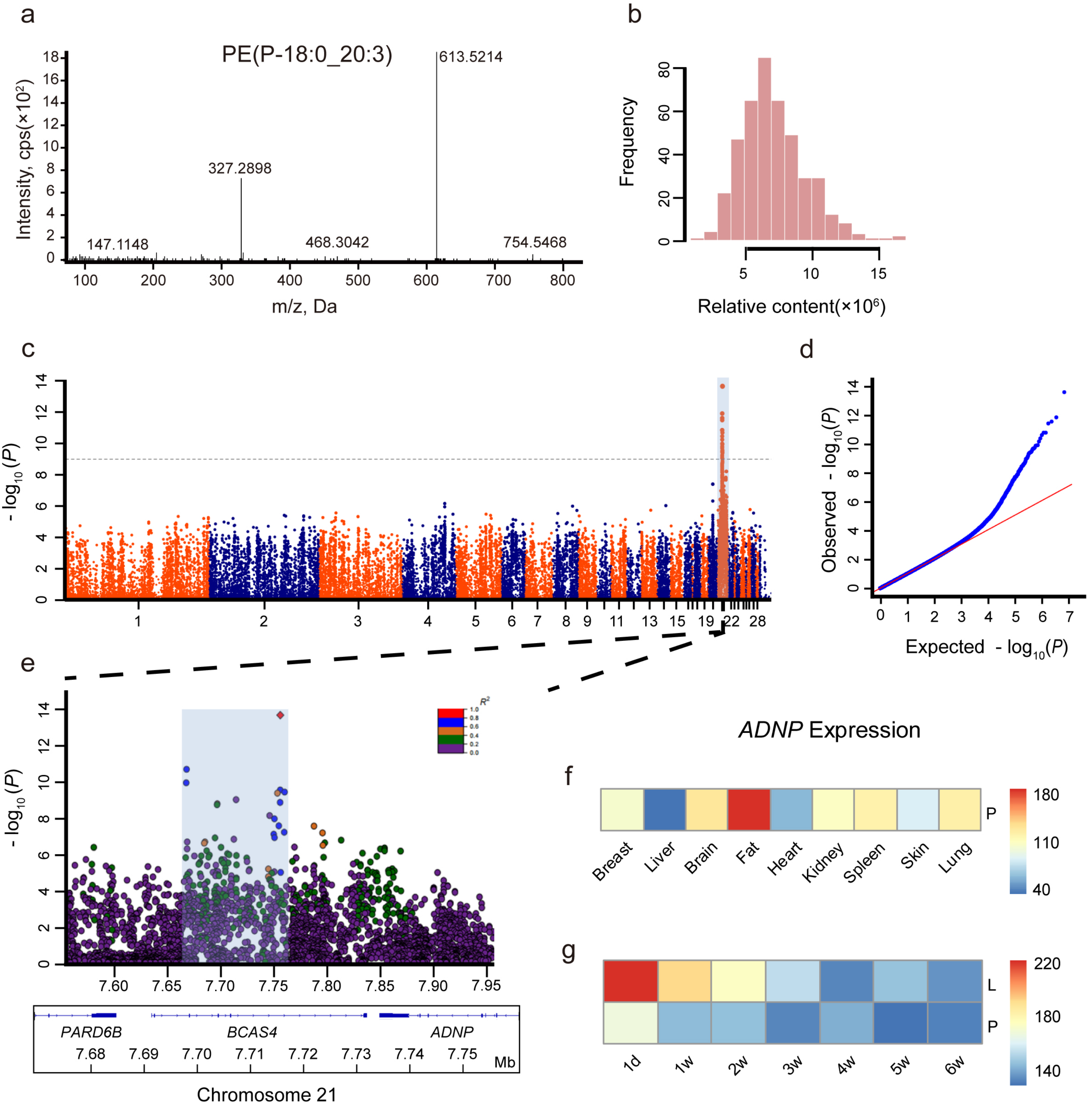

a
PE(P-18:0_20:3)
Intensity, cps(×10²)
613.5214
327.2898
147.1148
468.3042
754.5468
m/z, Da
b
Frequency
Relative content(×10⁶)
c
- log10(P)
d
Observed - log10(P)
Expected - log10(P)
e
PARD6B
BCAS4
ADNP
Mb
Chromosome 21
ADNP Expression
f
Breast
Liver
Brain
Fat
Heart
Kidney
Spleen
Skin
Lung
P
g
1d
1w
2w
3w
4w
5w
6w
L
P

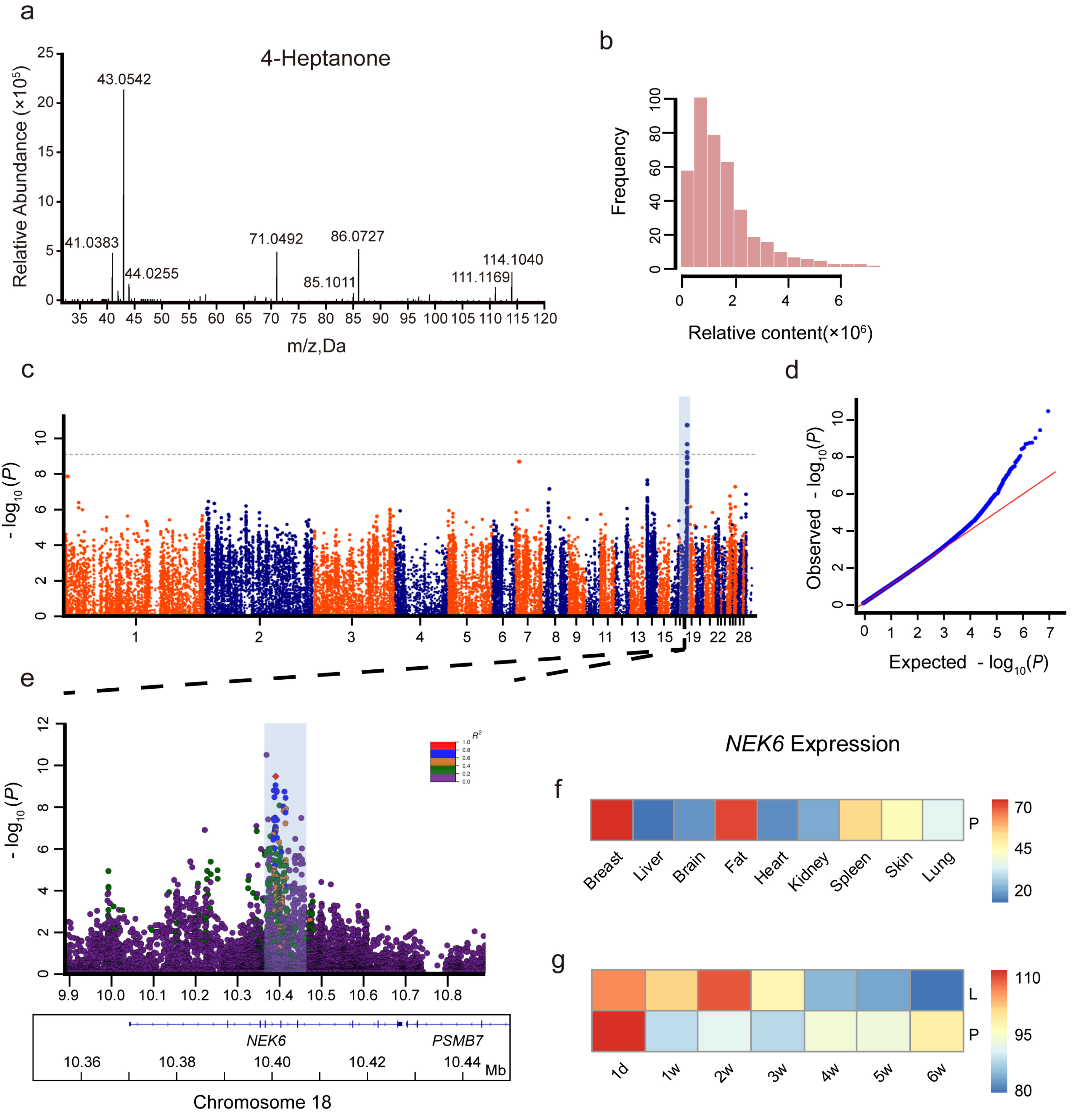
a
4-Heptanone
Relative Abundance (×10^5)
43.0542
41.0383
44.0255
71.0492
86.0727
85.1011
114.1040
111.1169
m/z,Da
b
Frequency
Relative content(×10^6)
c
- log10(P)
d
Observed - log10(P)
Expected - log10(P)
e
- log10(P)
R²
NEK6
PSMB7
Mb
Chromosome 18
NEK6 Expression
f
Breast
Liver
Brain
Fat
Heart
Kidney
Spleen
Skin
Lung
P
g
L
P
1d
1w
2w
3w
4w
5w
6w

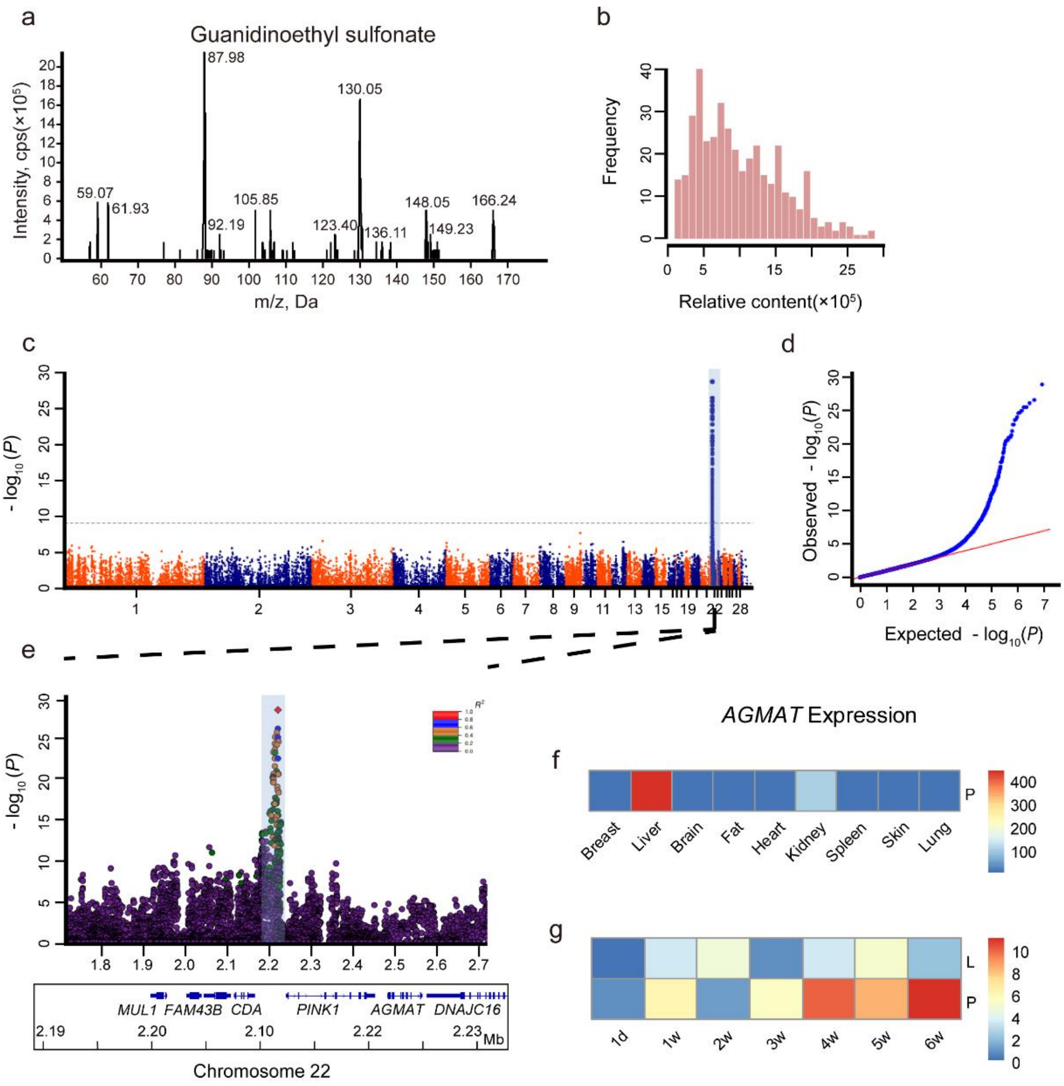

Supplementary Figure 12 The mGWAS results identified 43 candidate genes modulating metabolites of potential muscle physiological or nutritional importance.

(a) A histogram of the phenotype in the population. (b) LC-MS/MS fragmentation of hydrophilic metabolites, lipids and volatiles. (c) Manhattan plots of mGWAS of hydrophilic metabolites, lipids and volatiles. (d) Q-Q plot of the expected null distribution and the observed p-value using the mixed linear model. (e) Regional plots for the candidate region based on mGWAS results. (f) Multi-tissue gene expression profiling in Pekin duck (P) and Mallard (M). (g) Gene expression profiling between Pekin duck (P) and Liancheng duck (L) at seven development stages. * $P<0.05$, ** $P<0.01$, *** $P<0.001$.

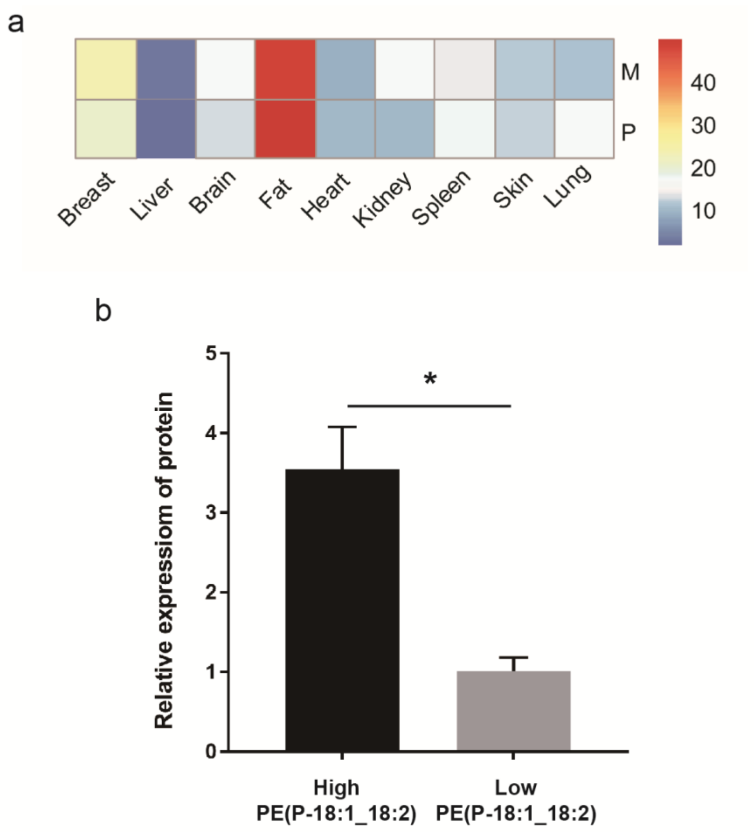

Supplementary Figure 13 The levels of *TMEM189* gene and protein expression. (a) Multi-tissue of *TMEM189* gene expression profiling in Pekin duck (P) and Mallard (M). (b) Relative expression of TMEM189 protein in the high PE (P-18:1_18:2) and low PE (P-18:1_18:2) content. * $P < 0.05$

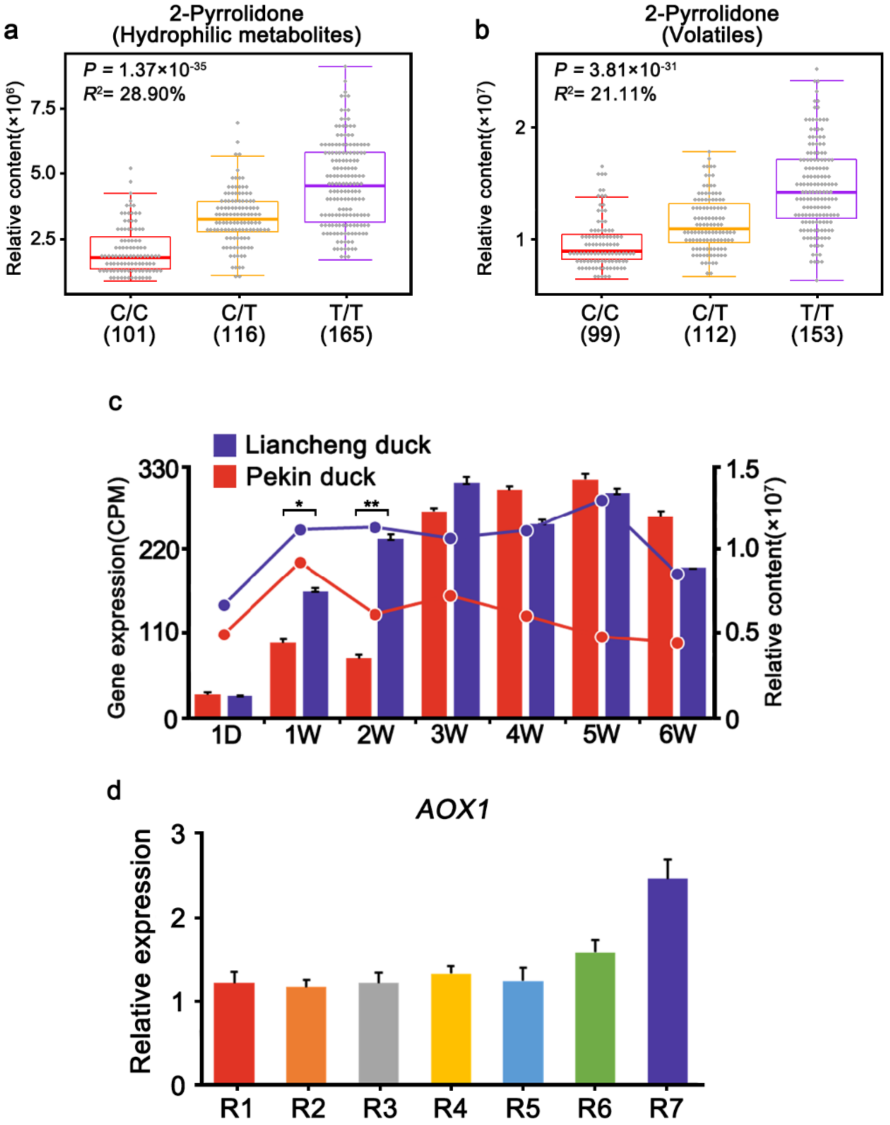

Supplementary Figure 14 Box plot of 2-pyrrolidone content of three genotypes at SNP Chr7: 4,810,144 and *AOX1* gene expression at seven developmental stages.

(a) 2-pyrrolidone content in hydrophilic metabolites. (b) 2-pyrrolidone content in volatiles. (c) 2-Pyrrolidone (hydrophilic metabolite) content changes corresponding to differentially expressed *AOX1* at 7 developmental stages, Red bars refer to Pekin duck, purple bars refer to Liancheng duck, * $P<0.05$, ** $P<0.01$. (d) The *AOX1* gene expression showed an increasing trend from R1 to R7.

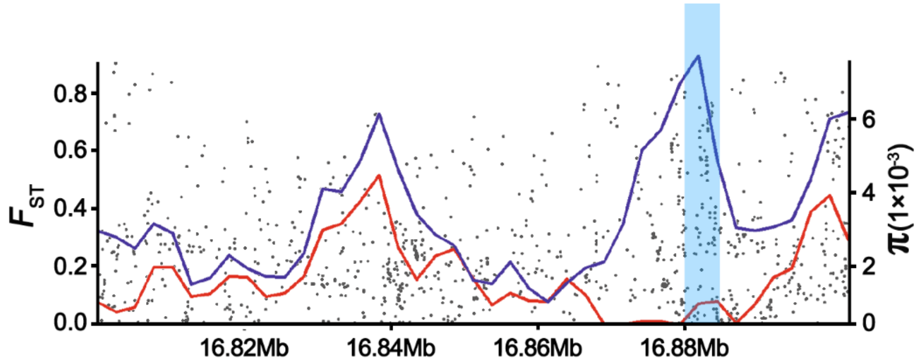

Supplementary Figure 15 Fixation index (F_{ST}, gray dot) and ratio of nucleotide diversity (π, 5 kb window, 2.5 kb step) for Chr2: 16.80 Mbp-16.90 Mbp SNPs between Pekin duck (red line) and Liancheng duck (blue line).

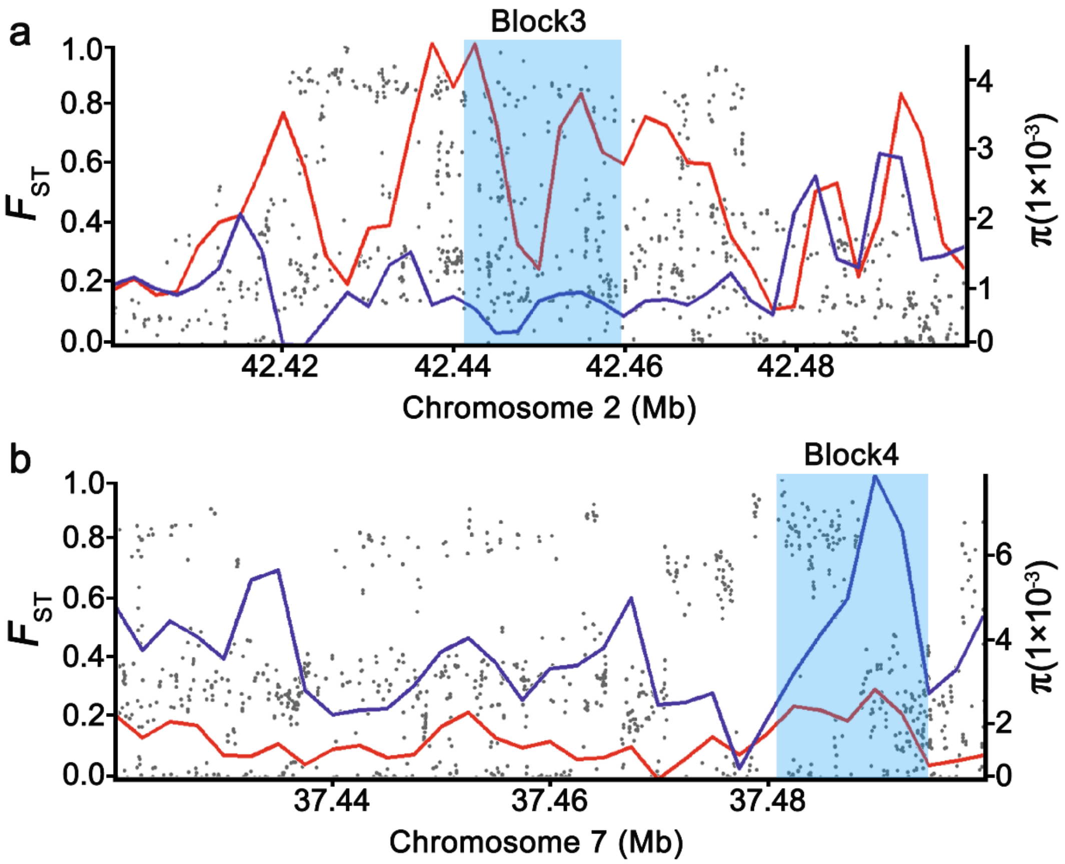

Supplementary Figure 16 Fixation index (*Fst*) and ratio of nucleotide diversity (π, 5kb window, 2.5kb step) for candidate region of Chr2 and Chr7 between Pekin duck and Liancheng duck.

(a) The region of Chr2:42.40 Mbp-42.50 Mbp. (b) The region of Chr7:37.40 Mbp-37.52 Mbp. Fixation index (F_{ST}, grey dot), Pekin duck (red line) and Liancheng duck (blue line).

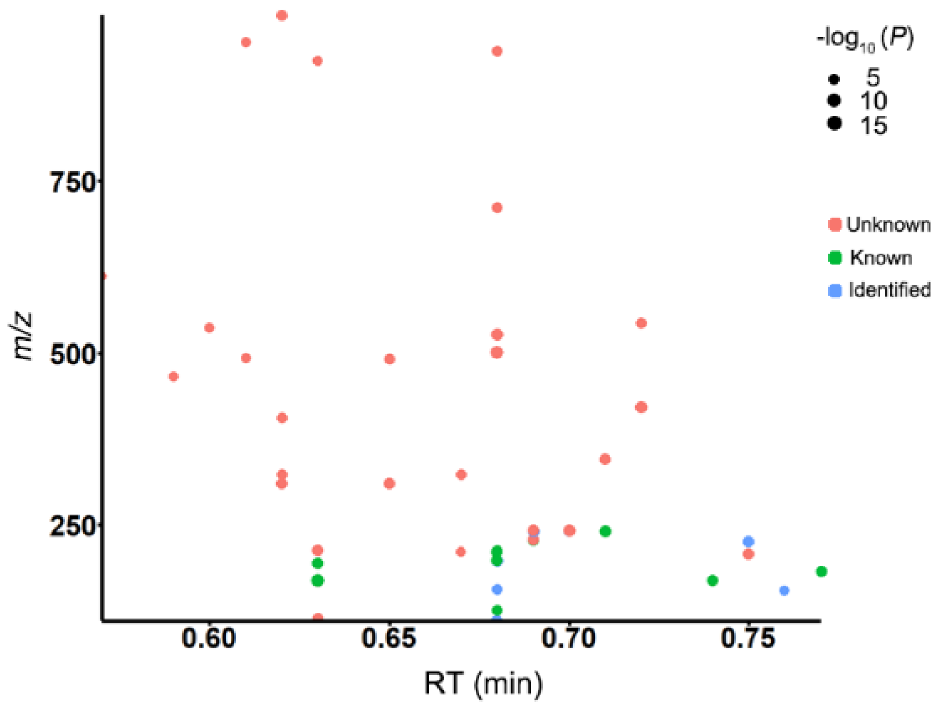

Supplementary Figure 17 49 carnosine and related metabolites in the m/z–retention time (RT) plane, a group of unidentified features sharing the RT region with histidine and its derivatives.

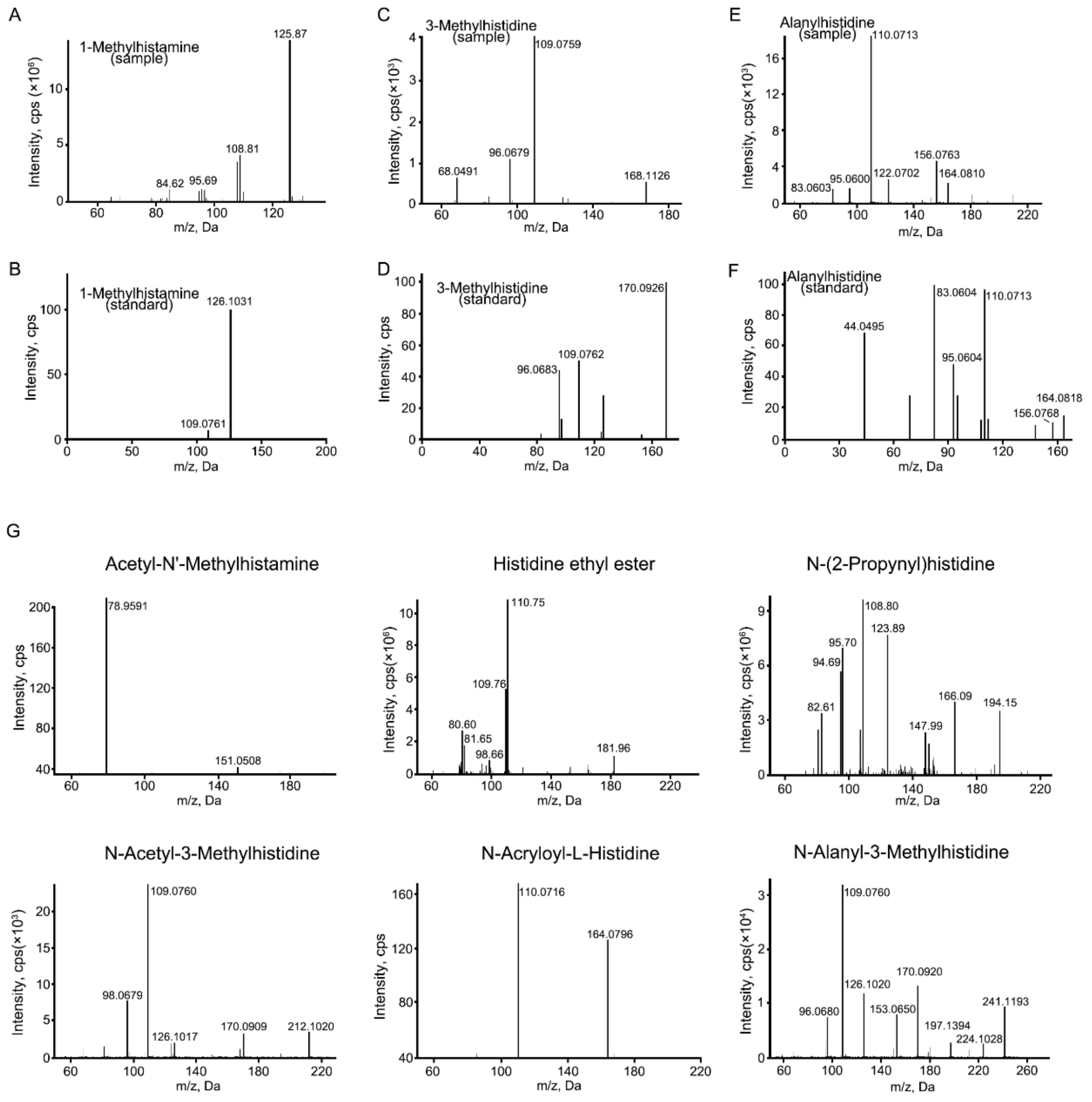

Supplementary Figure 18 9 unknown metabolites were annotated by mGWAS results and verified by high-resolution mass spectrometry.

(a)(b) Mass peak and spectrum of 1-Methylhistamine in meat sample and commercial standard. (c)(d) Mass peak and spectrum of 3-Methylhistidine in meat sample and commercial standard. (e)(f) Mass peak and spectrum of Alanylhistidine in meat sample and commercial standard. (g) Mass peak and spectrum of the other six unknown meatbolites.

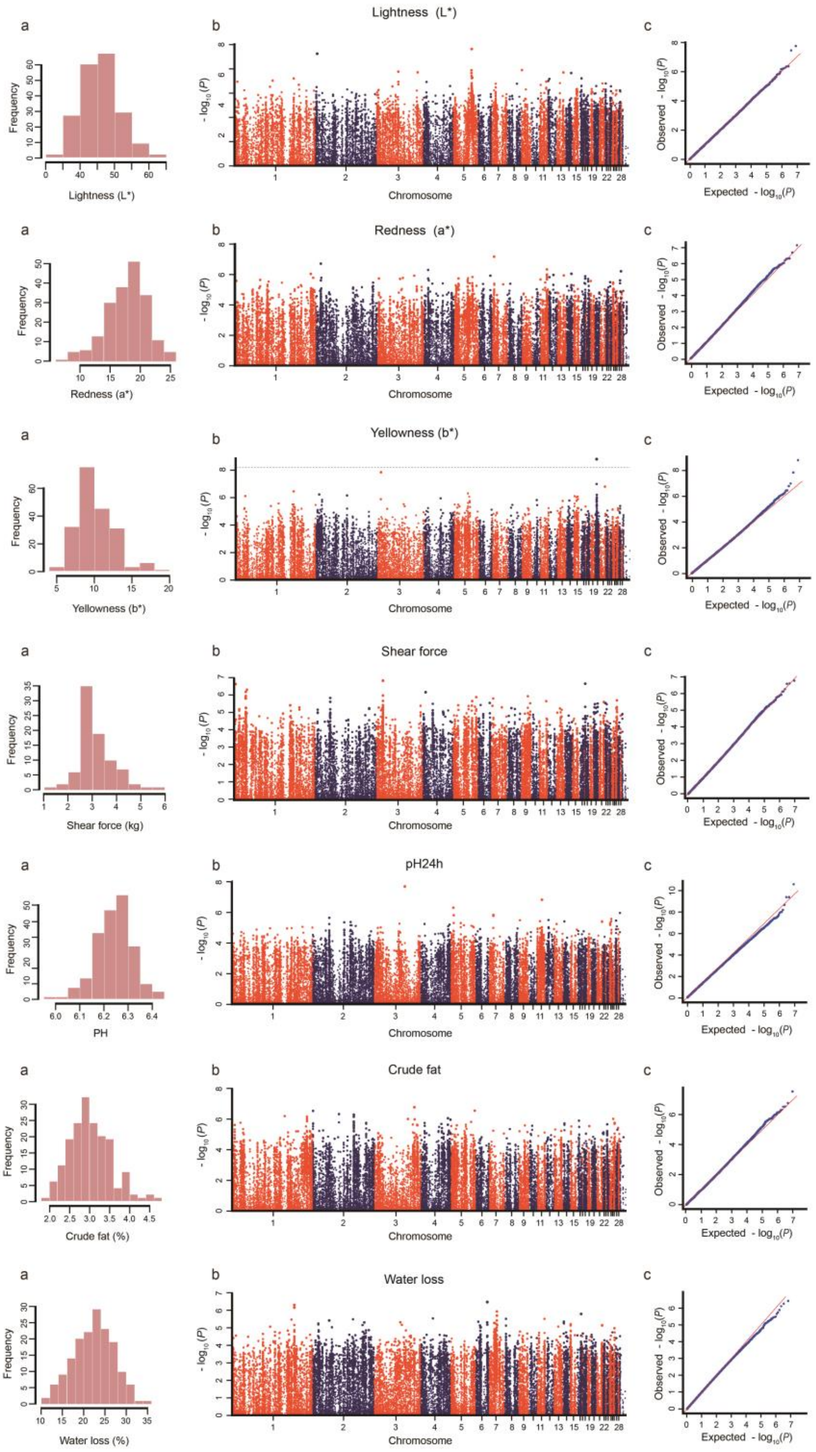

Supplementary Figure 19 GWAS results of 7 meat quality traits (lightness (L*), redness (a*), yellowness (b*), shear force, pH_{24h}, crude fat, and water loss).

(a) A histogram of the phenotype in this population. (b) Manhattan plots of GWAS results for meat quality traits. (c) Q-Q plot of the expected null distribution and the observed P value using the mixed linear model.

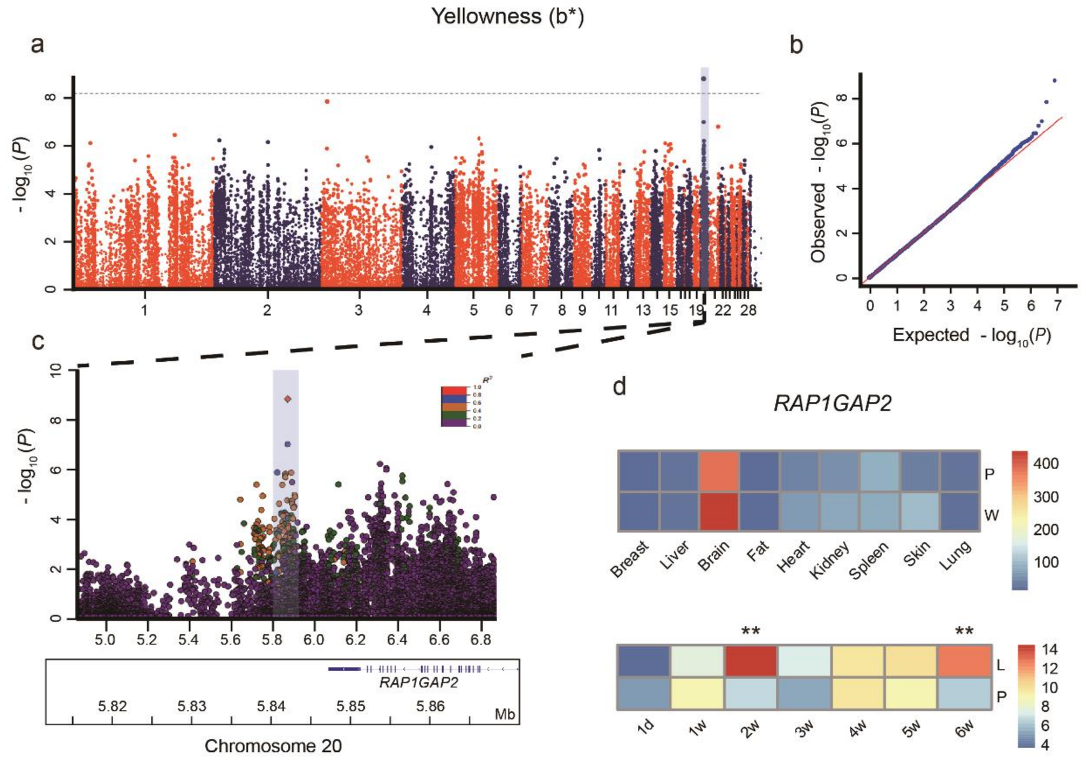

Supplementary Figure 20 The GWAS results showed that *RAP1GAP2* was significantly associated with yellowness (b*).

(a) Manhattan plots of mGWAS results for meat color (b*). (b) Q-Q plot of the expected null distribution and the observed P value using the mixed linear model. (c) Regional plots for the candidate region based on GWAS results. (d) Multi-tissue *RAP1GAP2* gene expression in Pekin duck (P) and *RAP1GAP2* gene expression in Pekin duck (P) and Liancheng duck (L) at seven developmental stages. * $P<0.05$, ** $P<0.01$, *** $P<0.001$.

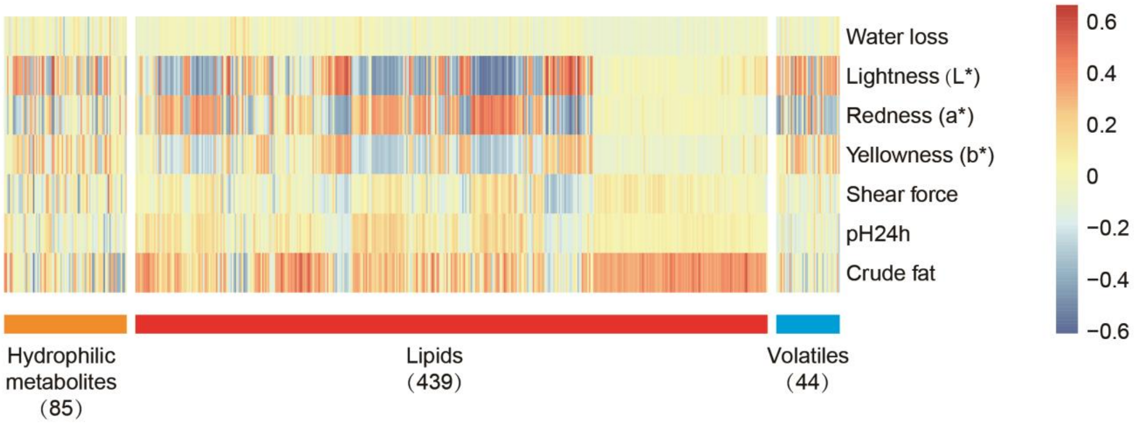

Supplementary Figure 21 A heatmap depicting Pearson's correlation between metabolites and meat quality traits**.**

Correlation ($r > 0.3$, or $r < -0.3$) between 7 meat quality traits (water loss, lightness (L*), redness (a*), yellowness (b*), shear force, pH_{24h}, and crude fat) and hydrophilic metabolites, lipids and volatiles.

Supplementary text

The detail procedure for meat quality phenotype determination

Meat color

The meat color of duck breast including lightness (L*), redness (a*), and yellowness (b*) was obtained by measuring randomly 3 positions of each sample with chromameter (CR-400, Minolta Camera Co., Osaka, Japan). The measurement of each position was repeated three times to obtain an average value.

PH_{24h}

The pH values of breast muscle were measured at 24 hours post mortem using a digital pH meter (HI99163, Dortmund, Italy). The pH detection probe was inserted into samples at a depth of 0.5 cm and the average pH value of each sample was calculated by taking 3 measurements in 3 different areas.

Shear force

Cooked breast meat samples were cut parallel to the direction of the muscle fibres, shaped to a size of 3 cm × 1 cm × 1 cm (length × width × thickness) and the shear force values of samples were measured using TA-XTplus texture analyzer (Texture Technologies Corp, Hamilton, MA, USA). Each sample was sheared in 2 areas to calculate the mean.

Water loss

The breast muscle was cut into 2 cm thick slices and squeezed under TA-XTplus texture analyzer (Texture Technologies Corp, Hamilton, MA, USA) to measure the weight of meat after squeezing. The result from each sample was recorded and water loss (WL) was calculated as: WL (%) = (original sample weight - squeezed sample weight) (g)/ original meat weight (g) ×100.

Crude fat

The routine Soxhlet extractor method was utilized to measure the crude fat of the breast muscle. All breast muscle samples were dried in the freeze dryer and then grinded into powder. Subsequently, approximately 1.0 g of each muscle powder sample was wrapped with a marked filter paper and placed in the extraction tube of the Soxhlet

extractor. Afterwards, 100 mL of petroleum ether was added into each extraction tube and samples was extracted at 70 °C for 12 hours. Finally, the samples were baked in an oven at 105 °C for 4 h and then weighed to determine the fat weight. The percentage of crude fat was calculated in the absolute dry state of the sample.
